# Sets2Networks: network inference from repeated observations of sets

**DOI:** 10.1186/1752-0509-6-89

**Published:** 2012-07-23

**Authors:** Neil R Clark, Ruth Dannenfelser, Christopher M Tan, Michael E Komosinski, Avi Ma'ayan

**Affiliations:** 1 Department of Pharmacology and Systems Therapeutics, Systems Biology Center of New York (SBCNY), Mount Sinai School of Medicine, One Gustave L. Levy Place, Box 1215, New York, NY, 10029, USA

## Abstract

**Background:**

The skeleton of complex systems can be represented as networks where vertices represent entities, and edges represent the relations between these entities. Often it is impossible, or expensive, to determine the network structure by experimental validation of the binary interactions between every vertex pair. It is usually more practical to infer the network from surrogate observations. Network inference is the process by which an underlying network of relations between entities is determined from indirect evidence. While many algorithms have been developed to infer networks from quantitative data, less attention has been paid to methods which infer networks from repeated co-occurrence of entities in related sets. This type of data is ubiquitous in the field of systems biology and in other areas of complex systems research. Hence, such methods would be of great utility and value.

**Results:**

Here we present a general method for network inference from repeated observations of sets of related entities. Given experimental observations of such sets, we infer the underlying network connecting these entities by generating an ensemble of networks consistent with the data. The frequency of occurrence of a given link throughout this ensemble is interpreted as the probability that the link is present in the underlying real network conditioned on the data. Exponential random graphs are used to generate and sample the ensemble of consistent networks, and we take an algorithmic approach to numerically execute the inference method. The effectiveness of the method is demonstrated on synthetic data before employing this inference approach to problems in systems biology and systems pharmacology, as well as to construct a co-authorship collaboration network. We predict direct protein-protein interactions from high-throughput mass-spectrometry proteomics, integrate data from Chip-seq and loss-of-function/gain-of-function followed by expression data to infer a network of associations between pluripotency regulators, extract a network that connects 53 cancer drugs to each other and to 34 severe adverse events by mining the FDA’s Adverse Events Reporting Systems (AERS), and construct a co-authorship network that connects Mount Sinai School of Medicine investigators. The predicted networks and online software to create networks from entity-set libraries are provided online at http://www.maayanlab.net/S2N.

**Conclusions:**

The network inference method presented here can be applied to resolve different types of networks in current systems biology and systems pharmacology as well as in other fields of research.

## Background

The skeleton of complex systems can be represented as a network where vertices represent entities and edges the relations between these entities. Often it is impossible, or expensive, to determine the network structure by experimental validation of all the interactions between all the vertices. It is usually more practical to infer the network from surrogate observations. Uncovering the relations between entities from indirect evidence is known as the problem of network inference or reverse engineering of networks from data. While many algorithms have been developed to infer networks from quantitative data of systems’ entities states, less attention has been placed on methods to infer networks from repeated observations of related sets. There are many cases in which groups or clusters of interrelated entities are known or can be observed experimentally. Typically such information is much more readily accessible than direct evidence of pair-wise interactions or even quantitative information about the entities under different conditions or time points. Each of these sets of related entities provides some information about the connectivity of the underlying network, and it would be of value to be able to utilize this information to resolve the connectivity of the underlying network connecting these entities. This inference process applies to a general class of inference problem of broad applicability; however, our motivation comes from problems in systems biology and systems pharmacology.

The tide of high-throughput biological data makes the inference of biological networks both more necessary and possible. There have been a number of success stories in the application of these methods to understand real biological phenomena. However, the multitude of components in biological molecular intracellular systems and their combinatorial interactions means that the possible networks that are consistent with observed data are astronomically large. Since we cannot directly observe many components of this system at once, and methods to profile binary interactions are expensive and laborious, the network remains under-determined; there are many networks which can equally well explain the observed data. However, in recent years the ability to sequence DNA, RNA and protein, together with the accumulation of prior knowledge about functional and physical relationships between genes and proteins, lends itself to a better ability to infer the underlying networks that govern the phenotype of mammalian cells.

At the same time, a new field is emerging called systems pharmacology. Systems pharmacology aims to integrate knowledge about drugs, drug-drug interactions, drug interactions with cells and organs, and drug relations to adverse events and desired effects in individual patients [1]. However, mining such data from various sources is challenging.

Current network inference methods employ a number of different strategies to reduce the search space of network structure solutions. Naturally, each strategy makes certain compromises and assumptions and has particular advantages and limitations [2]. While methods to infer networks from quantitative biological data have been popular, in particular methods such as Bayesian networks inference [3] and network inference based on mutual information [4], there has been less focus in systems biology on inferring co-occurence networks from sets of related entities and the applications thereof, while such an approach has been successfully applied more in other fields [5].

In most high-throughput (HT) methods that collect molecular biological data from cells, the underlying network is not known. Typically subsets of related molecular components are observed. For example, groups of co-expressed genes across different samples and contexts may be identified from transcriptomics data. Another example is groups of proteins which are listed in pull-down proteomics experiments that use immunoprecipitation followed by mass spectrometry (IP/MS) for protein complex identification. The identified genes or proteins may be regarded as the vertices of the underlying gene regulatory, cell-signaling or protein-protein interaction (PPI) networks. Extraction of the underlying network from such data can be achieved in many ways. In addition, the popular method of gene set enrichment analysis (GSEA) [6] uses libraries of related gene sets stored in the gene matrix transposed (GMT) format. In principle, and in most cases, within gene set libraries, each subset of related genes can provide some coarse local information about the connectivity of an underlying molecular network. As these gene sets accumulate, the underlying molecular networks that regulate mammalian cells may be resolved based on co-occurrence of genes within sets. We aim to employ the accumulation of course local information, from high-throughput experiments, to infer fine details of the global network. Here we give this idea a firm foundation and show that given accumulating subsets of related entities, the underlying network can be inferred with high fidelity, and if enough data is available, the network can be inferred completely. When the subsets of related entities do not provide enough information to infer the network with full confidence, we can extract all the possible information about the connectivity of the underlying network from the related set data to arrive at a best guess which can provide insights into the structure within the data.

The method we present here makes no *a priori* assumptions about the structure of the network or the nature of the edges. We begin our approach by grounding the solution in the fundamental principles of statistical mechanics. We numerically execute the solution on synthetic random networks to demonstrate its power. We then proceed to apply the method to real-world biological, clinical and social datasets in order to predict direct human protein-protein (PPI) interactions inferred from a recent HT-IP/MS dataset [7,8]; infer a network that connects mouse embryonic stem cell regulators based on data from ChIP-seq and loss-of-function/gain-of-function followed by expression data; learn a network that connects 53 cancer drugs and 34 common and severe side-effects extracted from thousands of patient records reported in the AERS database; and discover a network that connects authors based on co-authorship of publications listed on PubMed.

## Methods

### Exponential random graphs

Exponential Random Graphs Models (EGRMs) are a means of generating an ensemble of networks with prescribed statistical properties with the aim of modeling real-world networks. ERGMs were introduced by Holland and Leinhardt [9] and have a long history in complex systems research and are commonly applied in statistics and social science. ERGMs have been used for modeling complex brain networks [10] but their use in computational systems biology is scant. Park and Newman [11] showed that ERGMs are the natural extension of the fundamentals of statistical mechanics to network modeling. Here we adopt this formalism. The set of graphs *G* represents the sample space of graphs in the model, and, {*x*_*i*_}, *i =* 1 *… r*, represent a set of *r* empirical observations. Then a probability distribution *P*(*G*), can be defined over the elements *g* in *G*, such that the expectation value of x_i_ takes the empirical values. The best choice of probability distribution is the one which satisfies the empirical constraints,

(1) $$\sum_{g}P\left(g\right)x_{i}\left(g\right)=<x_{i}>\text{,}$$

while admitting no further information about the model graphs, which is achieved by maximizing the Gibbs entropy,

(2) $$S=-\sum_{g\in G}P\left(g\right)\ln P\left(g\right)\text{.}$$

This leads to a probability distribution which is the network equivalent of the Boltzman distribution,

(3) $$P\left(g\right)=\frac{e^{-H\left(g\right)}}{Z}$$

Where *H*(*g*) is the graph Hamiltonian function with Lagrange multipliers $\theta_{i}$,

(4) $$H\left(g\right)=\sum {}_{i}\theta_{i}x_{i}\left(g\right)$$

and *Z* is the partition function over the set of graphs *G*,

(5) $$z=\sum {}_{g}e^{-H\left(g\right)}$$

This probability distribution defines the exponential random graph model networks which obey the mean constrains of Equation 1, but which are otherwise maximally disordered.

### Dependence graphs

Pattison and Wasserman [12] showed that ERGMs can be generated in terms of dependence graphs. We shall briefly review these graphs here in the context of simply connected undirected networks, as they are relevant to the inference problem we address. The graphs which represent undirected simply connected networks can be represented by an adjacency matrix *g*_*ij*_, which is symmetric and has elements,

(6) $$g_{\mathrm{ij}}=\{\begin{array}{l} 1\;if\;v_{i}\;and\;v_{j}\;are\;connected\;by\;a\;vertex\;in\;G\\ 0\;otherwise \end{array}$$

The ERGMs define a probability distribution over *G*, and a model network, *g*_*ij*_ may be regarded as a realization of a random variable which is defined as a random selection from *G* over the probability distribution *P*. The dependence graph, *D,* has vertices which correspond to the edges in *G*_*ij*_. The edges of *D* connect vertices which are not conditionally independent. So the dependence graph identifies pairs of edges whose presence in the model network depends on each other. The Hammersley-Clifford theorem [13] then states that the model probability distribution, *P*(*G*), only depends on the complete subgraphs of the dependence graph.

### Inference approach

The inference problem addressed here is now phrased in terms of ERGMs. Given an unknown underlying network, *G*_*u*_, with vertices, *V*, we consider a set, *C*, composed of many subsets of the network’s vertices, $C=\left\{c_{i},c_{i}\subset V\right\}\text{,}\;i=1,\ldots ,N_{c}$, such that *C* is a field over the set of vertices. The sets *c*_*i*_ consist of vertices which are empirically observed to be related in the network, this could be for example, proteins identified in a mass-spectrometry proteomics pull-down, members of a cell signaling pathway, or co-authors of a publication. The central assumption is that each of the sets *c*_*i*_ identifies a locally connected subgraph of the underlying network. In these terms, the network inference problem we pose is thus: given the set of *N*_*c*_ subsets, *C*, and no other information, to what extent can we infer the underlying network *G*_*u*_?

To give a specific example, we may consider the results from HT-IP/MS, after appropriate filtering, as identifying a locally connected region of the underlying human protein-protein interactome network [7]. We hypothesize that we can resolve the underlying PPI network given enough observations of sets of proteins identified in different pull-downs. In these terms, the proteins would be represented by the vertices *v*_*i*_, and the list of proteins identified in each pull-down experiment would correspond to one element of *C*, while the underlying PPI network would be represented by *G*_*u*_. We are aware that we are searching for a static configuration of the network, whereas the underlying connectivity in complex systems, including PPIs and gene-regulatory networks, is dynamic as it may change over time and under different conditions.

We define our observable graph functions, *x*_*i*_(*G*) in the following way,

(7) $$x_{i}\left(G\right)=\{\begin{array}{l} 1\;\mathrm{if}\;\mathrm{the}\;\mathrm{elements}\;\mathrm{of}\;c_{i}\;\mathrm{form}\;\text{a}\;\mathrm{connected}\;\mathrm{subgraph}\;\mathrm{of}\;G\\ 0\;otherwise \end{array}$$

If we interpret each set as providing course local information on the connectivity of the underlying *G*_*u*_, such that we have a confidence, α, that the elements in each line are locally connected, then the constraints on the ensemble are the following,

(8) $$\sum_{G}P\left(G\right)x_{i}\left(G\right)=\alpha$$

in which case the maximum entropy probability distribution function *P*(*G*) identifies an ensemble of networks which have a connectivity consistent with the known input set *C* but which are otherwise maximally disordered, we shall refer to this as *G*_*ens*_. In our studies of the properties of our inference approach on synthetic networks we shall generate data which identifies locally connected regions of the underlying network and so we shall take the value of *α* = 1. When we apply our inference approach to data from systems biology the value of *α* will typically be less than unity as there will generally be only a finite degree of confidence in the connectivity of each local region identified in the data.

The above constraints leading to the probability distribution *P*(*G*) can also be seen in terms of a dependence graph, *D*, for the corresponding ERGM. Each set *c*_*i*_ contains vertices between which a set of edges are assumed to exist in the underlying network in order to form a locally connected subgraph. Over the ERGM ensemble, the presence of each of these edges is conditionally dependent upon the others. These edges form a complete subgraph of *D*, and therefore, according to the Hammersley-Clifford theorem, define the probability distribution *P*(*G*).

In the approach presented here we make no attempt to infer directionality, hence, we take the sample set, *G*, to consist of simple undirected graphs. The data contained in, *C*, constitutes an accumulation of course local information on the connectivity of the underlying network. Even with large amounts of this class of data the network often remains underdetermined, hence there exists a whole ensemble of networks which are consistent with the data. In order to infer finer details of the underlying network we adopt some of the philosophy of network modeling to take a sample of this ensemble, and then use the properties of this ensemble for inference. Here we take the sample numerically by using an algorithm which generates a random sample of the ensemble. The algorithm ensures that the sampled network has the local connectivity consistent with the data while minimizing the number of edges. The edge minimization ensures the strongest signal from the data in the sense that edges only appear in the ensemble as required by the data.

The algorithm works by generating a random sample, of size *N*_*g*_, of the ensemble of networks *G*_*ens*_ consistent with the data. According to the assumptions of the inference, the GMT file contains a number *N*_*C*_ of lines, *c*_*i*_*,* each of which consists of vertices which are locally connected in the underlying network. The algorithm first randomly permutes the order of the lines in the GMT file, then, starting with the empty graph which consists of the set of nodes (all distinct entities included in the data) and the empty set of edges *E*_*i*_ = {}, it builds a network by taking each line and introducing a minimal number of random links that connect the vertices in that line. The pseudo-code for the algorithm is then:

For i = 1 to *N*_*g*_

Randomly permute the order of the lines in the *GMT* file

*E*_*i*_ = {} (start with a graph with no links)

For j = 1 to *N*_*C*_

Randomly introduce a minimal number of edges between the vertices *c*_*i*_ such that they are connected, and continually append to the set *E*_*i*_

End For i

End For j

Calculate the mean adjacency matrix of the ensemble *G*_*ens*_

A sample of random networks generated in this fashion constitutes a random sample of *G*_*ens*_. The properties of this ensemble are then used to infer the underlying network *G*_*u*_. Specifically, we calculate the mean adjacency matrix over this ensemble, each element of which corresponds to the probability of the edge being present in a uniformly random draw from the ensemble; this is interpreted as being indicative of the accumulation of information on the presence of the edge in the underlying network.

### Analytical approximation

The algorithmic sampling of the ensemble *G*_*ens*_ becomes computationally demanding when inferring networks with many vertices, and large amounts of data. When applying the approach to infer biological networks from large high-throughput datasets which although sparse, can have thousands of vertices, we look for an efficient analytical approximation to the algorithmic solution. The approach we take is to analytically mimic the function of the fully executed algorithm that generates the networks, which are samples of *G*_*ens*_. The first order approximation is to treat each *c*_*i*_ as generating an independent minimally connected Bernoulli random graph, in which each edge has an independent and equal probability of appearing. Then the superposition of all the *c*_*i*_ may be regarded as a Bernoulli process in which each edge undergoes a series of Bernoulli trials with probabilities corresponding to the random graphs generated by each of the elements of *C*. In this approximation the probability that a given edge is present in *G*_*u*_ is given by,

(9) $$p_{\mathrm{ij}}=1-\Pi_{k}\left(1-\frac{2\alpha }{n_{ij,k}}\right)$$

Where *n*_*ij,k*_ is the size of the kth elements of *C* to which both vertices *v*_*i*_ and *v*_*j*_ belong.

### Alternative co-occurrence scoring methods

To compare the above methods to other approaches that can be used to construct networks from repeated observations of co-occurence of entities in related sets we compare our approach to two alternatives. A simple difference of proportions test to measure co-occurrence strength is applied as follows: For two given vertices, *A* and *B*, a contingency table can be constructed with *f(A and B)*, *f(A and not B)*, *f(not A and B)*, and *f(not A and not B)* where *f(X)* indicates the frequency of lines *c*_*i*_ in *C* where *X* is true. We then use the Chi-squared test of difference of proportions to test the significance of the co-occurrence. Then the probability of interaction between *A* and *B* is calculated as one minus the p-value. In addition, the simplest possible form of inference from co-occurrence whereby the probability of an edge between *A* and *B* is given by the ratio of the number of *c*_*i*_ in the GMT file in which both *A* and *B* occur to the total number of lines, *N*_*c*_, is also used as a comparison.

### Bias adjustment

While artificial random networks can be evenly and randomly sampled, real world datasets contain sampling biases where some vertices are measured more often. For example, well-studied genes/proteins appear in more publications and thus commonly have more reported interactions with other genes/proteins. This inhomogeneity of information throughout the network means that the inferred interactions are as sensitive to the frequency with which particular vertices are observed as to the strength and specificity of the edges between the vertices. In order to correct for this and calculate probabilities which are only sensitive to the strength of the interactions, we generate a null distribution for each edge probabilities under the hypothesis of no specific interaction. This is achieved by calculating the distribution of each edge-probability *p*_*ij*_ in the network after any information on the network connectivity has been destroyed: the GMT file data is randomly permuted, while preserving its structure by conserving the lengths of each set and the frequency of each element throughout the data, and allowing only one of each type in any given set/line, then all the edge weights are calculated. This process is repeated to generate the null distribution for each edge weight. By comparing the actual edge weight to this null distribution, under the hypothesis of no interaction, we obtain a p-value which quantifies the edge-probabilities after correction for the sampling bias. This p-value is then used as a measure of the strength of the interaction.

### Combining evidence from multiple GMT files to build a consensus network

We can combine several types of datasets stored in different GMT files to form a consensus network. By combining two or more different inferred network perspectives we may gain additional insight into the functional associations between related vertices. When combining data from several sources we rewrite equation 9 for the edge probabilities as:

(10) $$p_{i,j}=1-\left[\Pi_{k}\left(1-\frac{2\alpha }{n_{i,j,k}}\right)\right]\left[\Pi_{k\prime }\left(1-\frac{2\alpha \prime }{n_{i,j,k\prime }}\right)\right]\left[\ldots \right]\text{,}$$

where the terms in each square bracket come from each distinct data source and the Greek letters are the corresponding confidence parameters. To compute scores that consider the bias adjustment, the distribution of edge weights from randomly permuted GMT files are generated using the same equation and the same steps described above for inferring probabilities for edges for individual networks.

### Matthews correlation coefficient to evaluate inferred networks

To evaluate the quality of the predicted edges we use the Matthew's correlation coefficient (MCC). MCC is a balanced measure of the quality of binary classifications, and it is equal to unity when the classification is perfect; it is computed by the following equation:

(11) $$MCC=\frac{TP\times TN-FP\times FN}{\sqrt{\left(TP+FP\right)\left(TP+FN\right)\left(TN+FP\right)TN+FN}}$$

Where FP and FN are the false positives and negatives respectively, and TP and TN are the true positives and true negatives respectively.

## Results

### Inference of synthetic networks

**Figure 1:**
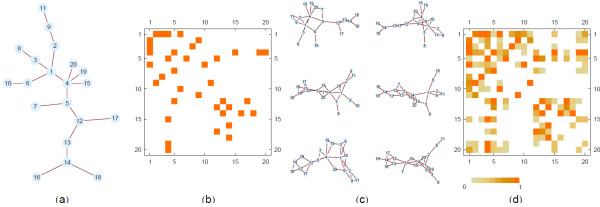
**Example of inferring a network from synthetic data.** (**A**) An arbitrary random graph serves as *G*_*u*_ which can be represented by an adjacency matrix, shown in (**B**). The field *C* is generated synthetically by performing short random walks on the graph to identify locally connected subgraphs. Using only the information contained in *C* the ensemble *G*_*ens*_ is algorithmically sampled, a few of the sampled graphs are shown in (**C**). An example of the mean adjacency matrix over *G*_*ens*_ is shown in (**D**).

**Figure 2:**
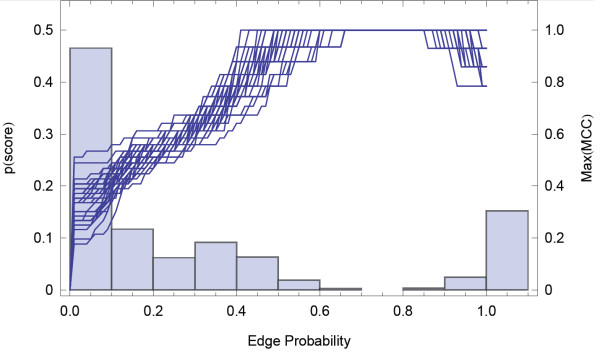
**Histogram of interaction probabilities (pale blue bars) and their corresponding MCC (deep blue lines) as a function of *p***_t_** for inferring ten synthetic networks of the same size (n = 50).**

First we investigate the quality of inference using our approach by applying it to synthetic test networks. We begin with a randomly generated connected graph serving as the underlying network *G*_*u*_ that we wish to infer (Figure 1A), which is also represented by its adjacency matrix (Figure 1B). The set *C* used to generate a GMT file is created by performing short random walks starting on random vertices. The idea is to infer *G*_*u*_ only using the information from *C*. The ensemble, *G*_*ens*_, is generated algorithmically by randomly introducing a minimum number of links in order to connect successive sets of vertices *c*_*i*_. A small selection of the calculated elements of *G*_*ens*_ is shown in Figure 1C. The mean adjacency matrix of this ensemble is then calculated (Figure 1D). The elements of the mean adjacency matrix are interpreted as probabilities that a given link is present in *G*_*u*_. Due to the binary nature of the edges, a comparison can be made between the underlying network *G*_*u*_ and the mean adjacency matrix, after the application of a threshold value of *p*_*t*_. A histogram of edge probabilities, *p*_*ij*_, shown in Figure 2, has a bimodal form which appears to distinguish between edges present in the underlying network (large mode) and edges not present in the underlying network (small mode). In addition, on the *x*-axis we plot the MCC as a function of the threshold *p*_*t*_. In other words, we plot the MCC where we cut the inferred adjacency matrix to decide which scores will constitute an edge. If we set a large threshold value there will be only few edges remaining in the network and thus many false negatives and lower MCC. Conversely, as the threshold is reduced to zero the network will tend to completeness and thus will include many false positives. There is a region of *p*_*t*_ where the similarity between the inferred network and the underlying network is greatest. This region corresponds to the peak of the MCC curve (Figure 2). 

The aim of applying the inference approach to such synthetic networks is to investigate the quality of the inference in a case where the underlying network is known. This investigation requires several steps. First, because the approach requires a random sample (of the consistent networks) we must investigate the convergence of the statistics deriving from this sample. Once the rate of convergence is known the required sample size *N*_*g*_ for a given degree of convergence is obtained and the method may be reliably applied. The next step is to examine the quality of inference and its dependence on the properties of the available data. Finally, with a view to the application of our method to problems in systems biology we examine the computational complexity and running time of our approach.

### Convergence of inferred networks

Our inference approach is based on the mean of a random sample of networks which are consistent with our data. The rate of convergence of this statistic depends on the properties of the data upon which the ensemble is based and so a general convergence rate does not exist. We can however derive an upper limit to the rate of convergence by considering the convergence in the worst-case-scenario. In this case there is no information on the connectivity of the underlying network, and therefore the largest possible ensemble of consistent networks and so the slowest rate of convergence. Here we derive the rate of convergence of the mean in this case as the upper limit for the rate of convergence.

In this worst-case every connected network with *n* edges is a consistent network. In a random sample of these networks a given edge has a probability $\frac{2}{n}$ of being present in any given network. Effectively, for a given edge, each sampled network constitutes a Bernoulli trial, and therefore in a random sample of *N*_*e*_ consistent networks, the total number of times any given edge is present, *X*, follows a Binomial distribution with *N*_*e*_ trials at a probability of success of $p=\frac{2}{n}$. If we use our sample to estimate this probability, $p_{\mathrm{est}}=\frac{X}{N_{e}}$, then the resulting estimate will be distributed by a (scaled) Binomial distribution with mean, *p*, and variance given by $\frac{Var\left(X\right)}{N_{e}^{2}}$, which can be written as, $\frac{p\left(1-p\right)}{N_{e}}$. The signal-to-noise ratio is sometimes written as the ratio of the mean to the variance, so in this case it is given by, $SNR=\sqrt{\frac{pN_{e}}{1-p}}$ which we can use to gauge the convergence by requiring that *N*_*e*_ is large enough to achieve a certain signal-to-noise ratio. Given that $p=\frac{2}{n}$, it means that the ensemble size must increase in proportion to the number of nodes n, in order to preserve a constant signal-to-noise ratio. The ensemble size required to achieve a given signal-to-noise ratio in this case can be written as,

(12) $$N_{e}=\left(\frac{n}{2}-1\right)SNR^{2}$$

and this is the formula used to determine the sample size throughout all applications of the algorithm presented here.

The above estimate can be used as an upper limit to the *N*_*e*_ required for the mean to converge to a given signal-to-noise ratio in the case of general GMT file data; this is because such GMT data file is more informative, hence the size of the ensemble of consistent networks is smaller, and a smaller sample is required for inferring the underlying network. We demonstrate this in a plot of the signal-to-noise ratio against the size of the ensemble for inference of a 20 node network (Figure 5).

**Figure 3:**
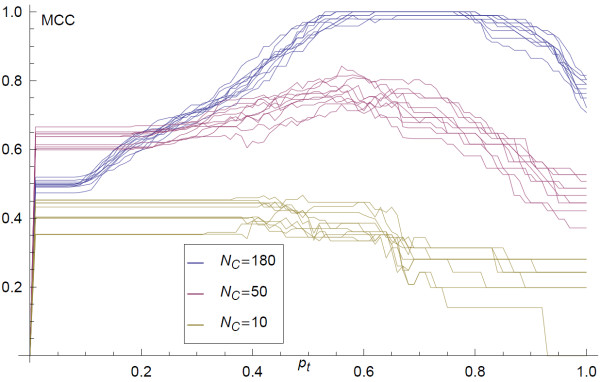
**MCC as a function of the threshold value *p***_***t***_** applied to the mean adjacency matrix calculated for ten synthetic network inferences from *C *with a number of subsets *N***_***C***_**= 180, 50, 10, where each of the connected subsets were generated by a random walk on the respective underlying synthetic networks * G***_***u ***_**of length 3.**

### Accuracy of inference

**Figure 4:**
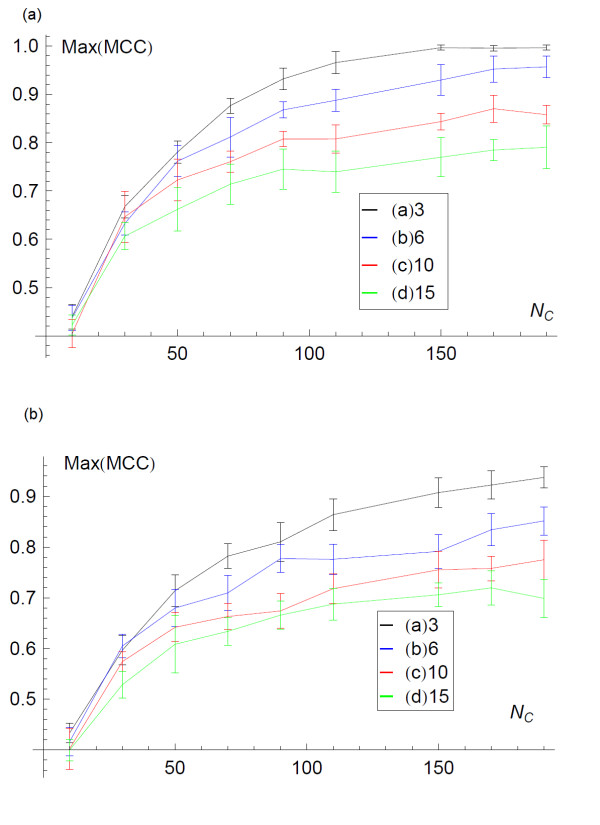
**The maximum value of the MCC over the full range of the threshold * p***_***t ***_**plotted against the size of the field, *N***_***C.***_ The curves show the resolution of the underlying network as *N*_*C*_ increases, where each *c*_*i*_ is generated by performing random walks of increasing length, as indicated in the legend. The upper figure derives from the algorithmic sampling while the lower figure derives from the analytic approximation.

Having quantified the convergence of the inferred network with the sample size *N*_*c*,_, the next consideration is how similar the inferred network is to the underlying network from which the data derives. The quality of the inference depends on the data contained in the GMT file. As the number of lines in the GMT file, *N*_*c*,_ increases, the information on the presence of edges accumulates and the inferred network becomes more similar to the underlying network. The length of the lines in the GMT file also influences the quality of the inference; larger lines provide coarser information on the connectivity of the network and thus are less informative. Finally, the more nodes are in an underlying network the greater the amount data required to infer its structure. Here, for a given number of n odes, *n*, in a minimally connected network we examine the quality of inference and how it depends on *N*_*c*,_ and the length of the lines in the GMT file. We begin by examining the dependence on the number of lines in the GMT file. We infer the structure of a minimally connected network with *n* = 50, from GMT files with a range of 10 to 180 lines. To quantify the quality of the inference we use the maximum value of the MCC. In Figure 6 we plot the maximum MCC against the number of lines in the GMT file, *N*_*c*,_, using four different methods of inference: 1) the full-algorithm 2) the analytic approximation 3) Chi-squared difference of proportions 4) simple co-occurrence. In each case we see that as *N*_*c*_ increases, i.e. with more data, the accuracy of the inference increases and tends towards ideal inference. This is when the inferred network is exactly the same as the underlying network. We also observe that the full algorithm described above is more accurate than the other methods, but the analytic approximation is also more accurate than more basic co-occurrence approaches (Figure 6). Finally we examine the dependence of the accuracy of inference upon the size of the sets (length of lines) in the GMT file. In Figure 4 we plot the mean quality of inference as measured by the maximum MCC. The four curves correspond to inference from synthetic GMT files with random walks of lengths of (a) 3, (b) 6, (c) 10, (d) and 15. Longer random walks result in GMT files with longer lines, and therefore coarser information on the connectivity of the underlying network. We see that each curve has the same tendency to increase the quality of inference with increased *N*_*c*_, however, the rate reduces with increasing size of set in GMT file, shorter lines. In this way we show that for coarser information (longer GMT file lines) more data is required to infer the network. 

**Figure 5:**
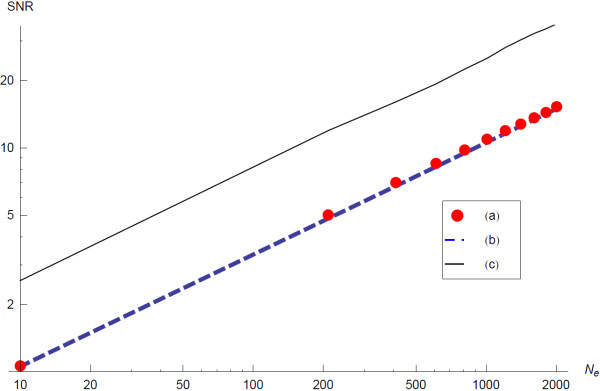
**Signal-to-noise ratio (SNR) plotted against the size of the ensemble for inference of a 20 vertices network.** (**a**) Mean SNR for edges inferred from a worst-case GMT file over a range of sample-sizes *N*_*e*_ (averaged over 40 inferred networks). (**b**) Expected SNR for the worst-case GMT file. (**c**) SNR in the case of GMT files generated by performing 100 short random walks (length 3).

**Figure 6:**
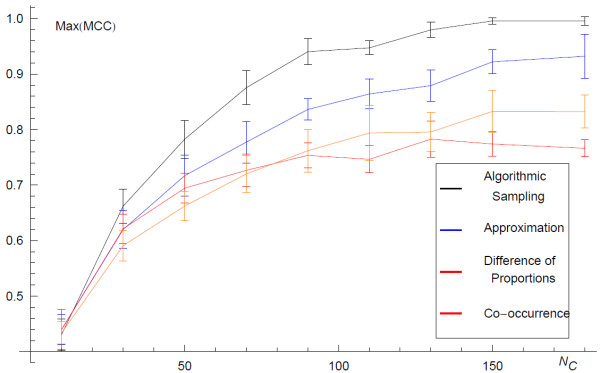
**Comparing algorithmic sampling to approximation and other methods.** The maximum value of the MCC over the full range of the threshold *p*_t_ plotted against the size of the field, *N*_C_. The error bars show the standard deviation over ten network inferences (each with 50 nodes). The four curves represent the resolution of the underlying network, *G*_*u*_, with increasing *N*_*C*_, when the network is inferred with the algorithmic sampling of *G*_*ens*_, the approximation shown in Equation 9, simple co-occurrence counting, and co-occurrence enrichment analysis using the chi-squared proportion test as described under alternative co-occurrence scoring methods.

**Figure 7:**
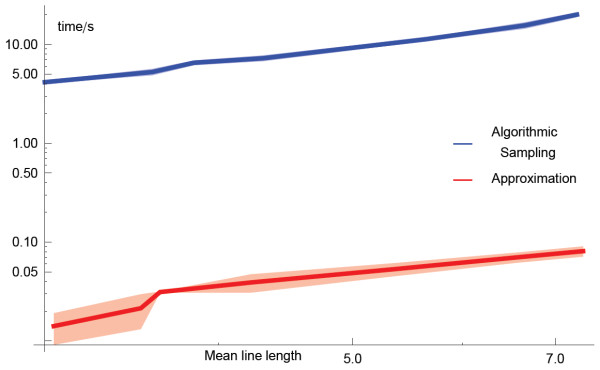
Dependence of the mean running time over 10 inference realizations for the inference of synthetic networks which have the parameters ***n* = 20**, ***N*_*c*_ = 100**, with GMT files generated with variable lengths of random walks.

### Running time and computational complexity

While the fully executed algorithm is potentially useful, in practice it is not practical due to its computational complexity. The operations to execute the fully enumerating algorithm depend on *N*_*e*_, *N*_*C*_, and the length of the elements in each *c*_*i*_, which are inherently random so we shall refer to the mean length *c*_*avg*_. From the structure of the pseudo-code we expect that the number of operations should increase in proportion to each of these three quantities, as well as the number of vertices n*, i.e.*, *O*(*N*_*e*_*N*_*C*_*c*_*avg*_). In the case of real data, *N*_*C*_ can be of the order of typically 10^3^ or 10^4^, and there can be typically thousands of vertices, so the number of operations for this method of inference can be prohibitively large. For the analytical approximation with Equation 9, described under Analytical Approximation above, the number of operations required for the computation depends on the details of the data as this determines the number of operations. The number of evaluations increases as *O*(*n*^2^). However, the number of operations required for each evaluation depends on the structure of the data within the GMT file. Comparison of running times between the fully executed algorithm and the approximation is shown in Figure 7Figure 7; this shows that the analytical approximation is two orders of magnitude faster than algorithmic sampling and therefore more practical for generating networks from real datasets stored as GMT files.

In the next sections we employ the approximation for the inference of PPIs from HT-IP/MS data, construct a network of stem cell regulators from ChIP-seq and loss-of-function/gain-of-function followed by expression data, construct a network between cancer drugs and severe side effects from patient records, as well as construct a co-authorship network connecting researchers from Mount Sinai School of Medicine in New York.

### Application to PPI prediction from HT-IP/MS data

The identification of binary interactions between proteins is an important task in systems biology. Initially, information on PPI networks in mammalian cells came from targeted experiments involving a small number of proteins. However, experimental techniques can now explore PPIs in human and mouse cells at large-scale. In addition, large numbers of PPIs from small-scale experimental studies are continually aggregated in publicly available databases.

In a recent study, Malovannaya et al. [8] reported the results from over 3000 HT-IP/MS experiments applied to nuclear co-regulators and members of their complexes in human cells. With this experimental technique, antibodies are used to pull-down a bundle of proteins that can be identified with mass-spectrometry. A total of 1796 antibodies were used in 3290 immuno-precipitation experiments of nuclear extracts from human cell-lines, in the course of which 315,215 individual protein detections were made. Non-specific binding events significantly contribute to the protein detection data and it is necessary to filter these out; we used the same filter used by the authors of the study [8] which reduced the number of individual protein detections to 100,341. Because the proteins are bound together in each pull-down, each of the 3290 experiments may be regarded as identifying a locally connected subgraph of the underlying binary PPI network. This type of information on the underlying network is equivalent to the class of data discussed above. The list of proteins identified in each experiment may be identified with the field *C*, and a mean adjacency matrix may be calculated from equation 9, then finally the sampling bias correction can be made to derive a predicted weighted adjacency matrix. This matrix quantifies the confidence of each binary direct physical PPI given solely the information on the connectivity of the underlying network derived from the collection of pull-down experiments. This approach is similar to our recently proposed reanalysis of this dataset since it does not consider the baits used in each experiment to determine direct protein interactions [7].

A large value of *p*_*i*,*j*_ may indicate that there is potentially sufficient information to suggest that protein *v*_*i*_ and *v*_*j*_ directly physically bind to each other. However, although large, the amount of data in this HT-IP/MS dataset is not large enough to fully resolve the underlying network of PPIs, so a small value of *p*_*i*,*j*_ does not necessarily indicate that the pair of proteins do not directly bind, only that there is not enough information in the dataset to suggest that they do. As the network is not fully resolved, the results of this inferential process could be used to rank the binary interactions to suggest likelihood of interactions for more targeted validation. Alternatively, the mean adjacency matrix could be used to gain a course grained global view of the human nuclear co-regulation complexome. To evaluate the reliability of predicted interactions we used benchmarking to compare predicted interactions to known interactions. Benchmarking is important for determining the quality and reliability of network inference approaches, and we attempt here to evaluate our inference approach applied to this data. The typical approach is to take the union of many current curated PPI databases and treat the interactions therein as true positives. This is imperfect because these databases contain significant numbers of false positives and false negatives, penalizing inferences that may be discovering correctly unknown interactions.

**Figure 8:**
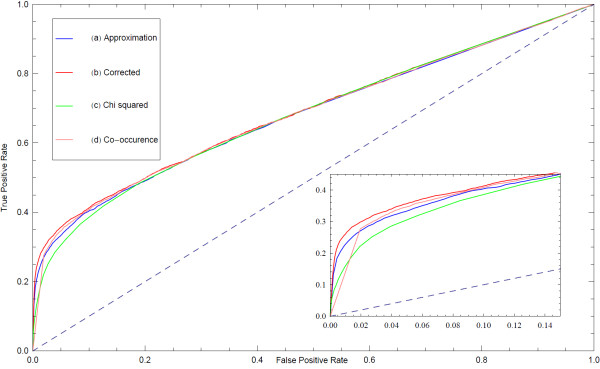
**Receiver operator characteristic (ROC) curves of the mean adjacency matrix of the ensemble *G***_***ens***_**created from the HT/IP-MS data inferred interactions created with the four different types of inference methods.** True positives are called based on known protein-protein interactions from published databases and publications used as the gold standard to evaluate the quality of the classification. Inset is a zoom-in of the most left portion of the ROC curve plots.

**Figure 9:**
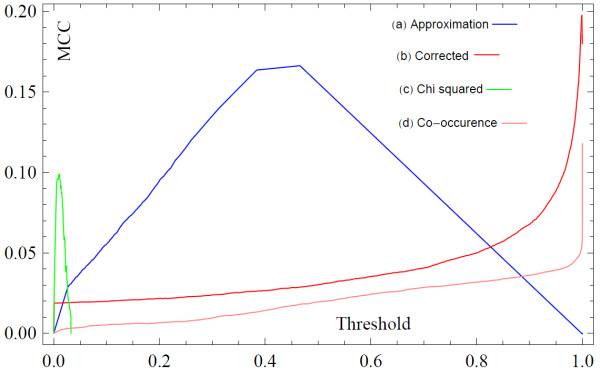
**The MMC as a function of the threshold value *p***_***t***_**applied to the mean adjacency matrix calculated for the HT/IP-MS data inferred interactions with the four different types of inference methods as compared to the PPI database to evaluate the quality of the classification.**

**Figure 10:**
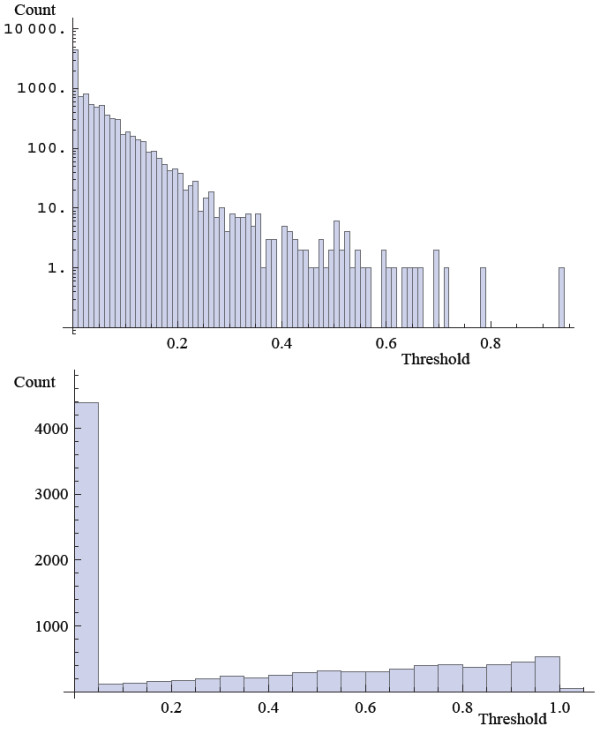
**Histograms of scores between all pair-wise proteins within the mean adjacency matrix of the ensemble *G***_***ens***_**created from the HT/IP-MS data inferred interactions created with the approximation algorithm before (top) and after (bottom) the bias adjustment.**

With these concerns in mind, we followed this procedure to evaluate our inference method. First we used the list of proteins identified in each pull-down to define the sets of proteins forming a connected subgraph of the underlying PPI network; this defines the subsets *c*_*i*_ composing the field *C*. We then used the approximation shown in equation 9 to estimate the mean adjacency matrix. We then collected PPI data from the following databases: BioGrid [14], HPRD [15], InnateDB [16], IntAct [17], KEGG [18], KEA [19], MINT [20], MIPS [21], DIP [22], BIND [23], BioCarta, PDZBase [24], PPID, Yu et al. [25], Stelzl et al. [26], Ewing et al. [27], Rual et al. [28] and Ma’ayan et al. [29]. We treated this data as the set of true positives and compared our mean adjacency matrix to it. To evaluate our ability to predict interactions we plotted the receiver operator characteristic curve (Figure 8) and the MCC as a function of the threshold value of *p*_*t*_ (Figure 9). In addition, we observe that prior to the sampling-bias-correction, the distribution of edge weights decays monotonically, whereas after, there is a bimodal distribution (Figure 10) which is reminiscent of the observed histogram shown in Figure 2 for the random network inference score distribution. The inhomogeneous distribution of protein frequencies throughout the experimental data suggests a sampling bias. However, the change in distribution of edge weights from unimodal to bimodal suggests that the bias has been removed to some extent. This is confirmed by the ROC and MCC curves that show that the bias adjustment results in improved accuracy above the uncorrected inference or co-occurrence computed using enrichment analysis with a Chi-squared test. Altogether, the predicted network of protein-protein interactions can suggest novel binary physical protein-protein interactions amenable for functional experimental validation. The top predicted interactions are provided in Additional file 1: Table S1 and on the web at http://www.maayanlab.net/S2N/PPI.html. 

**Figure 11:**
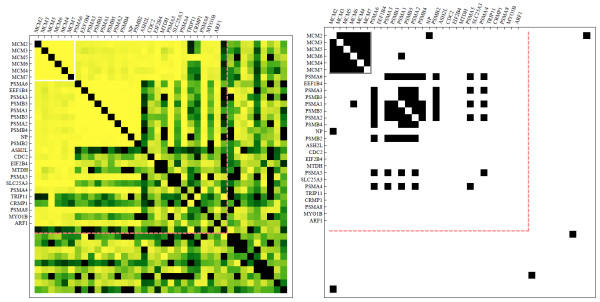
**Predicted interactions between members of the MCM complex as listed in the CORUM database and additional proteins that are predicted to strongly interact with the members of this complex.** On the left is a heatmap that visualizes the strength of the scores as predicted by the approximation method after applying the bias adjustment. On the right is a heatmap made of the same proteins where interactions are visualized as an adjacency matrix where black squares denotes known interactions in the PPI database we constructed from multiple published sources.

**Figure 12:**
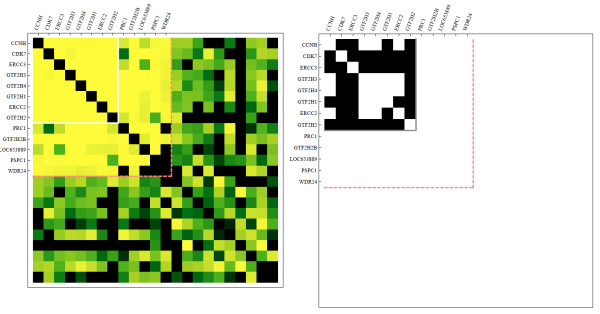
**Predicted interactions between members of the TFIIH transcription factor complex as listed in the CORUM database and additional proteins that are predicted to strongly interact with the members of this complex.** On the left is a heatmap that visualizes the strength of the scores as predicted by the approximation method after applying the bias adjustment. On the right is a heatmap made of the same proteins where interactions are visualized as an adjacency matrix where black squares denote known interactions in the PPI database we constructed from multiple published sources.

To further validate the inferred PPI network, we compared the ability of the predicted interactions to recover known protein complexes listed in the CORUM database [30]. We filtered the CORUM database, retaining only those complexes for which at least 80 % of the subunits are detected in the IP/MS data upon which we based our inference. In addition we retained only those CORUM complexes which had at least four proteins. The result was fifty CORUM protein complexes which could be potentially inferred from the IP/MS data. For each of these protein complexes, we generated three lists: 1) the known CORUM complex members; 2) proteins which are inferred to have significant interactions with the complex members either by having interaction-strength above the threshold resulting in maximum MCC with any individual member, or mean interaction strength across all complex member in the 95th percentile; 3) a list of random proteins to provide a scale for the background. We then performed hierarchical clustering on the complex members and a clustering based on clique membership on the additional proteins that strongly interact with the complex members. Such interactions are visualized in adjacency matrices heatmaps that show that our method recovered many known complexes and is capable of predicting additional members of known complexes (Figures 11 and 12, and Additional files 2 and 3). Adjacent to each heatmap of predicted interactions for each complex are connectivity maps in the same order where elements indicate membership in at least one of the PPI databases listed above. A comparison of these two adjacency matrices shows that the CORUM complexes are clearly well inferred with our method, and also that we can observe interactions with potential proteins and protein complexes which are currently undiscovered experimentally. Focusing on two such complexes, Figure 11 shows identified relationship between the mini chromosome maintenance (MCM) complex and the proteasome, whereas in Figure 12 additional components are suggested for the TFIIH transcription factor complex. The relationship between the MCM complex and the proteasome appears to be divided into two mutually exclusive sets of proteasome related components that do not interact with each other. 

### Revealing associations between stem cell regulators

**Figure 13:**
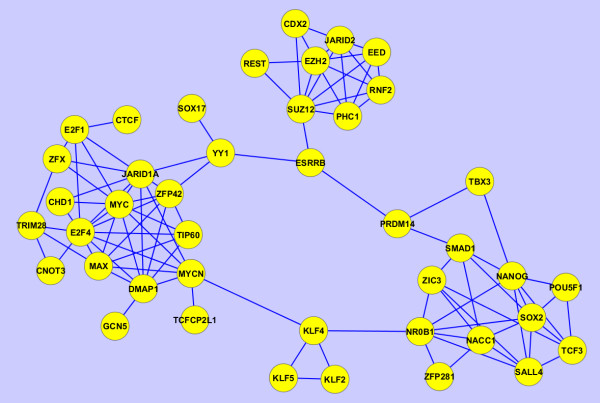
Subnetwork of inferred interactions between pluripotency and self-renewal regulators as determined by applying the approximation method with the Bias Adjustment on the ChIP-seq dataset of profiling these factors and regulators in mouse embryonic stem cells (mESCs).

**Figure 14:**
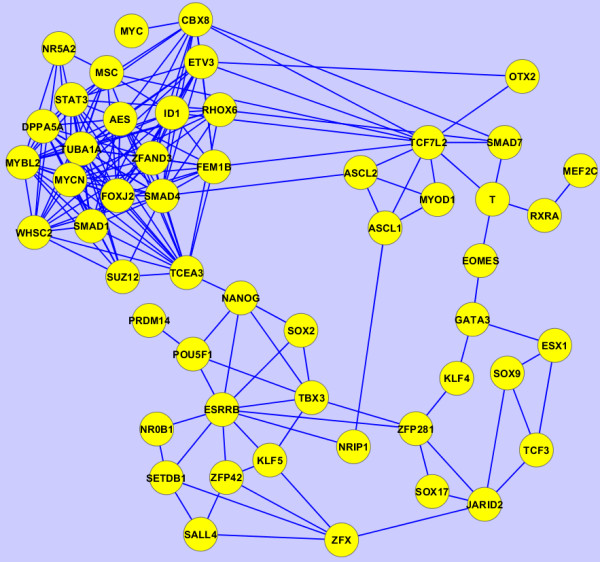
Subnetwork of inferred interactions between pluripotency and self-renewal regulators as determined by applying the approximation method with the bias adjustment to the LOF/GOF followed by gene expression microarrays dataset when perturbing these factors and regulators in mESCs.

**Figure 15:**
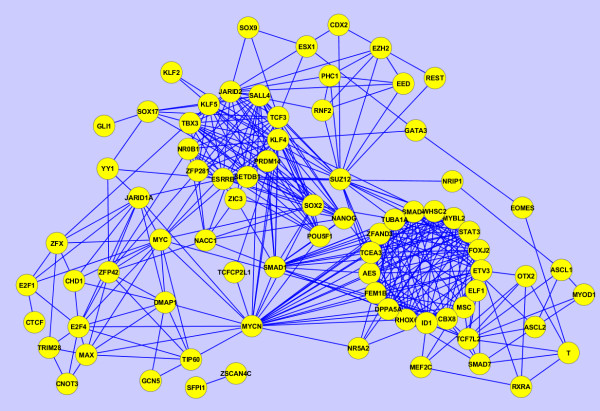
**Subnetwork of inferred interactions between pluripotency and self-renewal regulators as determined by applying the approximation method with the bias adjustment on the ChIP-seq dataset of profiling these factors and regulators in mESCs as well as the LOF/GOF followed by gene expression microarrays dataset when perturbing these factors and regulators in mESCs.** The scores were combined using Equation 10.

In the past few years a tremendous number of high-content experiments have profiled different aspects of mouse embryonic stem cells. Such experiments include gene-expression microarrays at different conditions, genome-wide histone modification and transcription factor binding to DNA using ChIP-seq, RNAi screens for identifying pluripotency regulators, proteomics, phosphoproteomics and microRNA profiling. While these experiments have the potential to fully uncover the regulatory networks governing stem-cell maintenance and differentiation into specific lineages, data integration across regulatory layers to extract new knowledge from such data is challenging [31]. The network inference method we employ here can be utilized for data integration by building networks from various sources of data. Using Equation 10 we can construct networks that combine information from multiple types of GMT files. From the stem cell literature we collected publications that reported: a) transcription factor, and other transcriptional regulators, binding to the promoters of target genes as determined by ChIP-seq or ChIP-chip experiments; and b) gene expression changes after the knockdown (loss-of-function) or over-expression (gain-of-function) of these factors and regulators as determined by gene expression arrays. We then converted such data into two GMT files: the first with each row having a set of transcription factor target genes from each ChIP-seq or ChIP-chip experiment, and the second having in each row a set of differentially expressed genes for each factor or regulator from each gene expression loss-of-function (LOF) or gain-of-function (GOF) experiment. We then transposed these two files were the target genes are the row labels and the factors and regulators are listed in each row. Finally, we generated three networks: 1) based on the ChIP data alone (Figure 13); 2) based on the gene-expression after knockdown or over-expression data (Figure 14); and 3) based on combined data using Equation 10 (Figure 15). The ChIP network contains four clusters, the first of which includes the known pluripotency maintenance regulators: Nanog, Oct4 and Sox2; the second cluster corresponds to the polycomb repressive complex (PRC); the third cluster includes early differentiation regulators including Myc and MycN; the remaining cluster is made of Klf4, Klf2 and Klf5 which likely have overlapping unique set of target genes (Figure 13). The LOF/GOF followed by expression network is more difficult to explain. Nanog, Oct4 and Sox2 still appear together and are directly connected. However, the other components appear in other clusters that are more difficult to define based on what is known (Figure 14). Finally, the combined network shows an interesting pattern: the triad, Nanog, Oct4 and Sox2 appears in the center of two highly connected clusters, one containing other known pluripotency regulators including Tbx3, Esrrb, Klf4, Sall4 and Tcf3 whereas the other cluster contains more differentiation components. It is plausible that the triad of Nanog, Oct4 and Sox2 is regulating both highly dense circuits keeping the appropriate balance between early differentiation and self-renewal maintenance states. 

**Figure 16:**
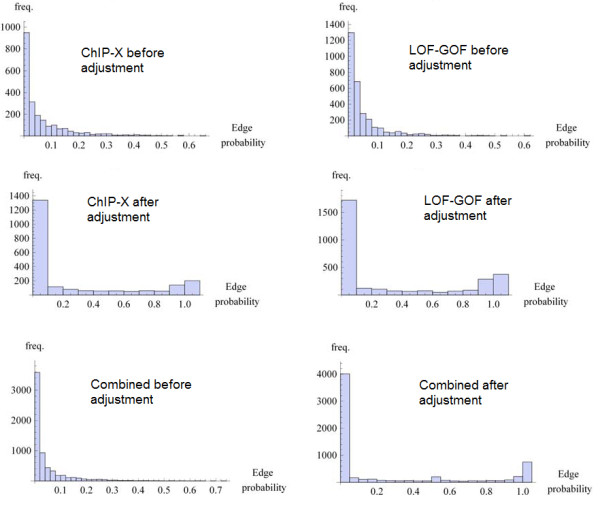
**Histograms of scores between all pair-wise transcription factors and regulators within the mean adjacency matrices of the ensembles *G***_***ens***_**created for generating the three networks visualized in Figures **13 **, **14 **and **15 **before (left) and after (right) the bias adjustment.**

It is important to point out that the GMT files, both from the ChIP data and LOF/GOF gene expression data have large *N*_*c*_, i.e., many rows in each of the two GMT files. In addition, each row is relatively short, having only few factors or regulator listed in each row. As seen from the examples applied on random artificial networks, such data should recover networks with high fidelity because it is likely to contain enough information to recover the network. Indeed, histograms of the scores show clear segregation of scores into high and low after applying the bias adjustment (Figure 16). However, the networks that govern stem cell regulation are mostly unknown so we cannot fully validate these inferred networks.

### Identifying statistical interactions between drugs and side effects

**Figure 17:**
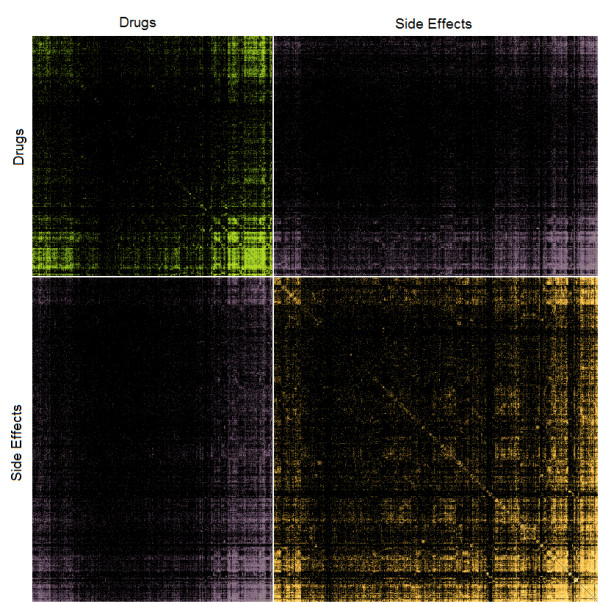
**Statistical interactions between drugs (green), side-effects (purple) and drugs/side-effects (orange) created from patient records (rows) from the AERS database and applying the network inference method on the most recent 50,000 entries.** Color intensity represents the strength of the link. Drugs and side-effects are hierarchically clustered separately.

**Figure 18:**
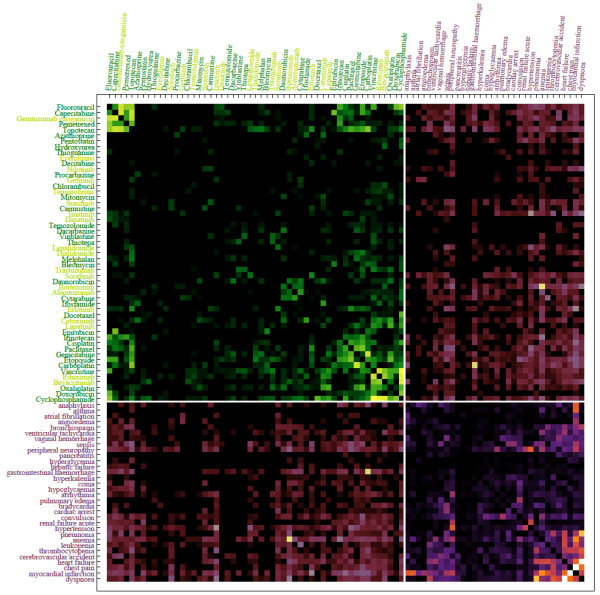
**Heatmap of an adjacency matrix connecting 53 cancer drugs and 32 severe side-effects.** Statistical interactions between cancer drugs: light green (n = 19) are drugs that target cell signalling components, and dark green (n = 34) are cytotoxic drugs; as well as 32 severe side-effects (purple) and drugs/side-effects interactions (orange) created from patient records (rows) from the AERS database applying the network inference method on the most recent 50,000 entries. Color intensity represents the strength of the link. Drugs and side-effects are hierarchically clustered separately.

Next, we used the network inference approach to mine statistical interactions from the FDA’s spontaneous adverse event reporting system (AERS). This database contains millions of records entered by physicians in the United States recording data from patients. Each record contains a patient record number, the drugs the patient was taking and the adverse events they experienced. From the database we first extracted the most recent one million records (April, 2012). To consolidate drug names we converted all entered drug names to their generic names using synonyms from DrugBank [32] and drugs.com. Records that contained drug names that could not be mapped to generic drug names were dropped. This resulted in a loss of about ~20 % of all records. We then treated the left-over 793,789 entries from the AERS database as the potential set *c*_*i*_ where we treated drugs and side-effects as connected subgraphs in the underlying drug-drug, drug/side-effect, and side-effect/side-effect statistical interaction network. Because of the computational complexity of the problem and to save execution time, we only used the most recent 50,000 records from this dataset to create the actual network we visualize (Figure 17). The meaning of drug-drug interactions in this context is that these drugs are commonly co-prescribed; side-effect/side-effect interactions mean that the side-effects co-occur; and drug/side-effect interactions are common associations between drugs and side-effects. In total, the resultant network connected 726 drugs and 980 side effects. The visualization of this network as a heatmap adjacency matrix clearly shows that there are clusters of co-prescribed drugs and co-occurring side effects and interactions between these clusters (Figure 17). To drill down into more specific examples of such interactions we selected a subset made of 53 drugs used to treat cancer: 34 cytotoxic drugs and 19 drugs that target cell signaling pathways. We connected these drugs to a subset made of 32 severe side effects (Figure 18). This heatmap shows a strong link between chemotherapeutics and cardiovascular related adverse events where cytotoxic cancer therapeutics are commonly co-prescribed with drugs that target cell signaling components. One of the interesting links that the method uncovered is the strong link between Anemia and Bortezomib, a cancer drug that is also used to treat other diseases such as lupus. Whether Bortezomib is helpful in curbing anemia, causes anemia, or should be prescribed to patients that already have anemia is controversial [33]. It also highlights the fact that side-effect entries in AERS are not necessarily indicative of a direct causative relationship, and apparent statistical relations between taking drugs and side effects can appear due to other correlates, and could even appear when the drugs is prescribed to treat the side-effect. While this network of drugs and side-effects illuminates some initial relationships between approved drugs and the side-effects these drugs elicit, there is much more to explore in this realm. Causative relationships and correlation artifacts may leave their imprint in the network structure as revealed with our method. For example, the innocent bystander effect may appear as a clique where a group of drugs have a strong connection to a side-effect, but also a strong connection to each other. This then opens up the possibility of applying additional network analysis to extract causative drug/side-effect relationships from our initial undirected drug/side-effect network. 

### Mount Sinai collaboration network

**Figure 19:**
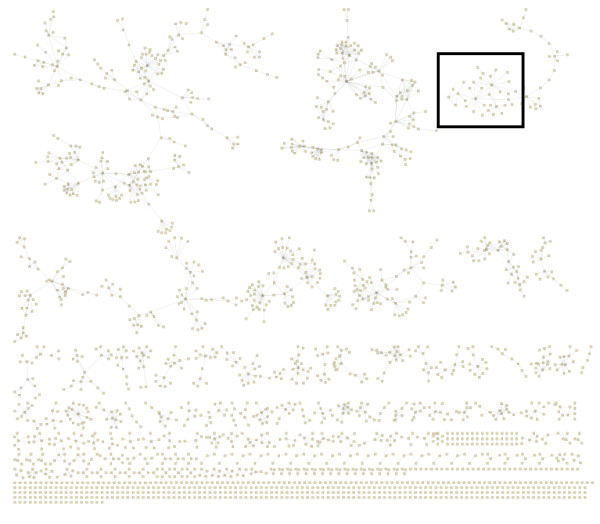
Collaboration network created by considering publications as the rows in a GMT file for publications from Mount Sinai School of Medicine investigators downloaded from PubMed.

**Figure 20:**
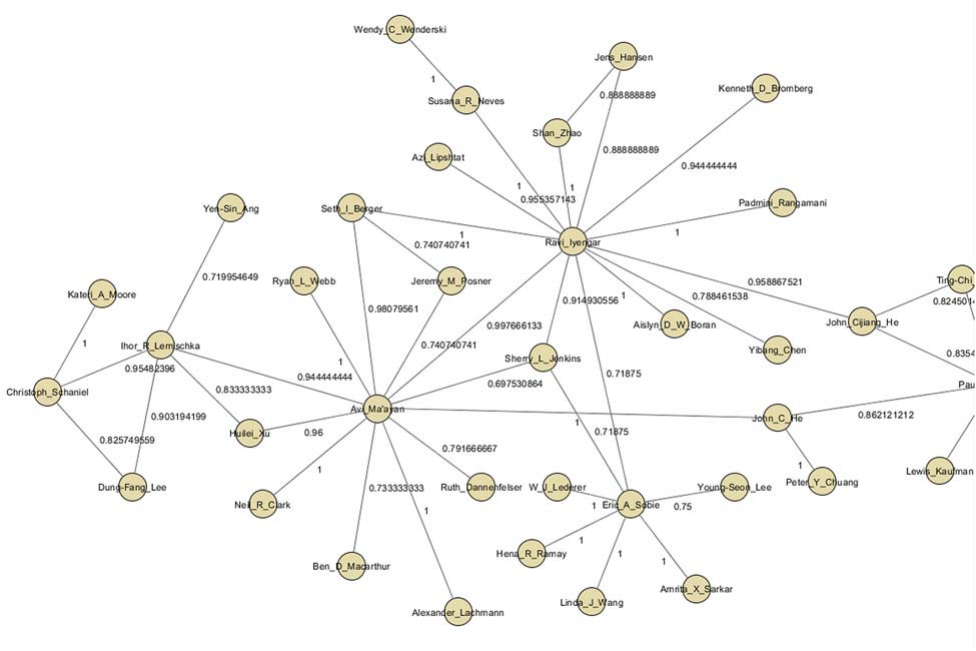
**Zooming into the highlighted subnetwork in Figure**19**to show relationships between authors and the scores computed to connect them.**

Finally, we show how the network inference approach can be applied more broadly to construct other types of networks. The GMT representation lends naturally to the inference of co-authorship networks where each row in the GMT file derives from a publication where the authors of the publication are listed in each row. Using PubMed E-utilities’ E-search function we searched for the latest (early May 2012) publications that contain an affiliation equal to the term Mount Sinai School of Medicine. From the returned list of publications, we downloaded the top 5,000 abstracts returned by the search query and extracted the author list using the E-fetch function. For each paper, the data was formatted into a GMT file with the PubMed ID as the set label and each author of each paper as the members of each set. After the assembly of the GMT file, the approximation algorithm was applied to the data. The final network contains only edges with scores higher than 0.67 (Figure 19, http://www.maayanlab.net/S2N/mssm.html). The network shows known clusters of investigators from various departments identifying many of the relationships we are familiar with and can confirm (Figure 20). 

## Conclusions

Network inference is a process by which a network is resolved from indirect data. In cases where direct determination of the network is difficult or impossible it is necessary to use indirect evidence which can be more easily obtained. Here we show that using collections of related entities, which are easy to accumulate, we can resolve the underlying network. We drew the analogy that the indirect empirical data describes a macrostate, and the ensemble of networks consistent with this data is the available microstates. As the empirical data accrues there are more constraints and the size of the microstate ensemble shrinks until the underlying network resolves. We employed the formulation of ERGMs from Park and Newman [11], providing a statistical mechanical framework for artificial network models, to derive the appropriate ensemble. The network ensemble was then used to evaluate the statistical evidence for the underlying network given the data. We applied the approach to synthetic data we generated as well as to two pertinent specific examples from systems biology, namely PPI prediction and stem-cell regulatory network reconstruction. In addition we provide one example from systems pharmacology, namely identifying statistical interactions between drugs and side-effects, and one example of co-authorship network, connecting Mount Sinai investigators. The networks we reconstructed are not only validated by known interactions but also offer predictions for interactions that are likely real and could be further validated experimentally. All these networks and datasets are provided on the web at http://www.maayanlab.net/S2N with a web-based open source software tool that can be used to convert any entity-set library to a network. While useful for addressing problems in current biology and biomedicine, the approach is of general significance and can be applied in other fields that study complex systems.

## Competing interests

The author(s) declare that they have no competing interests.

## Authors’ contributions

NRC and AM designed the study and wrote the manuscript. NRC generated all figures and conducted the computational analyses and mathematical derivations. RD developed the web-site. MEK collected PPI interactions from publicly available resources. CMT processed the data from AERS. All authors read and approved the final manuscript.

## Supplementary Material

Additional file 1 Predicted PPIs. Click here for file

Additional file 2 List of 50 complexes. Click here for file

Additional file 3 Images of predicted 50 complexes. Click here for file
