# Supplementary material for: Sets2Networks: network inference from repeated observations of sets
Source: BMC Syst Biol. 2012 Jul 23;6:89. doi: 10.1186/1752-0509-6-89 (PMC3443648; doi:10.1186/1752-0509-6-89)
Supplement: Additional file 3 — Images of predicted 50 complexes. [file 1752-0509-6-89-S3.pdf]

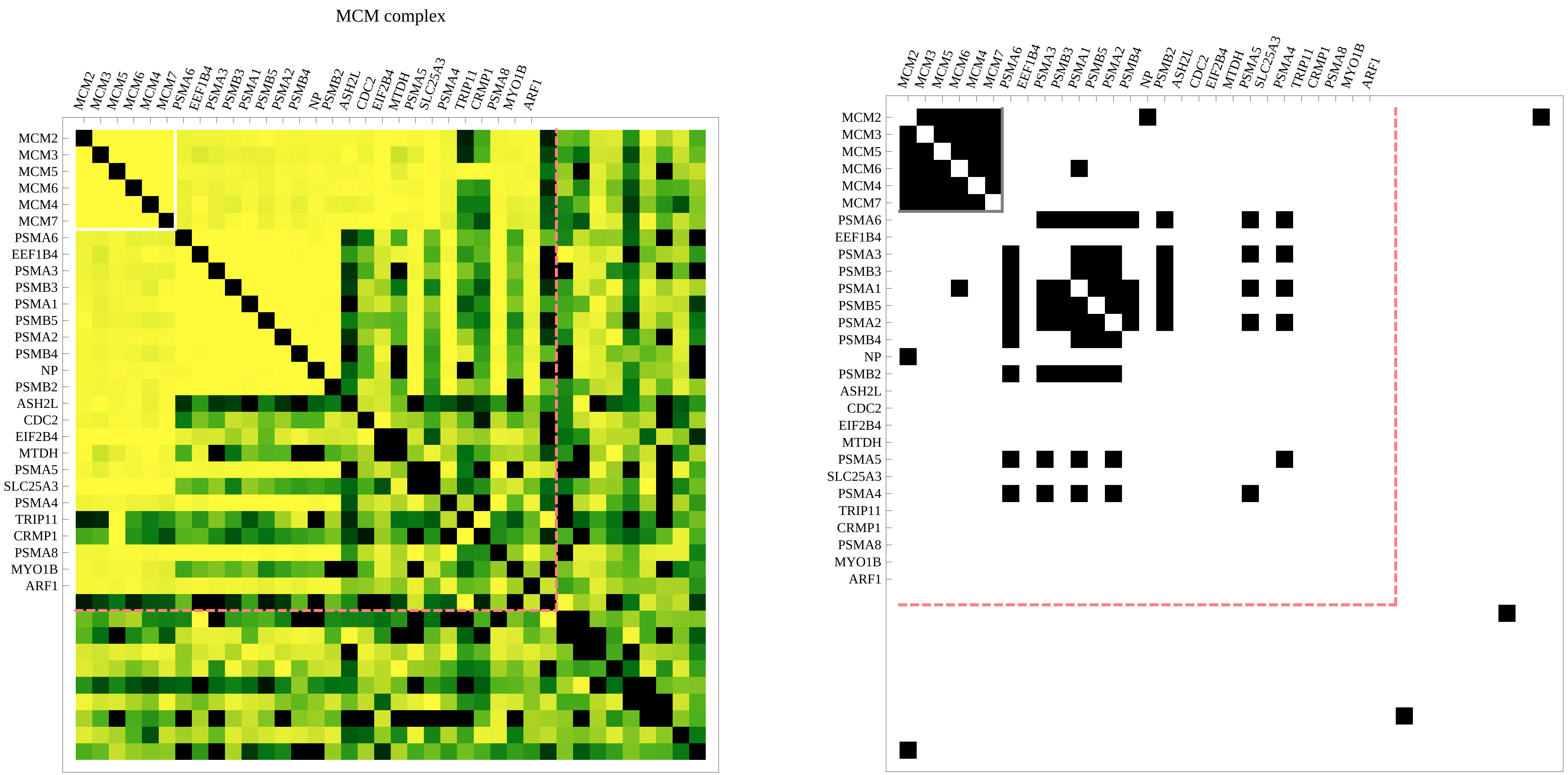

MCM complex
MCM2
MCM3
MCM5
MCM6
MCM4
MCM7
PSMA6
EEF1B4
PSMA3
PSMB3
PSMA1
PSMB5
PSMA2
PSMB4
NP
PSMB2
ASH2L
CDC2
EIF2B4
MTDH
PSMA5
SLC25A3
PSMA4
TRIP11
CRMP1
PSMA8
MYO1B
ARF1

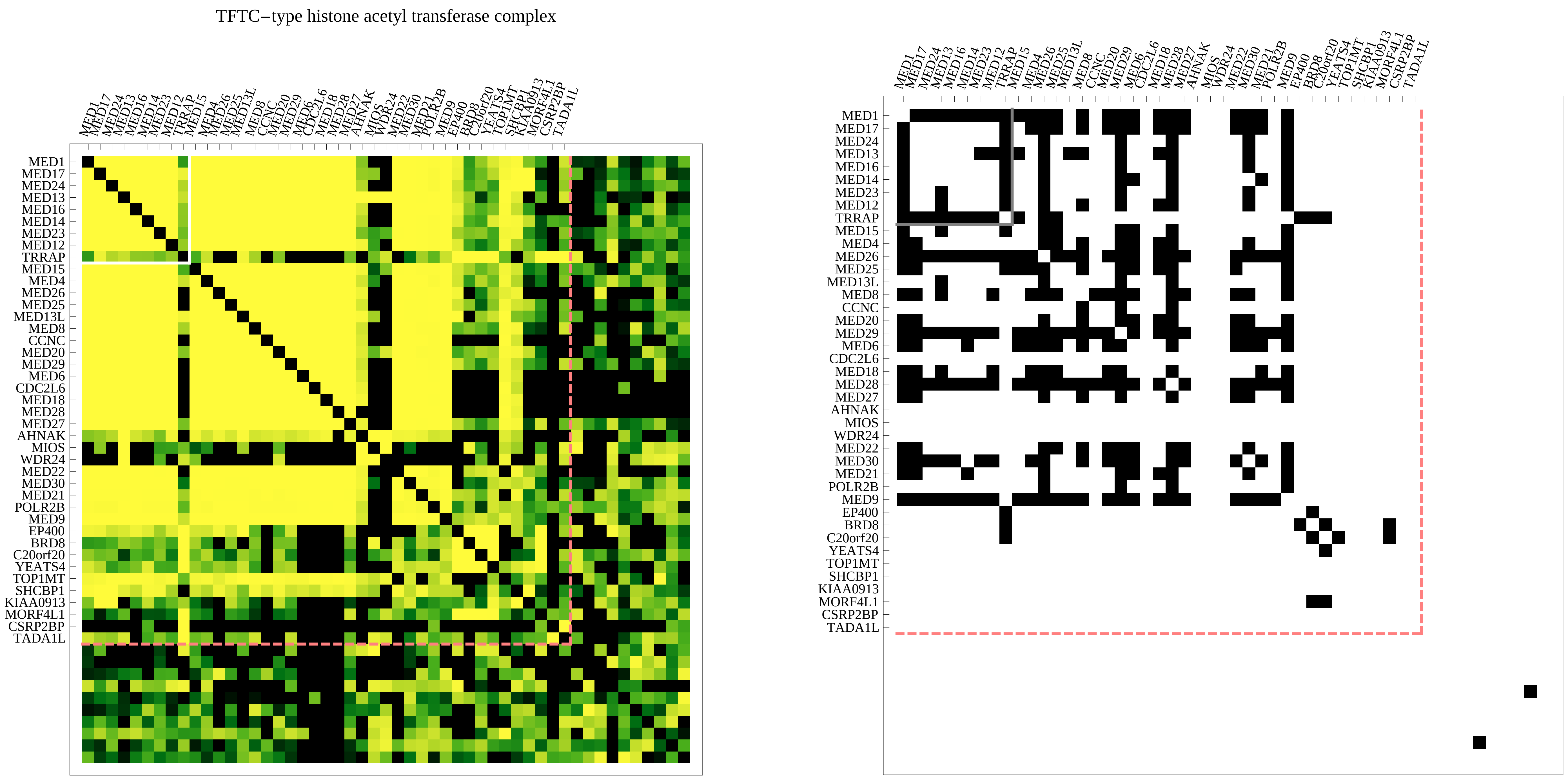

TFTC−type histone acetyl transferase complex
MED1
MED17
MED24
MED13
MED16
MED14
MED23
MED12
TRRAP
MED15
MED4
MED26
MED25
MED13L
MED8
CCNC
MED20
MED29
MED6
CDC2L6
MED18
MED28
MED27
AHNAK
MIOS
WDR24
MED22
MED30
MED21
POLR2B
MED9
EP400
BRD8
C20orf20
YEATS4
TOP1MT
SHCBP1
KIAA0913
MORF4L1
CSRP2BP
TADA1L

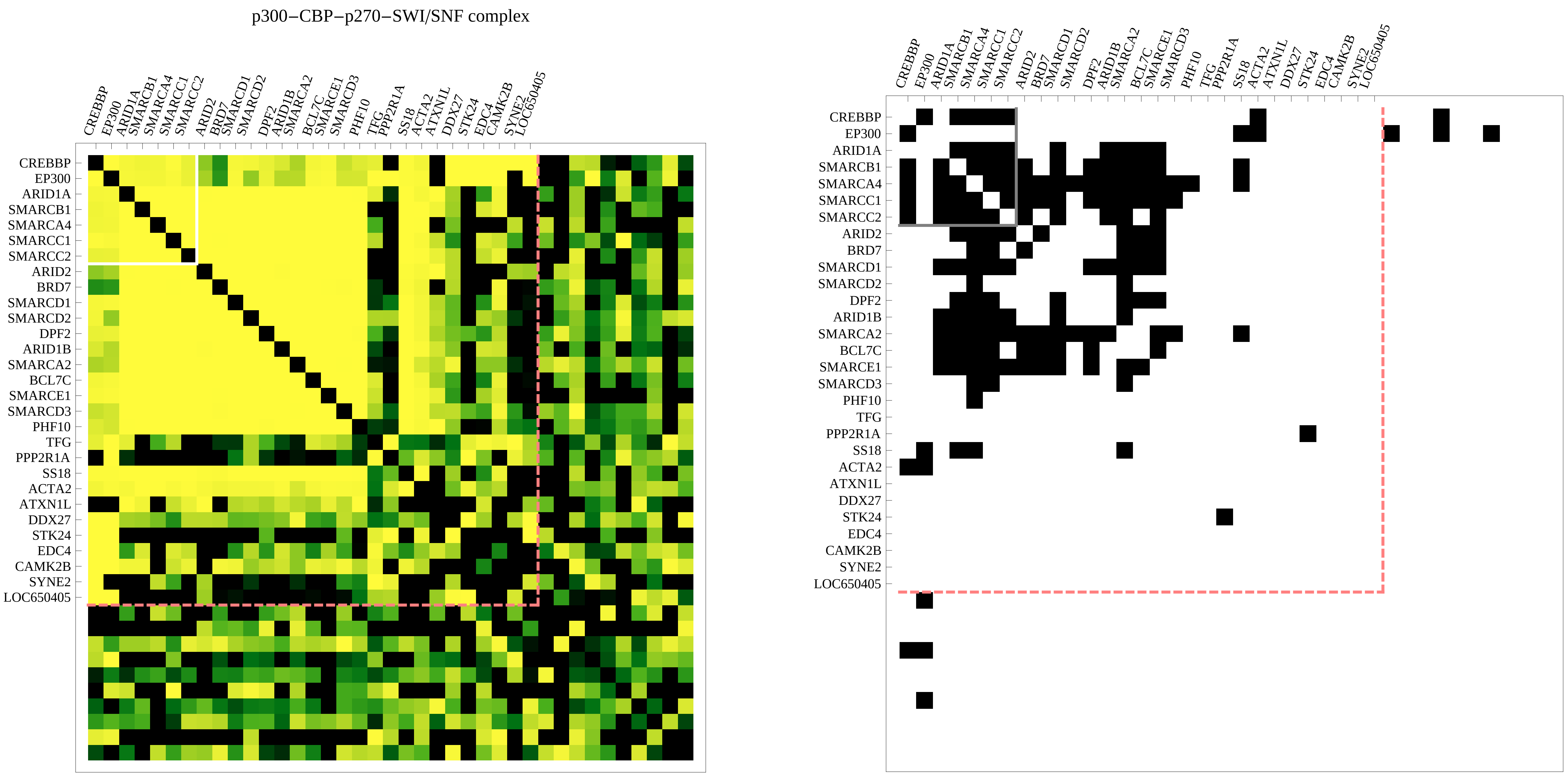
p300−CBP−p270−SWI/SNF complex
CREBBP
EP300
ARID1A
SMARCB1
SMARCA4
SMARCC1
SMARCC2
ARID2
BRD7
SMARCD1
SMARCD2
DPF2
ARID1B
SMARCA2
BCL7C
SMARCE1
SMARCD3
PHF10
TFG
PPP2R1A
SS18
ACTA2
ATXN1L
DDX27
STK24
EDC4
CAMK2B
SYNE2
LOC650405

NRD complex (Nucleosome remodeling and deacetylation complex)

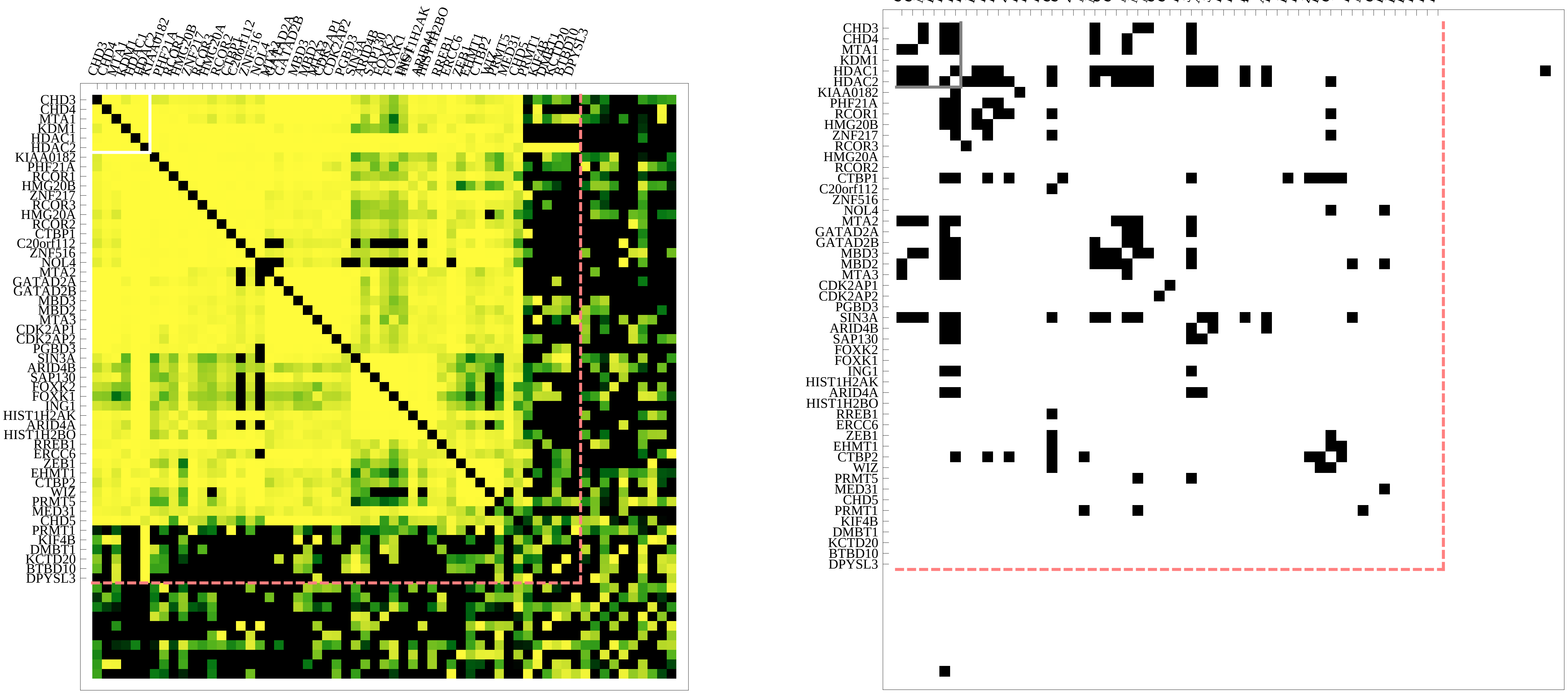

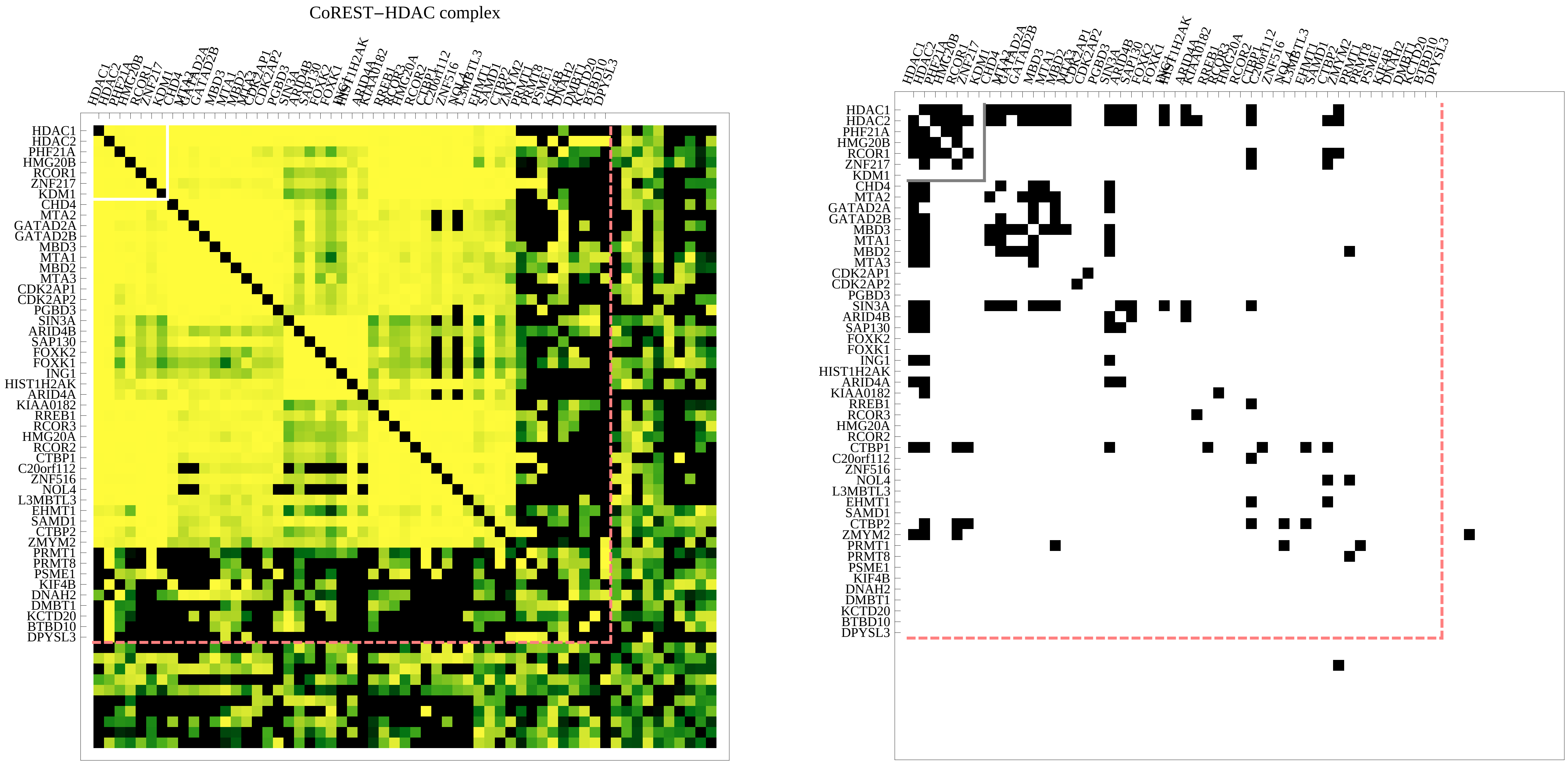
CoREST−HDAC complex
HDAC1
HDAC2
PHF21A
HMG20B
RCOR1
ZNF217
KDM1
CHD4
MTA2
GATAD2A
GATAD2B
MBD3
MTA1
MBD2
MTA3
CDK2AP1
CDK2AP2
PGBD3
SIN3A
ARID4B
SAP130
FOXK2
FOXK1
ING1
HIST1H2AK
ARID4A
KIAA0182
RREB1
RCOR3
HMG20A
RCOR2
CTBP1
C20orf112
ZNF516
NOL4
L3MBTL3
EHMT1
SAMD1
CTBP2
ZMYM2
PRMT1
PRMT8
PSME1
KIF4B
DNAH2
DMBT1
KCTD20
BTBD10
DPYSL3

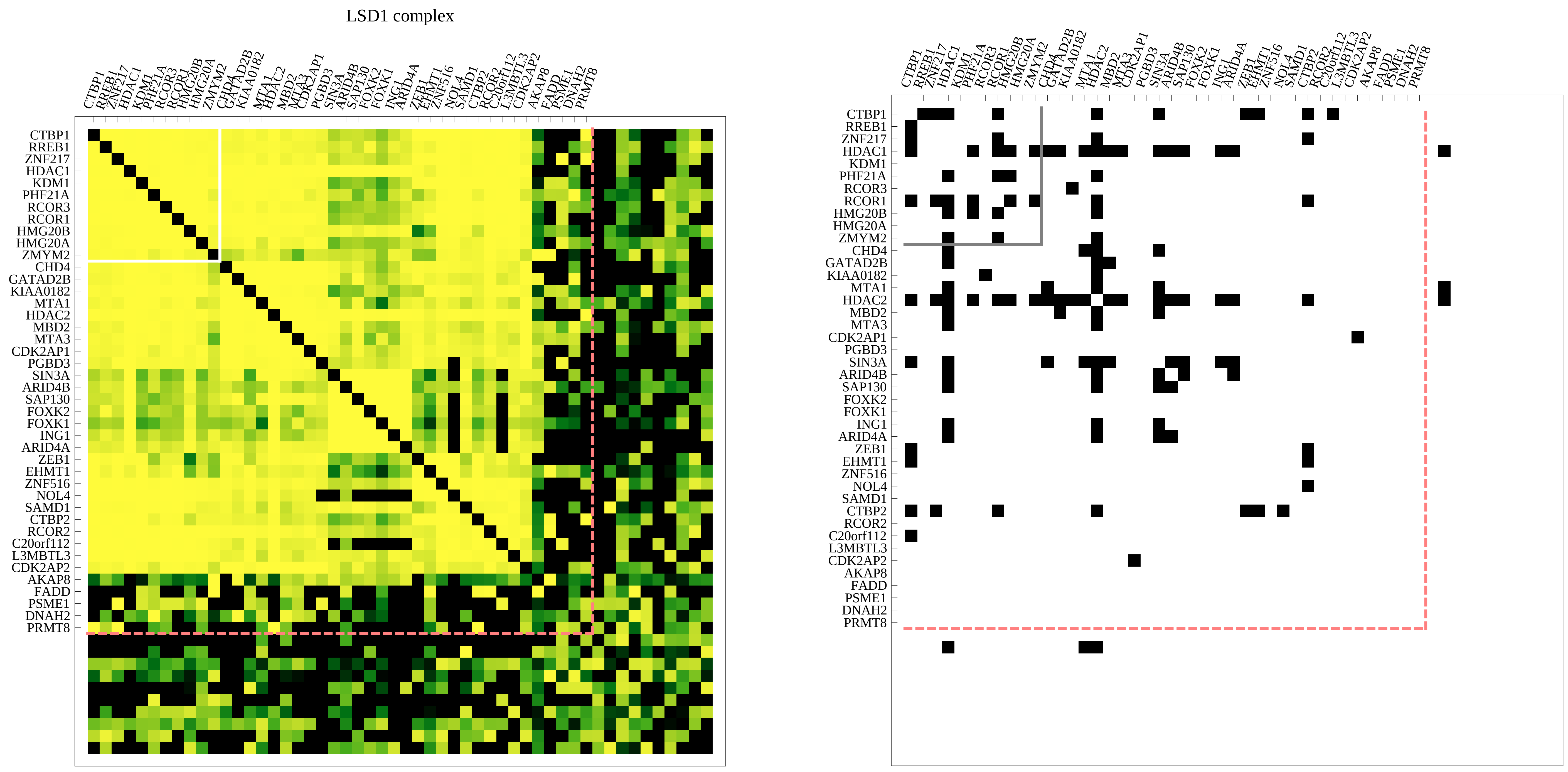
LSD1 complex
CTBP1
RREB1
ZNF217
HDAC1
KDM1
PHF21A
RCOR3
RCOR1
HMG20B
HMG20A
ZMYM2
CHD4
GATAD2B
KIAA0182
MTA1
HDAC2
MBD2
MTA3
CDK2AP1
PGBD3
SIN3A
ARID4B
SAP130
FOXK2
FOXK1
ING1
ARID4A
ZEB1
EHMT1
ZNF516
NOL4
SAMD1
CTBP2
RCOR2
C20orf112
L3MBTL3
CDK2AP2
AKAP8
FADD
PSME1
DNAH2
PRMT8

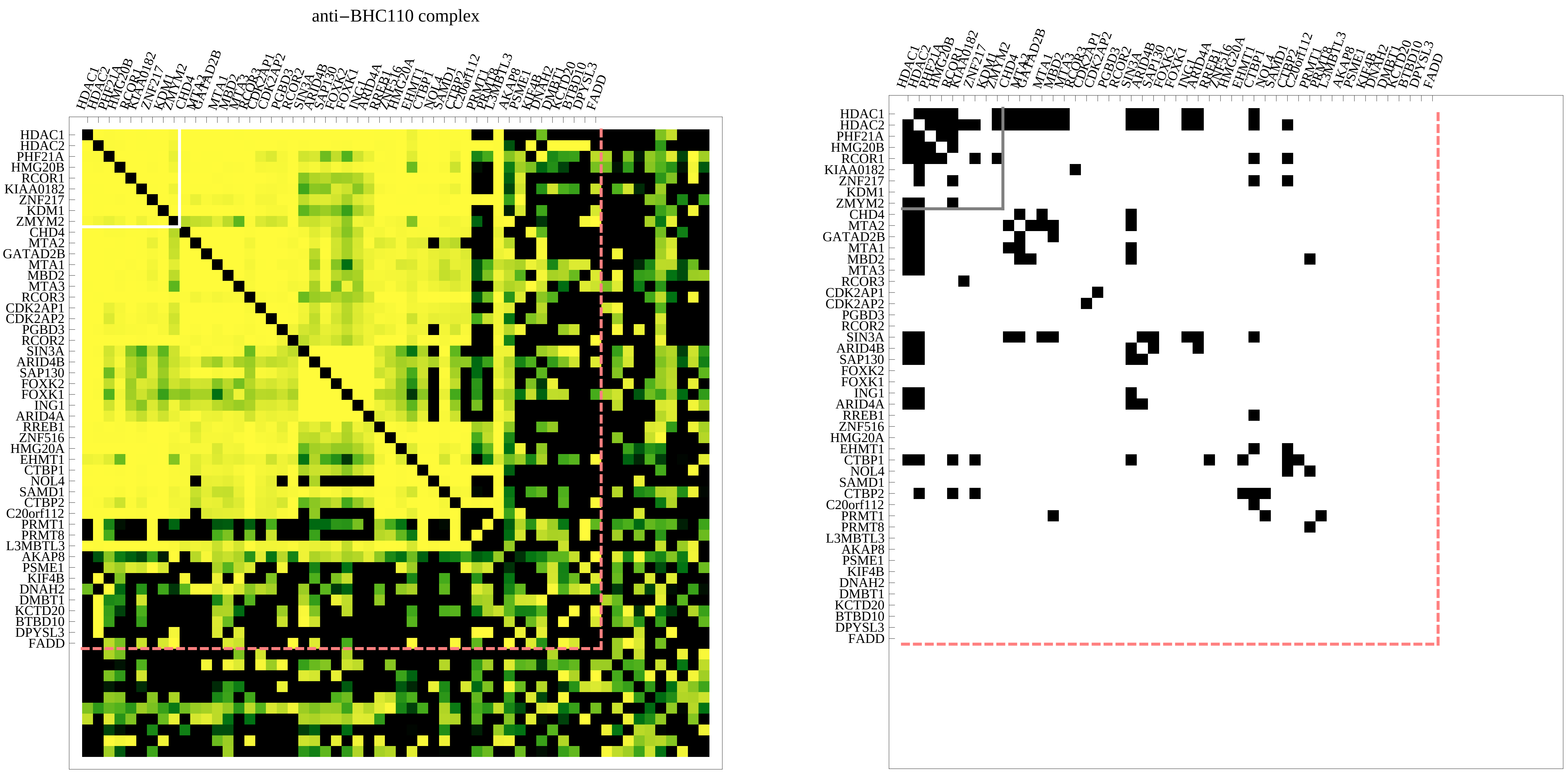
anti−BHC110 complex
HDAC1
HDAC2
PHF21A
HMG20B
RCOR1
KIAA0182
ZNF217
KDM1
ZMYM2
CHD4
MTA2
GATAD2B
MTA1
MBD2
MTA3
RCOR3
CDK2AP1
CDK2AP2
PGBD3
RCOR2
SIN3A
ARID4B
SAP130
FOXK2
FOXK1
ING1
ARID4A
RREB1
ZNF516
HMG20A
EHMT1
CTBP1
NOL4
SAMD1
CTBP2
C20orf112
PRMT1
PRMT8
L3MBTL3
AKAP8
PSME1
KIF4B
DNAH2
DMBT1
KCTD20
BTBD10
DPYSL3
FADD

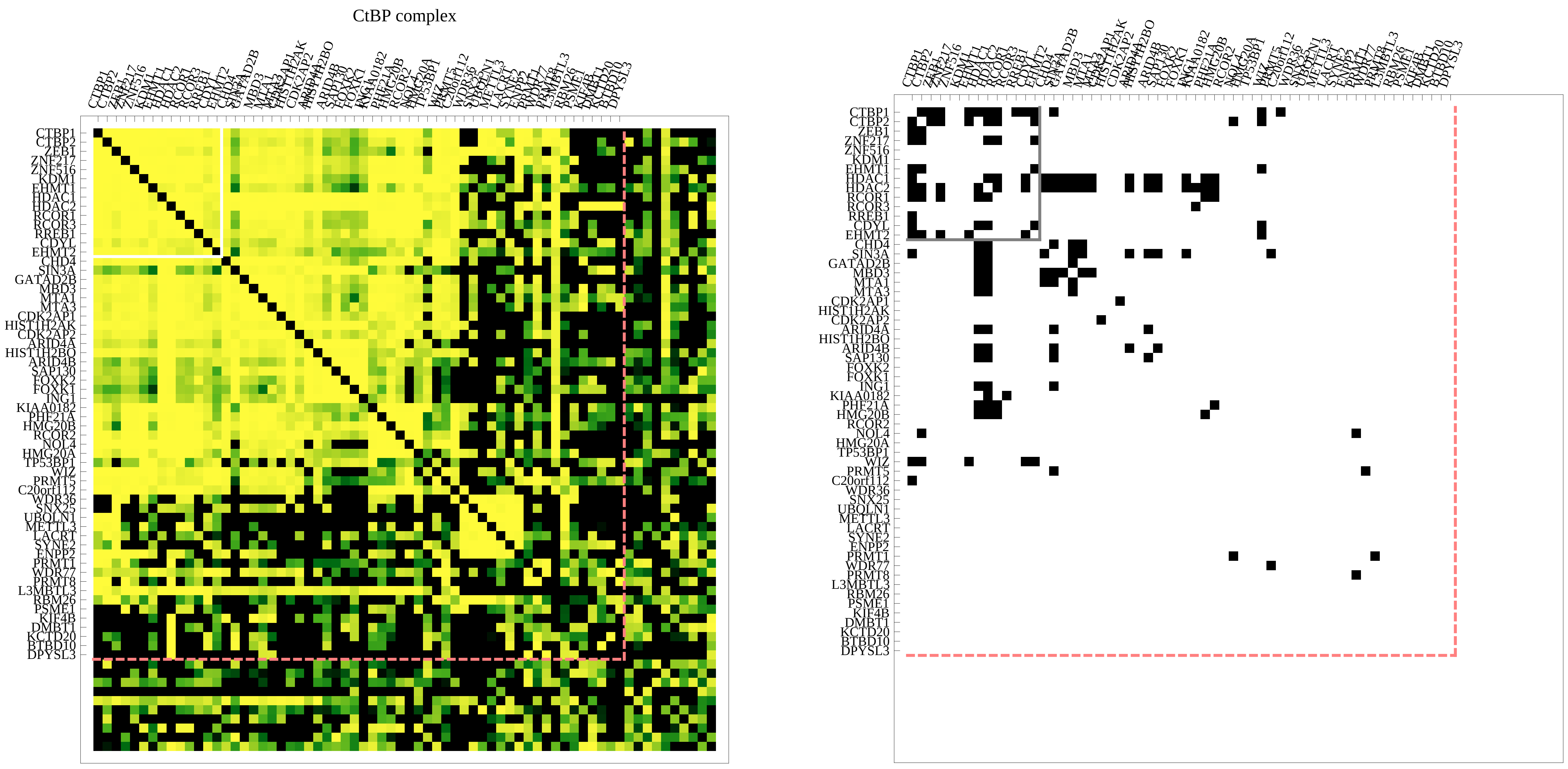
CtBP complex
CTBP1
CTBP2
ZEB1
ZNF217
ZNF516
KDM1
EHMT1
HDAC1
HDAC2
RCOR1
RCOR3
RREB1
CDYL
EHMT2
CHD4
SIN3A
GATAD2B
MBD3
MTA1
MTA3
CDK2AP1
HIST1H2AK
CDK2AP2
ARID4A
HIST1H2BO
ARID4B
SAP130
FOXK2
FOXK1
ING1
KIAA0182
PHF21A
HMG20B
RCOR2
NOL4
HMG20A
TP53BP1
WIZ
PRMT5
C20orf112
WDR36
SNX25
UBQLN1
METTL3
LACRT
SYNE2
ENPP2
PRMT1
WDR77
PRMT8
L3MBTL3
RBM26
PSME1
KIF4B
DMBT1
KCTD20
BTBD10
DPYSL3

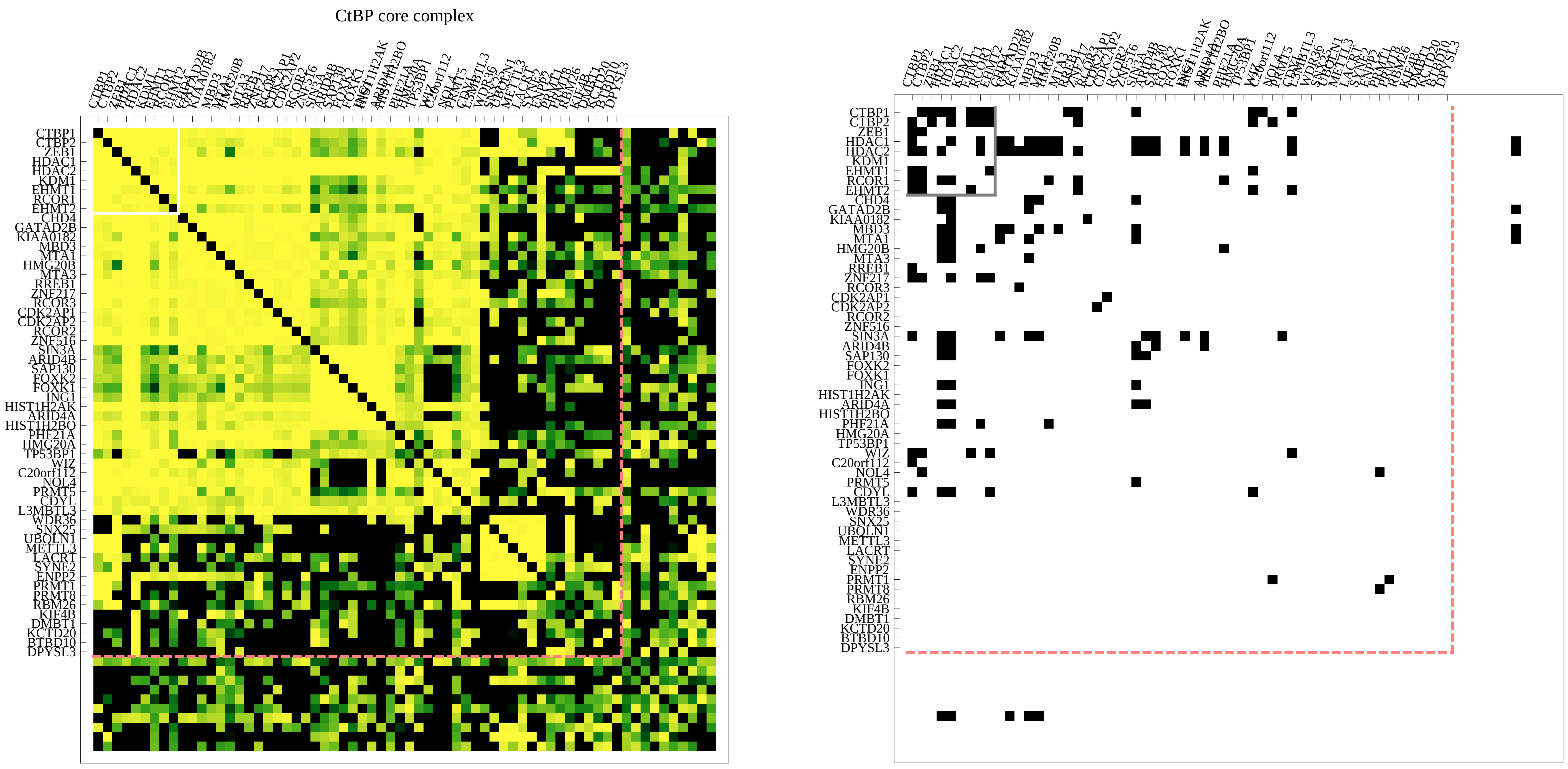
CtBP core complex
CTBP1
CTBP2
ZEB1
HDAC1
HDAC2
KDM1
EHMT1
RCOR1
EHMT2
CHD4
GATAD2B
KIAA0182
MBD3
MTA1
HMG20B
MTA3
RREB1
ZNF217
RCOR3
CDK2AP1
CDK2AP2
RCOR2
ZNF516
SIN3A
ARID4B
SAP130
FOXK2
FOXK1
ING1
HIST1H2AK
ARID4A
HIST1H2BO
PHF21A
HMG20A
TP53BP1
WIZ
C20orf112
NOL4
PRMT5
CDYL
L3MBTL3
WDR36
SNX25
UBQLN1
METTL3
LACRT
SYNE2
ENPP2
PRMT1
PRMT8
RBM26
KIF4B
DMBT1
KCTD20
BTBD10
DPYSL3

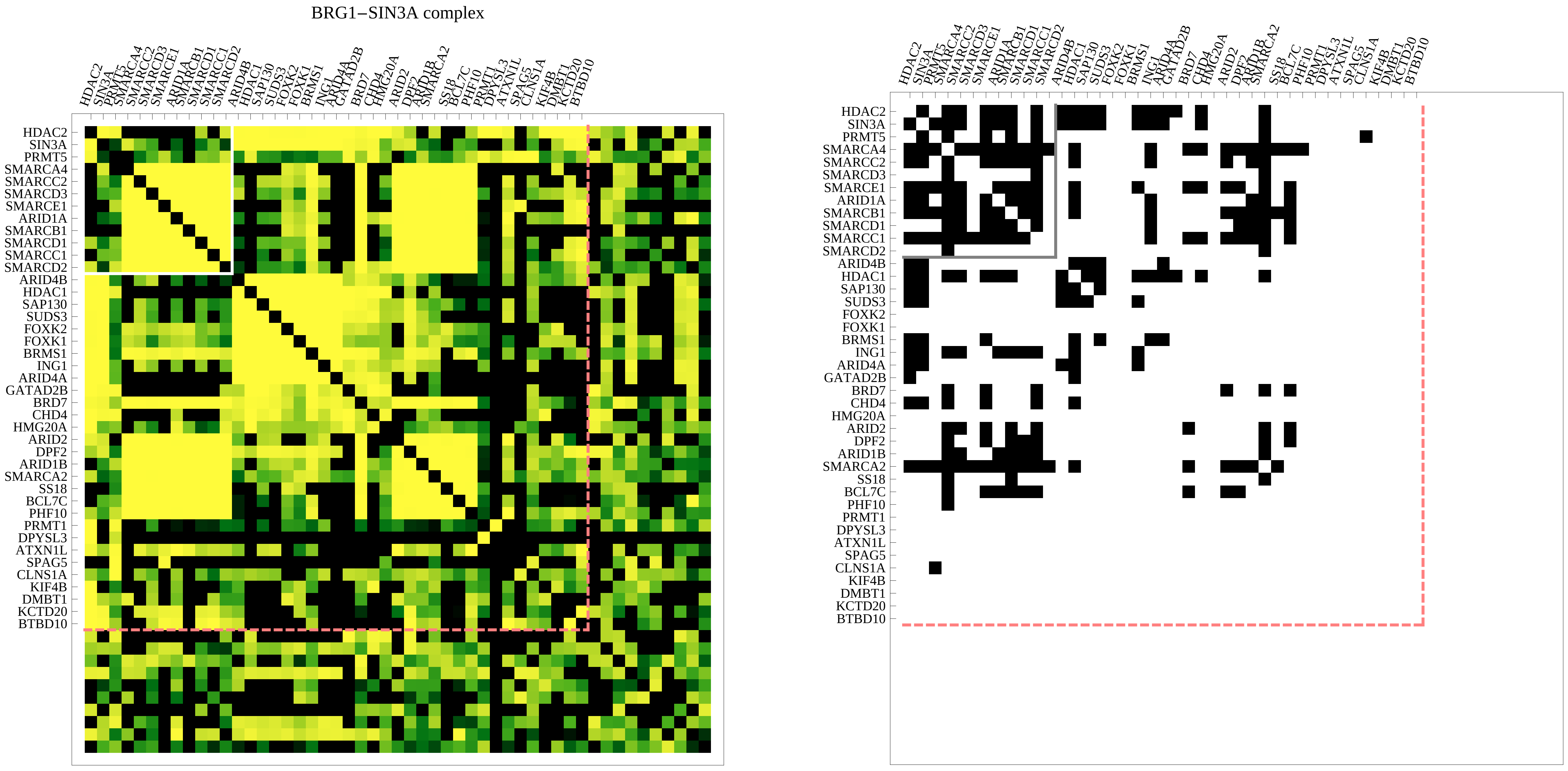
BRG1−SIN3A complex
HDAC2
SIN3A
PRMT5
SMARCA4
SMARCC2
SMARCD3
SMARCE1
ARID1A
SMARCB1
SMARCD1
SMARCC1
SMARCD2
ARID4B
HDAC1
SAP130
SUDS3
FOXK2
FOXK1
BRMS1
ING1
ARID4A
GATAD2B
BRD7
CHD4
HMG20A
ARID2
DPF2
ARID1B
SMARCA2
SS18
BCL7C
PHF10
PRMT1
DPYSL3
ATXN1L
SPAG5
CLNS1A
KIF4B
DMBT1
KCTD20
BTBD10

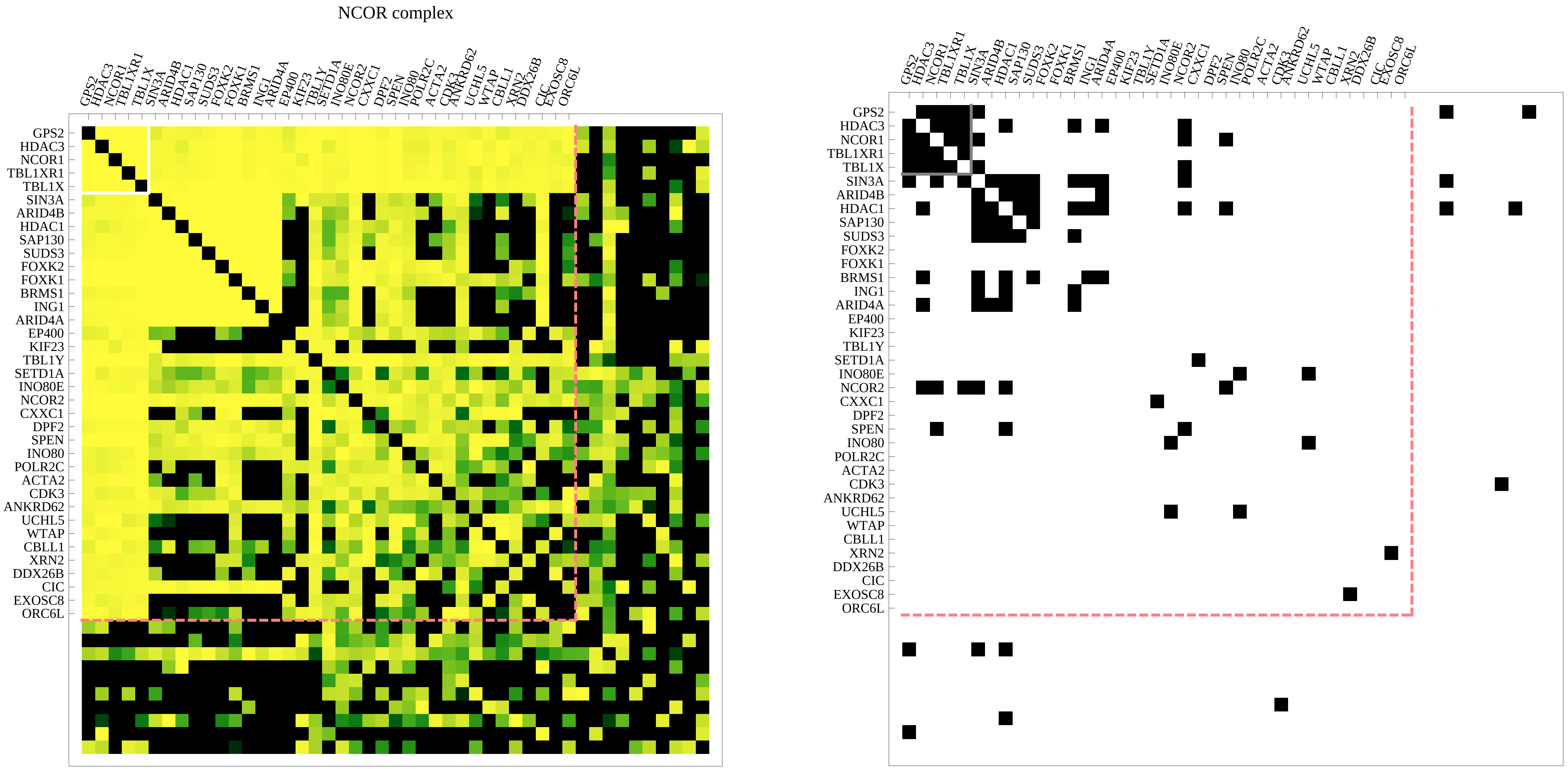
NCOR complex
GPS2
HDAC3
NCOR1
TBL1XR1
TBL1X
SIN3A
ARID4B
HDAC1
SAP130
SUDS3
FOXK2
FOXK1
BRMS1
ING1
ARID4A
EP400
KIF23
TBL1Y
SETD1A
INO80E
NCOR2
CXXC1
DPF2
SPEN
INO80
POLR2C
ACTA2
CDK3
ANKRD62
UCHL5
WTAP
CBLL1
XRN2
DDX26B
CIC
EXOSC8
ORC6L

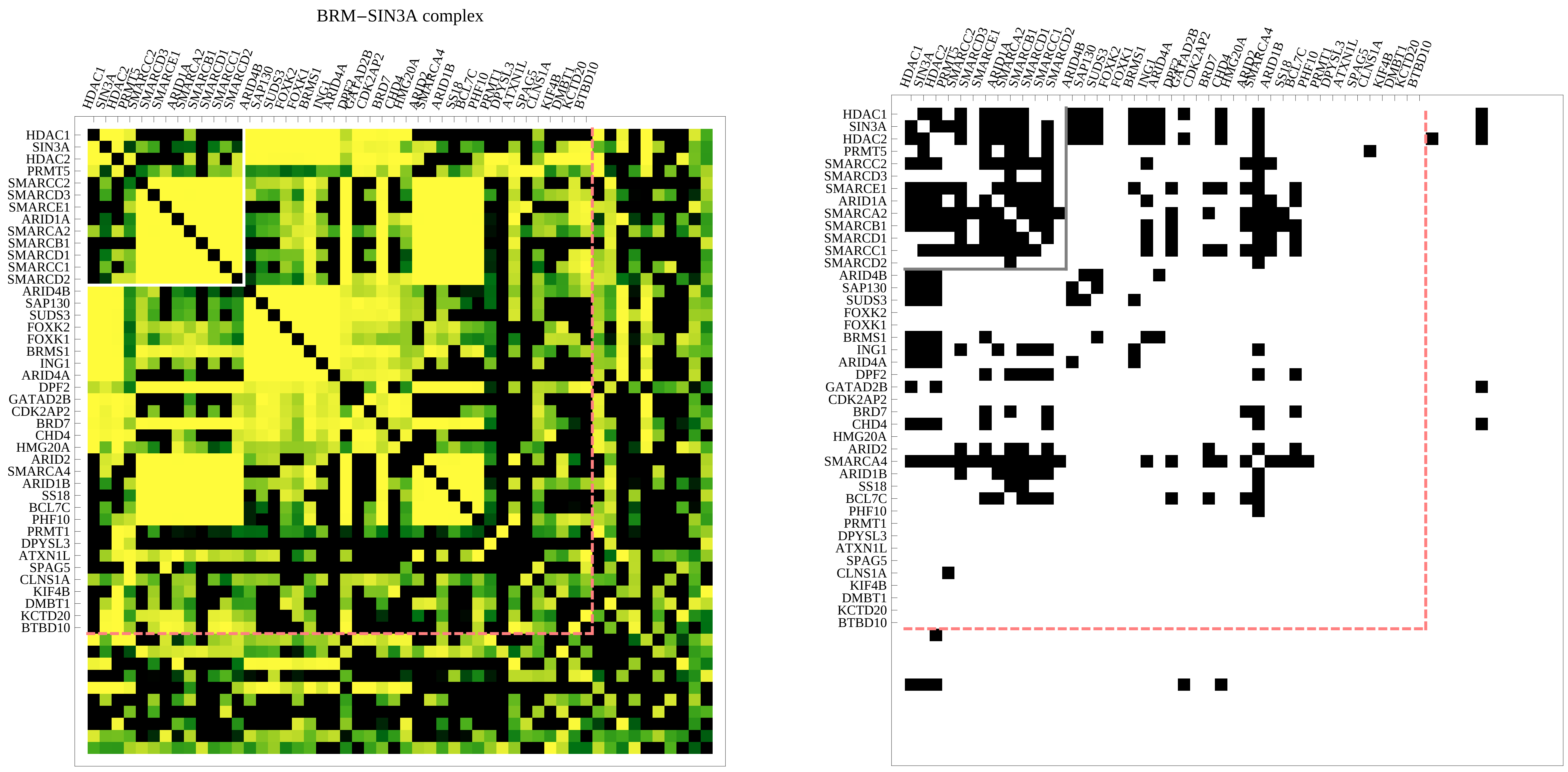
BRM−SIN3A complex
HDAC1
SIN3A
HDAC2
PRMT5
SMARCC2
SMARCD3
SMARCE1
ARID1A
SMARCA2
SMARCB1
SMARCD1
SMARCC1
SMARCD2
ARID4B
SAP130
SUDS3
FOXK2
FOXK1
BRMS1
ING1
ARID4A
DPF2
GATAD2B
CDK2AP2
BRD7
CHD4
HMG20A
ARID2
SMARCA4
ARID1B
SS18
BCL7C
PHF10
PRMT1
DPYSL3
ATXN1L
SPAG5
CLNS1A
KIF4B
DMBT1
KCTD20
BTBD10

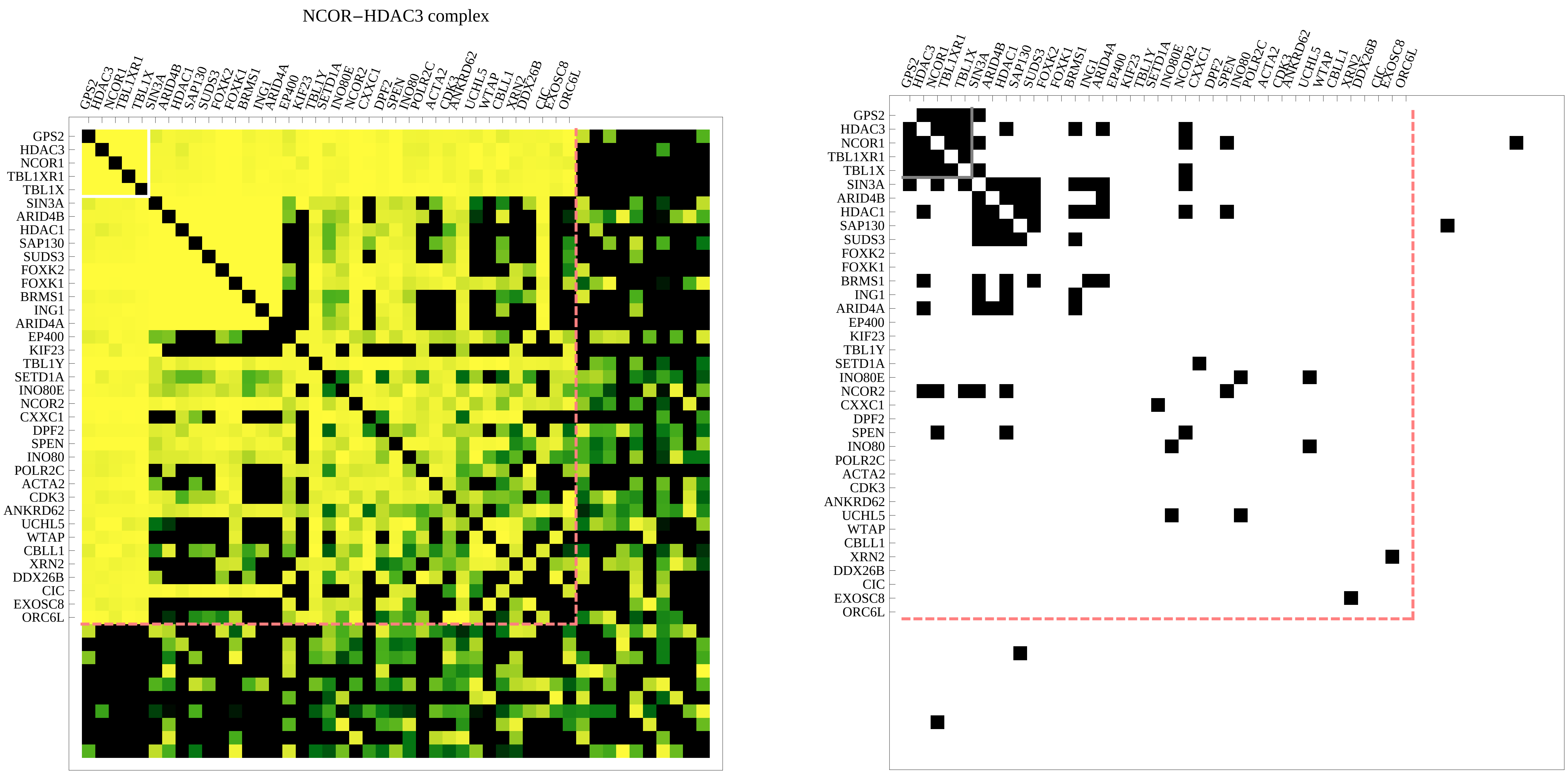
NCOR−HDAC3 complex
GPS2
HDAC3
NCOR1
TBL1XR1
TBL1X
SIN3A
ARID4B
HDAC1
SAP130
SUDS3
FOXK2
FOXK1
BRMS1
ING1
ARID4A
EP400
KIF23
TBL1Y
SETD1A
INO80E
NCOR2
CXXC1
DPF2
SPEN
INO80
POLR2C
ACTA2
CDK3
ANKRD62
UCHL5
WTAP
CBLL1
XRN2
DDX26B
CIC
EXOSC8
ORC6L

x (EIF3S6, EIF3S5, EIF3S4, EIF3S3, EIF3S6IP, EIF3S2, EIF3S9, EIF3S12, EIF3S10, EIF

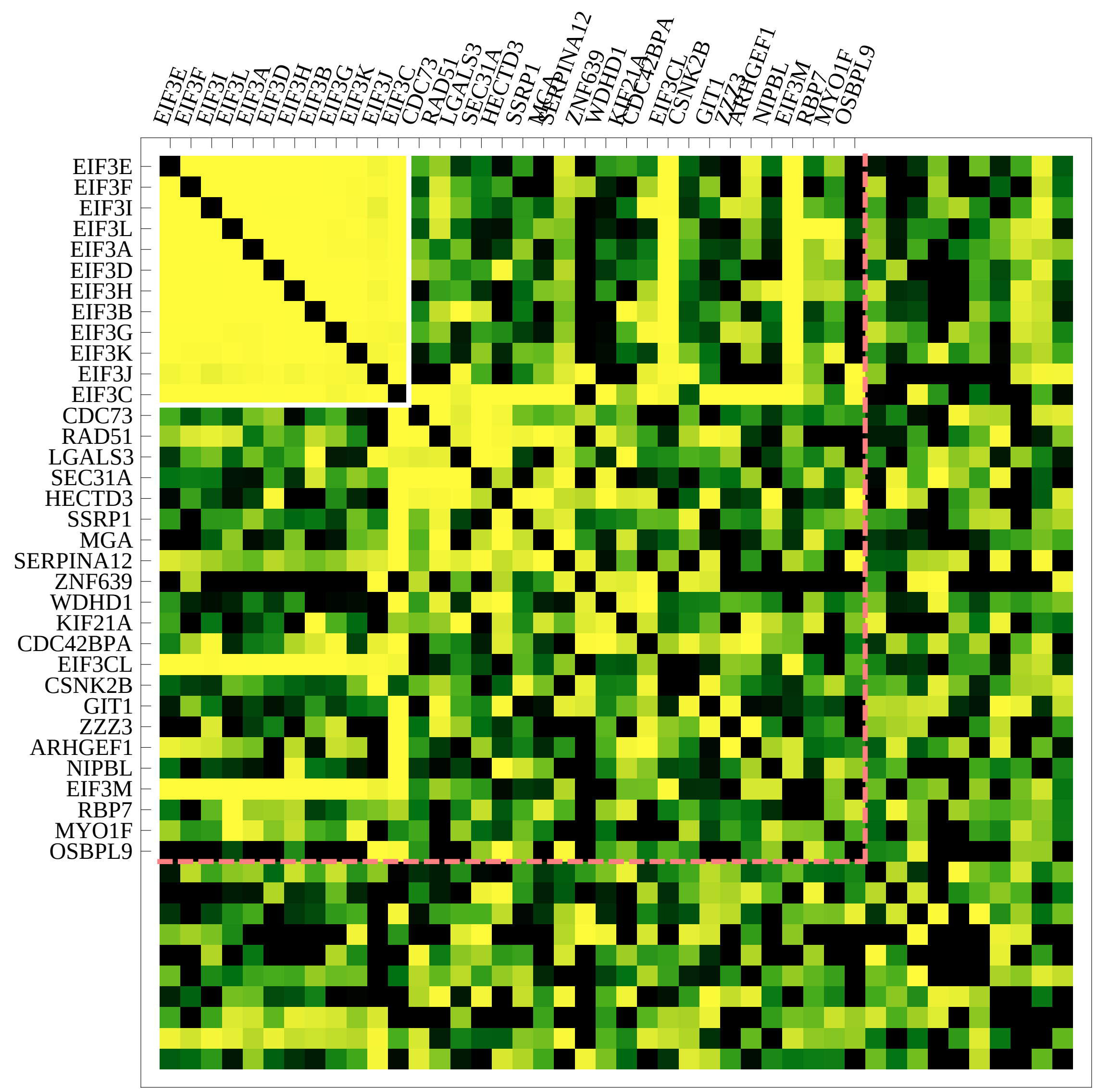

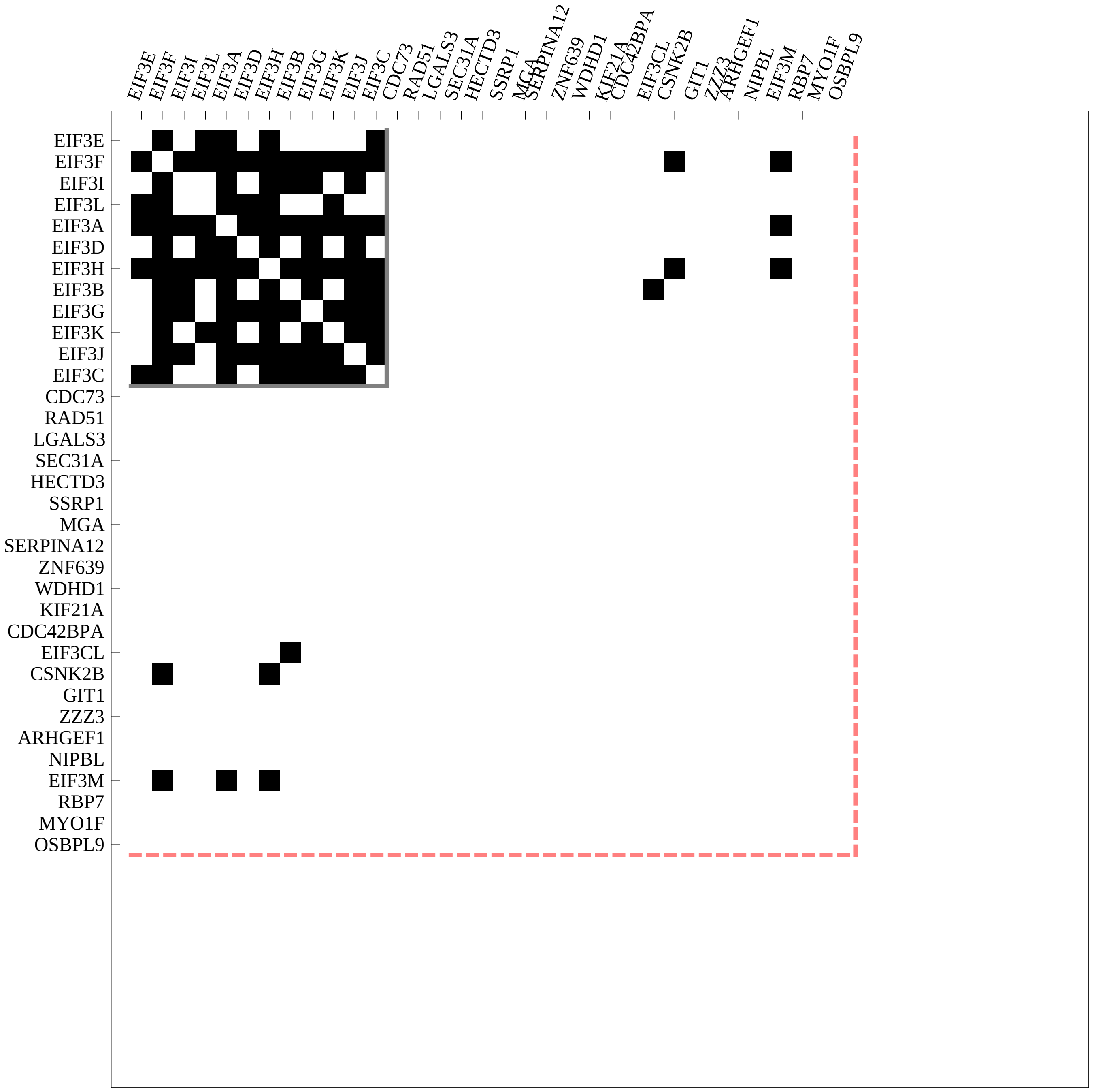

LARC complex (LCR−associated remodeling complex)

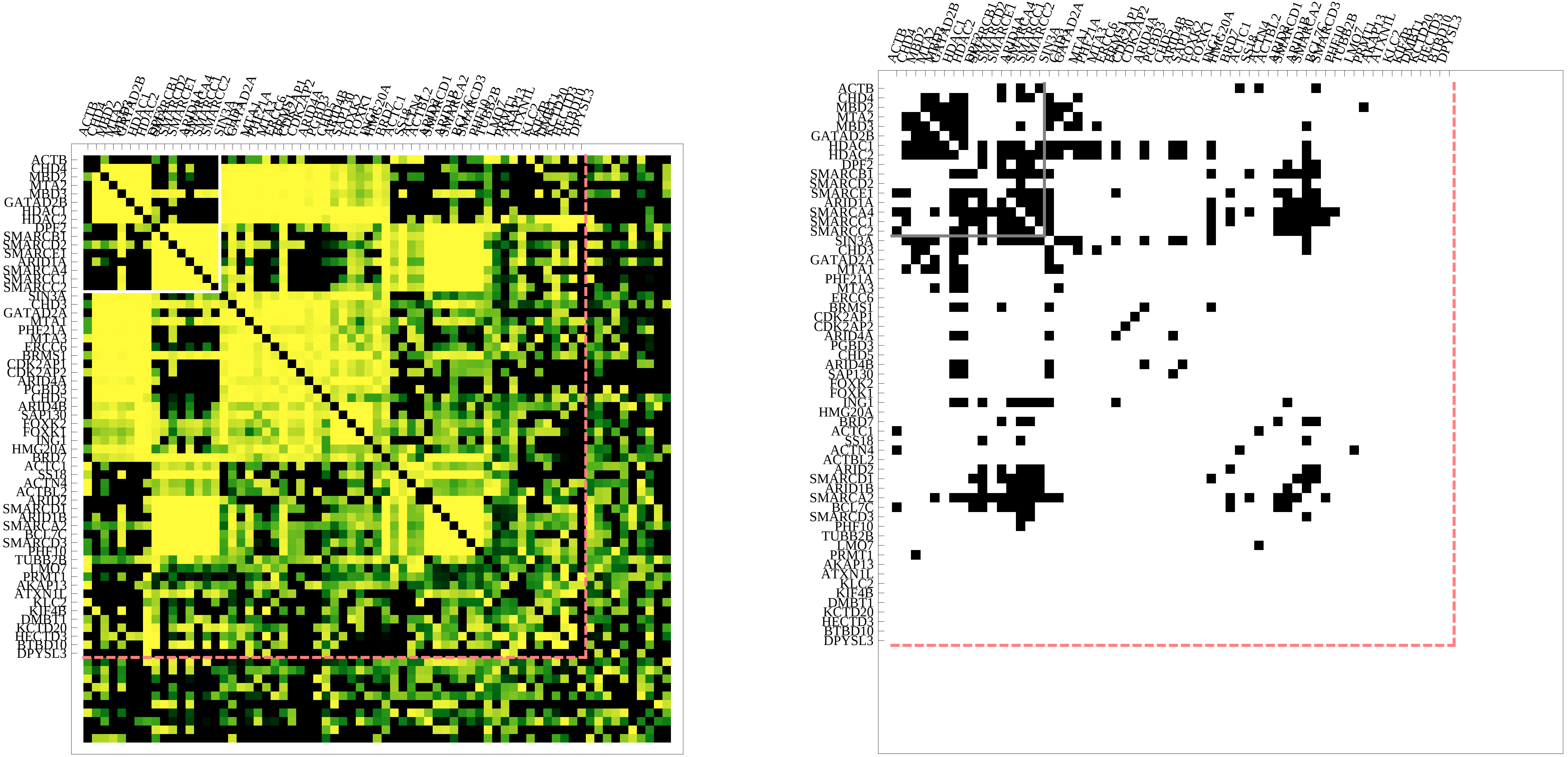

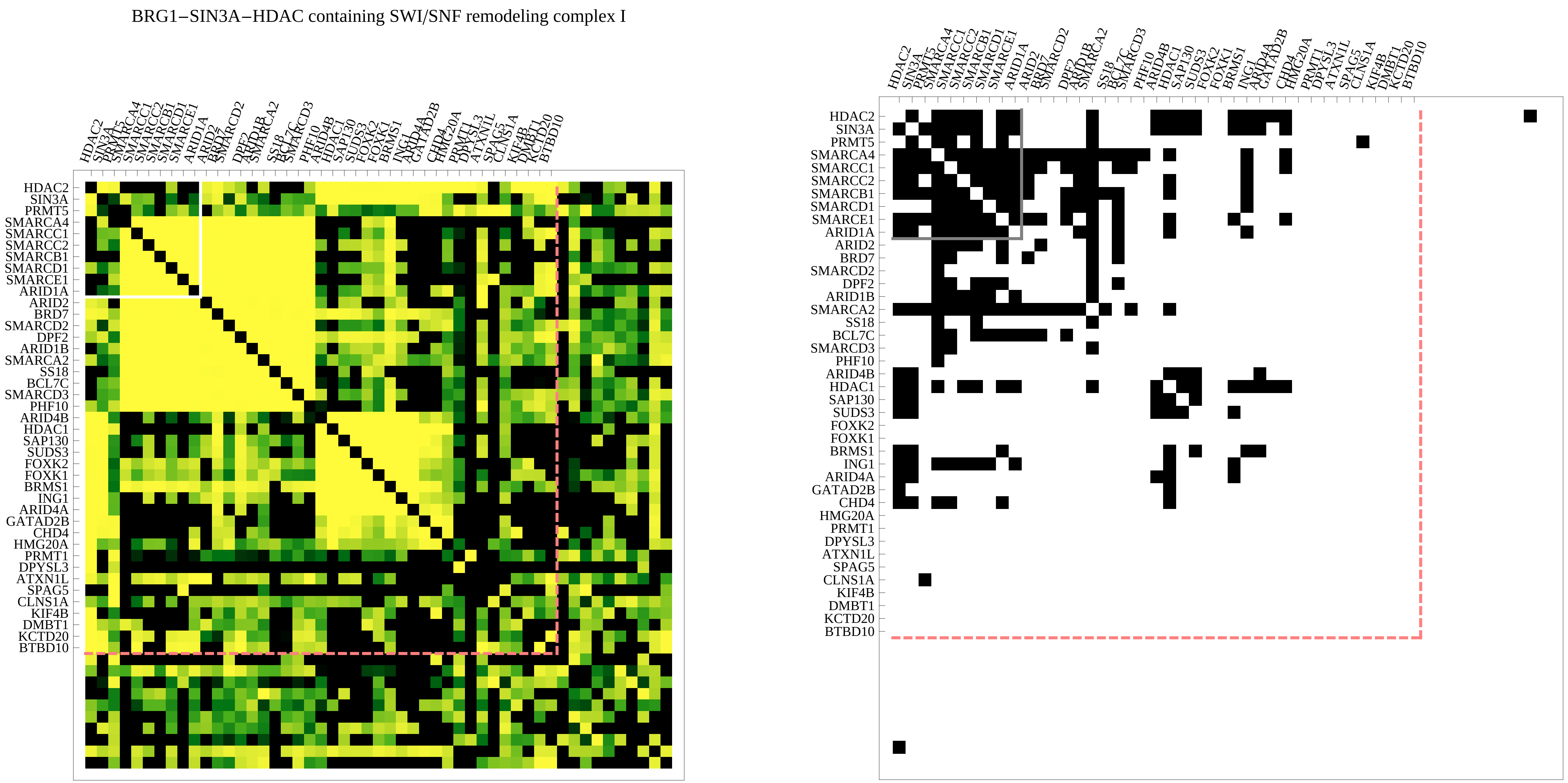
BRG1−SIN3A−HDAC containing SWI/SNF remodeling complex I
HDAC2
SIN3A
PRMT5
SMARCA4
SMARCC1
SMARCC2
SMARCB1
SMARCD1
SMARCE1
ARID1A
ARID2
BRD7
SMARCD2
DPF2
ARID1B
SMARCA2
SS18
BCL7C
SMARCD3
PHF10
ARID4B
HDAC1
SAP130
SUDS3
FOXK2
FOXK1
BRMS1
ING1
ARID4A
GATAD2B
CHD4
HMG20A
PRMT1
DPYSL3
ATXN1L
SPAG5
CLNS1A
KIF4B
DMBT1
KCTD20
BTBD10

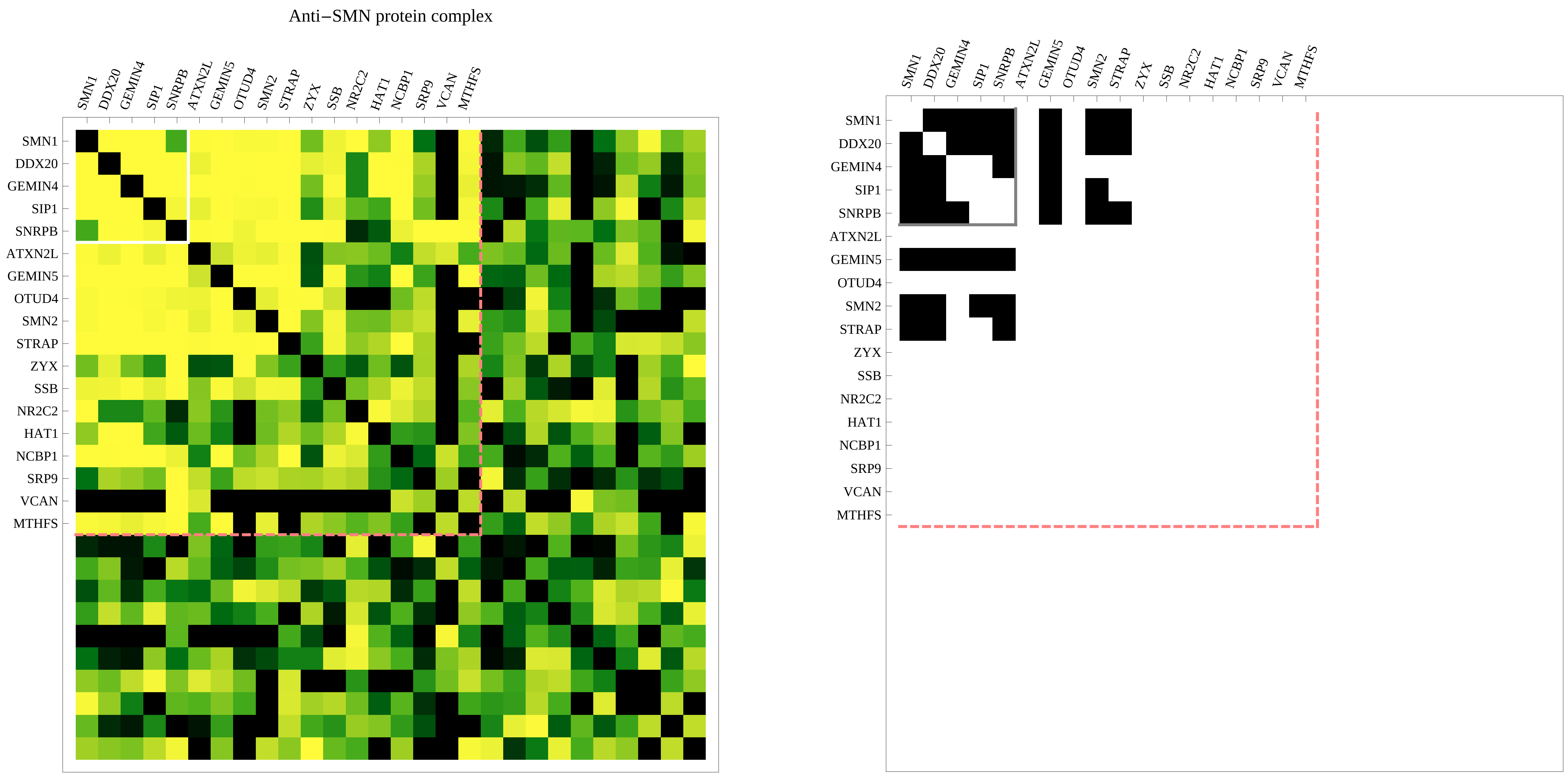

Anti−SMN protein complex
SMN1
DDX20
GEMIN4
SIP1
SNRPB
ATXN2L
GEMIN5
OTUD4
SMN2
STRAP
ZYX
SSB
NR2C2
HAT1
NCBP1
SRP9
VCAN
MTHFS

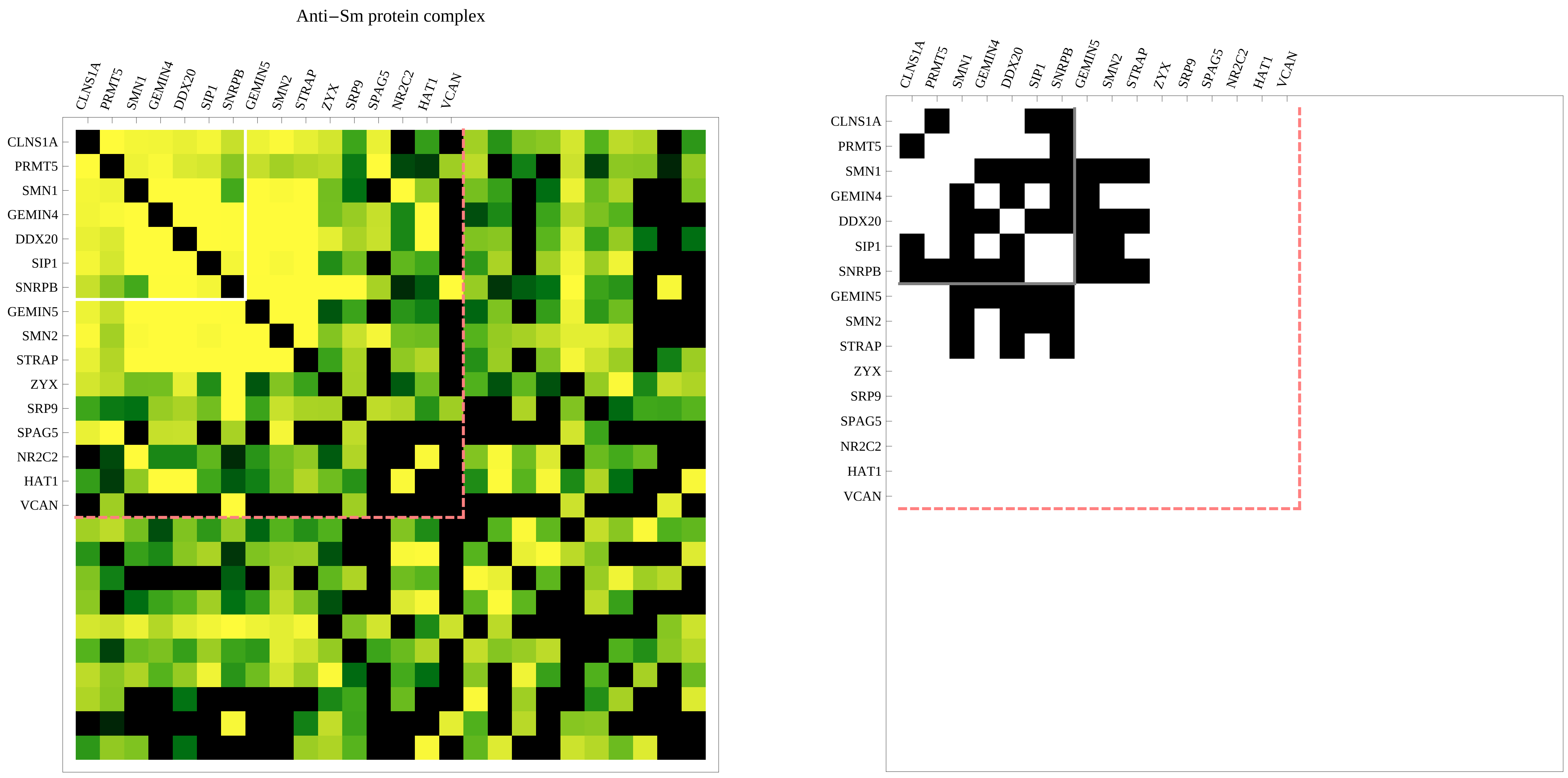

Anti−Sm protein complex
CLNS1A
PRMT5
SMN1
GEMIN4
DDX20
SIP1
SNRPB
GEMIN5
SMN2
STRAP
ZYX
SRP9
SPAG5
NR2C2
HAT1
VCAN

20S methylosome and RG−containing Sm protein complex

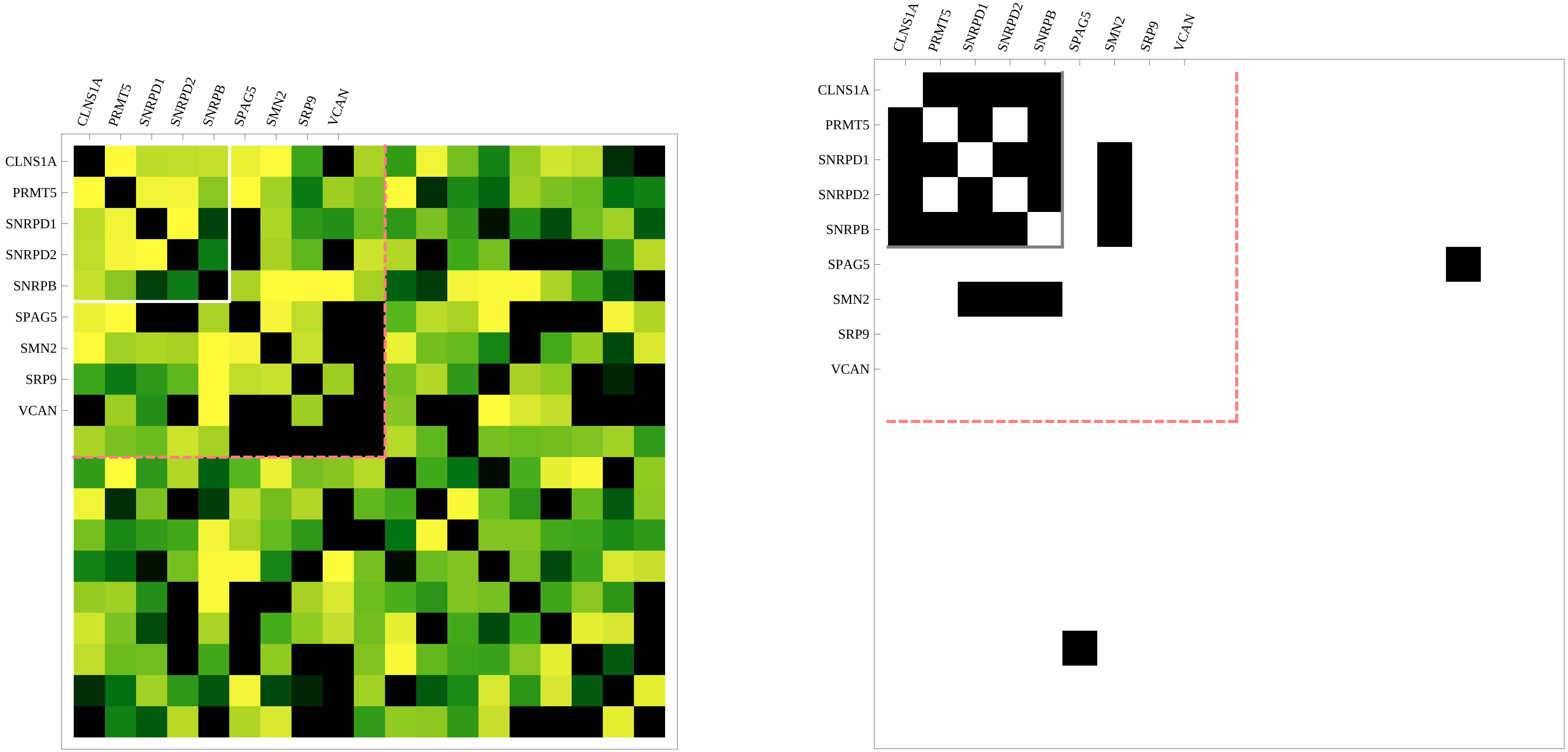

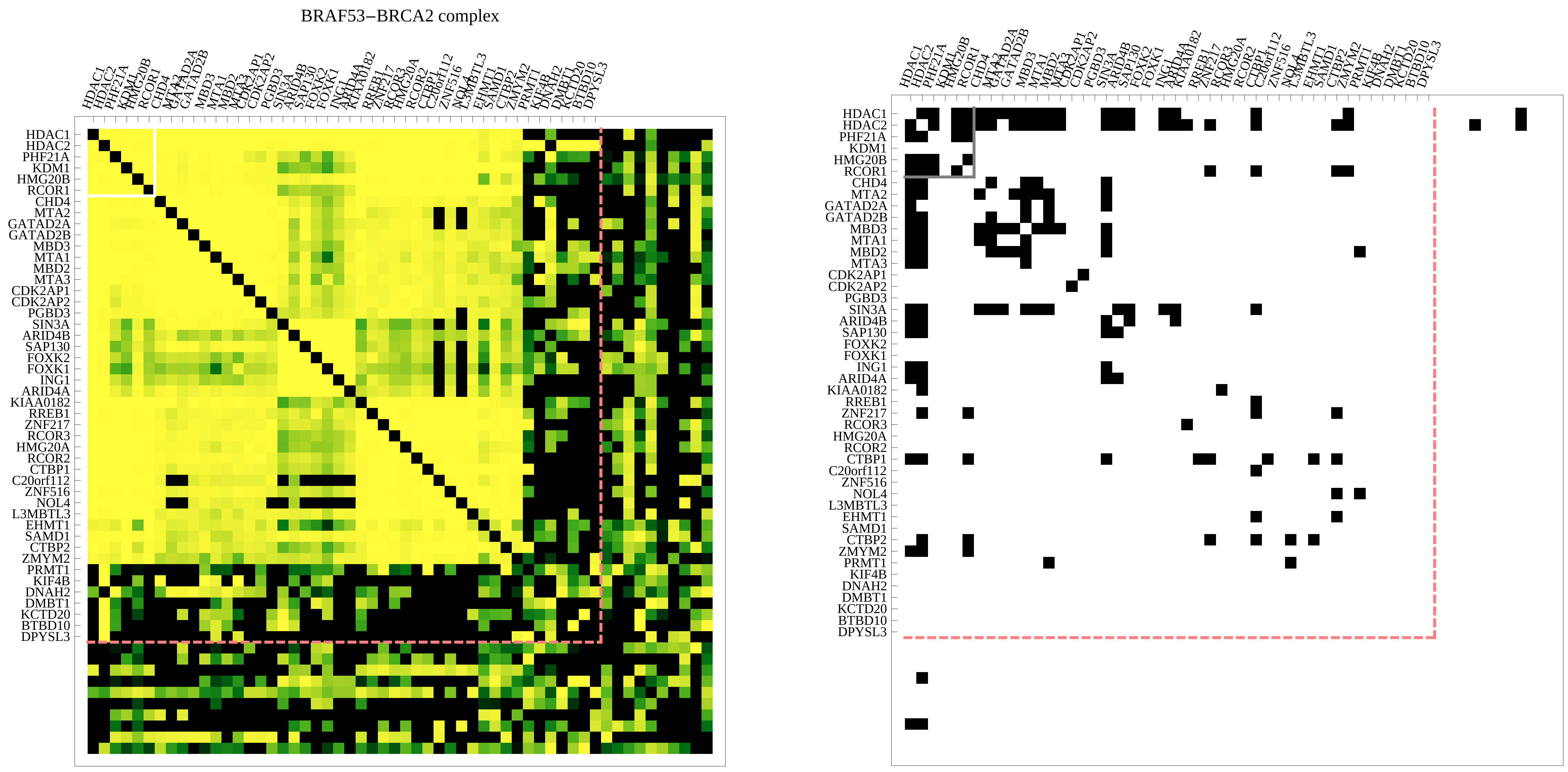

BRAF53−BRCA2 complex
HDAC1
HDAC2
PHF21A
KDM1
HMG20B
RCOR1
CHD4
MTA2
GATAD2A
GATAD2B
MBD3
MTA1
MBD2
MTA3
CDK2AP1
CDK2AP2
PGBD3
SIN3A
ARID4B
SAP130
FOXK2
FOXK1
ING1
ARID4A
KIAA0182
RREB1
ZNF217
RCOR3
HMG20A
RCOR2
CTBP1
C20orf112
ZNF516
NOL4
L3MBTL3
EHMT1
SAMD1
CTBP2
ZMYM2
PRMT1
KIF4B
DNAH2
DMBT1
KCTD20
BTBD10
DPYSL3

TFIIH transcription factor complex

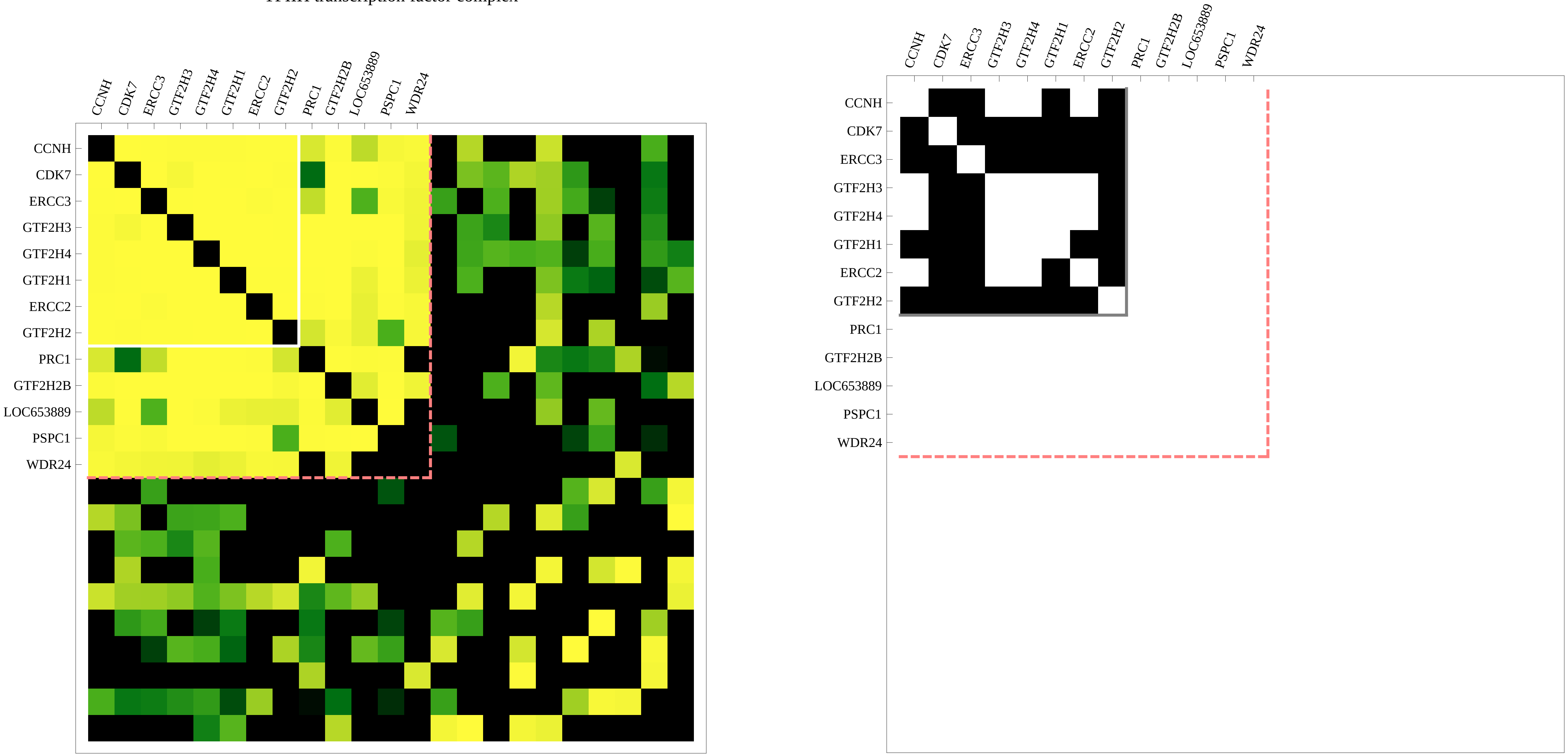

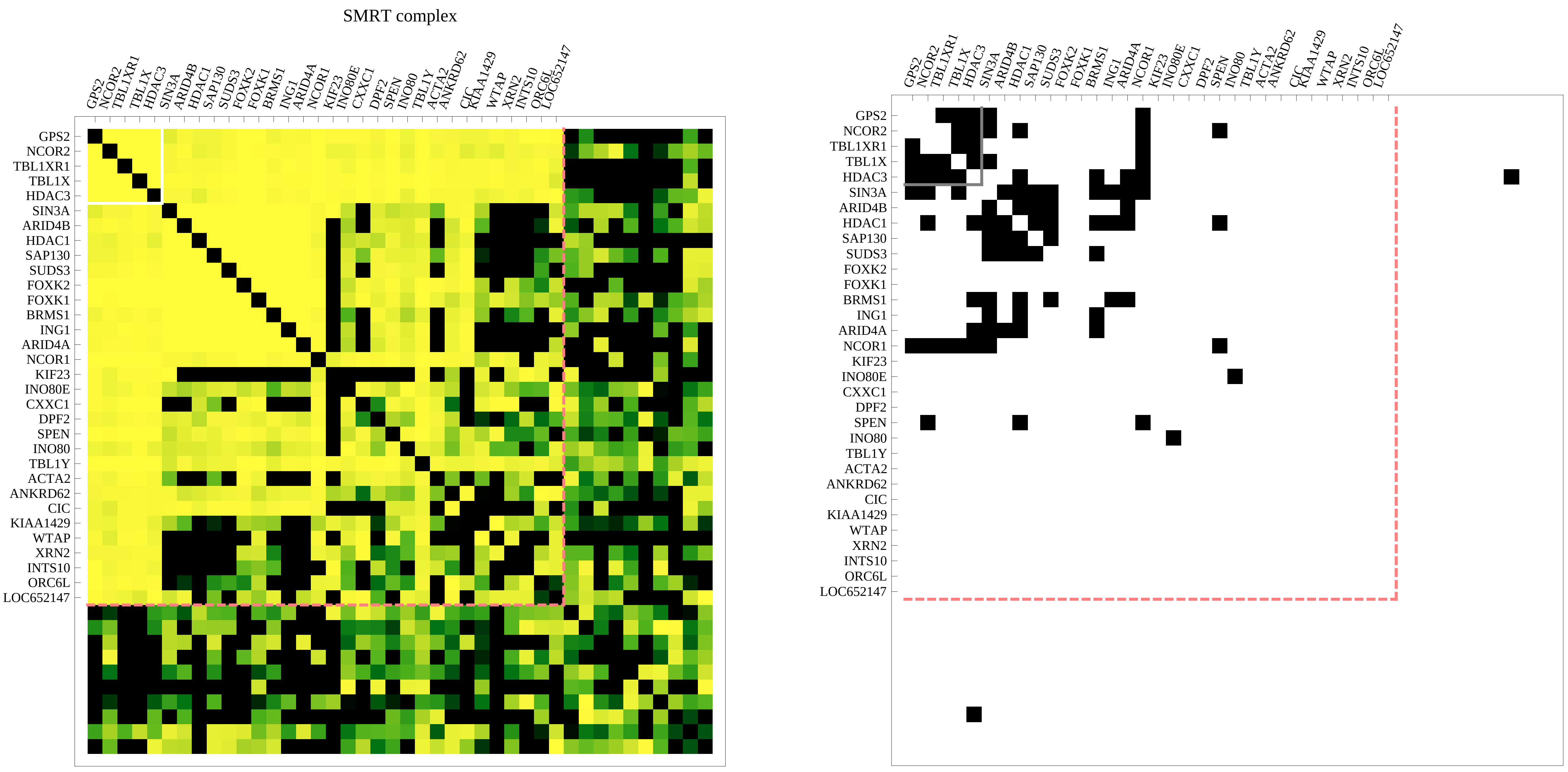
SMRT complex
GPS2
NCOR2
TBL1XR1
TBL1X
HDAC3
SIN3A
ARID4B
HDAC1
SAP130
SUDS3
FOXK2
FOXK1
BRMS1
ING1
ARID4A
NCOR1
KIF23
INO80E
CXXC1
DPF2
SPEN
INO80
TBL1Y
ACTA2
ANKRD62
CIC
KIAA1429
WTAP
XRN2
INTS10
ORC6L
LOC652147

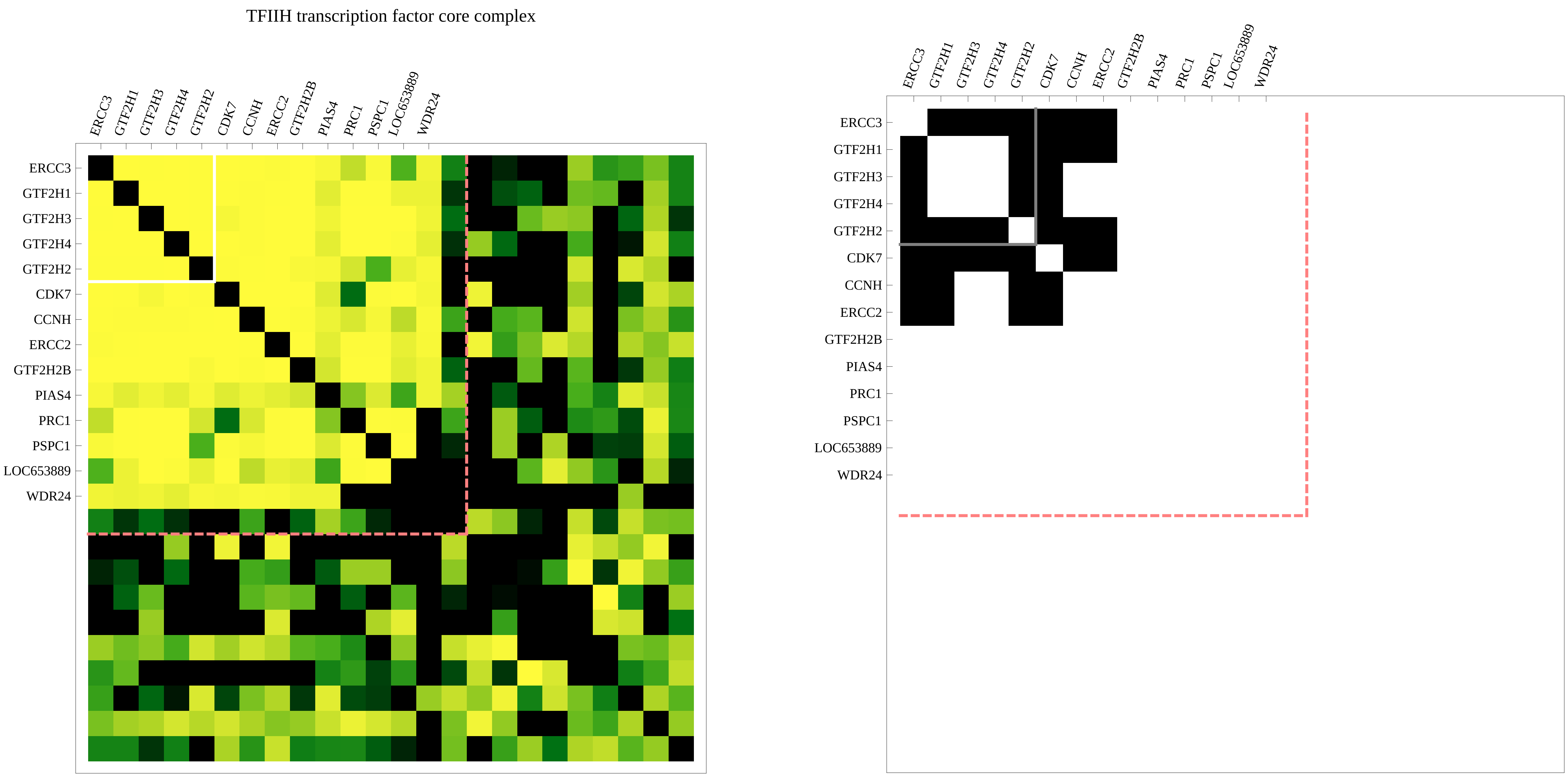
TFIIH transcription factor core complex
ERCC3
GTF2H1
GTF2H3
GTF2H4
GTF2H2
CDK7
CCNH
ERCC2
GTF2H2B
PIAS4
PRC1
PSPC1
LOC653889
WDR24

F3S6, EIF3S5, EIF3S4, EIF3S3, EIF3S6IP, EIF3S2, EIF3S9, EIF3S12, EIF3S10, EIF3S

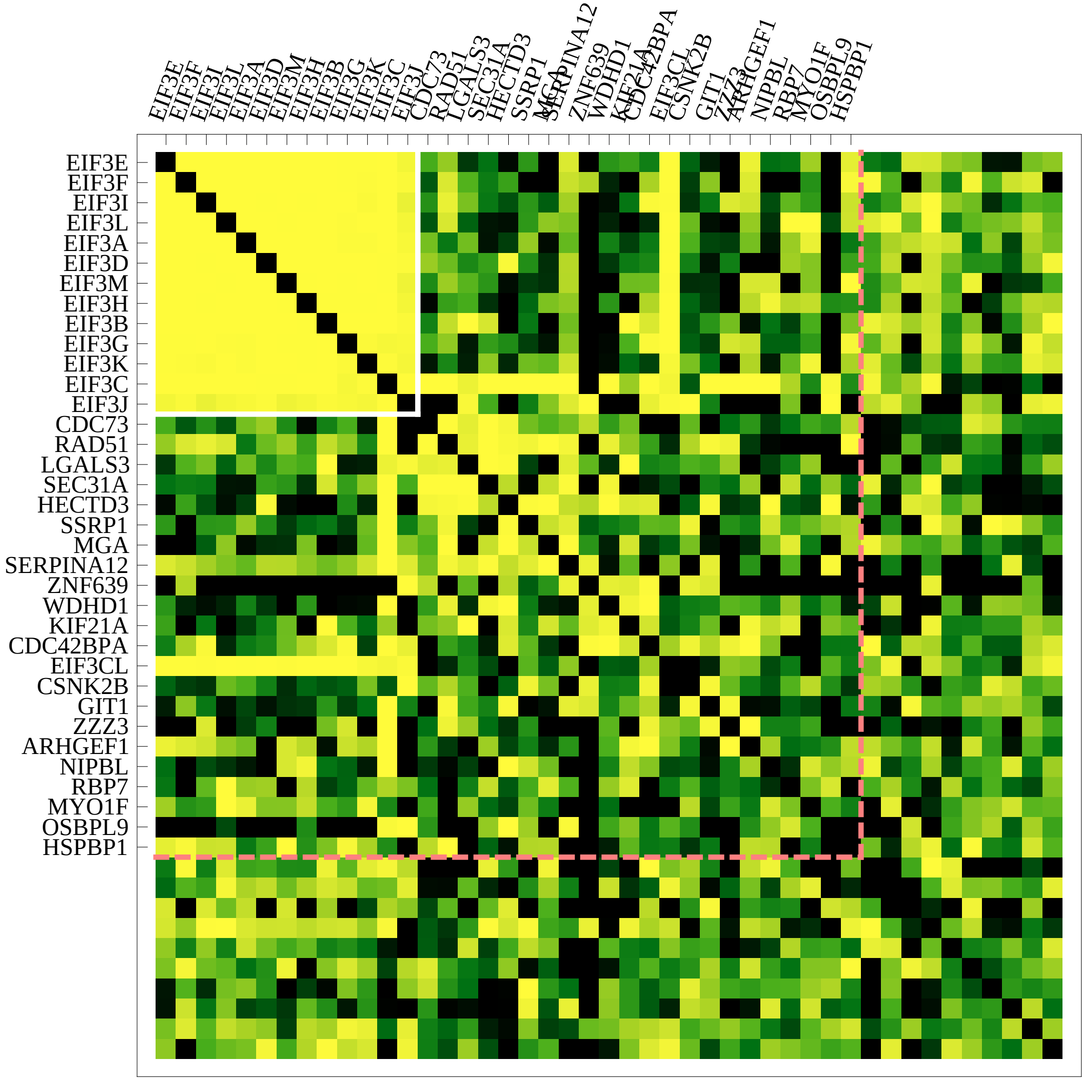

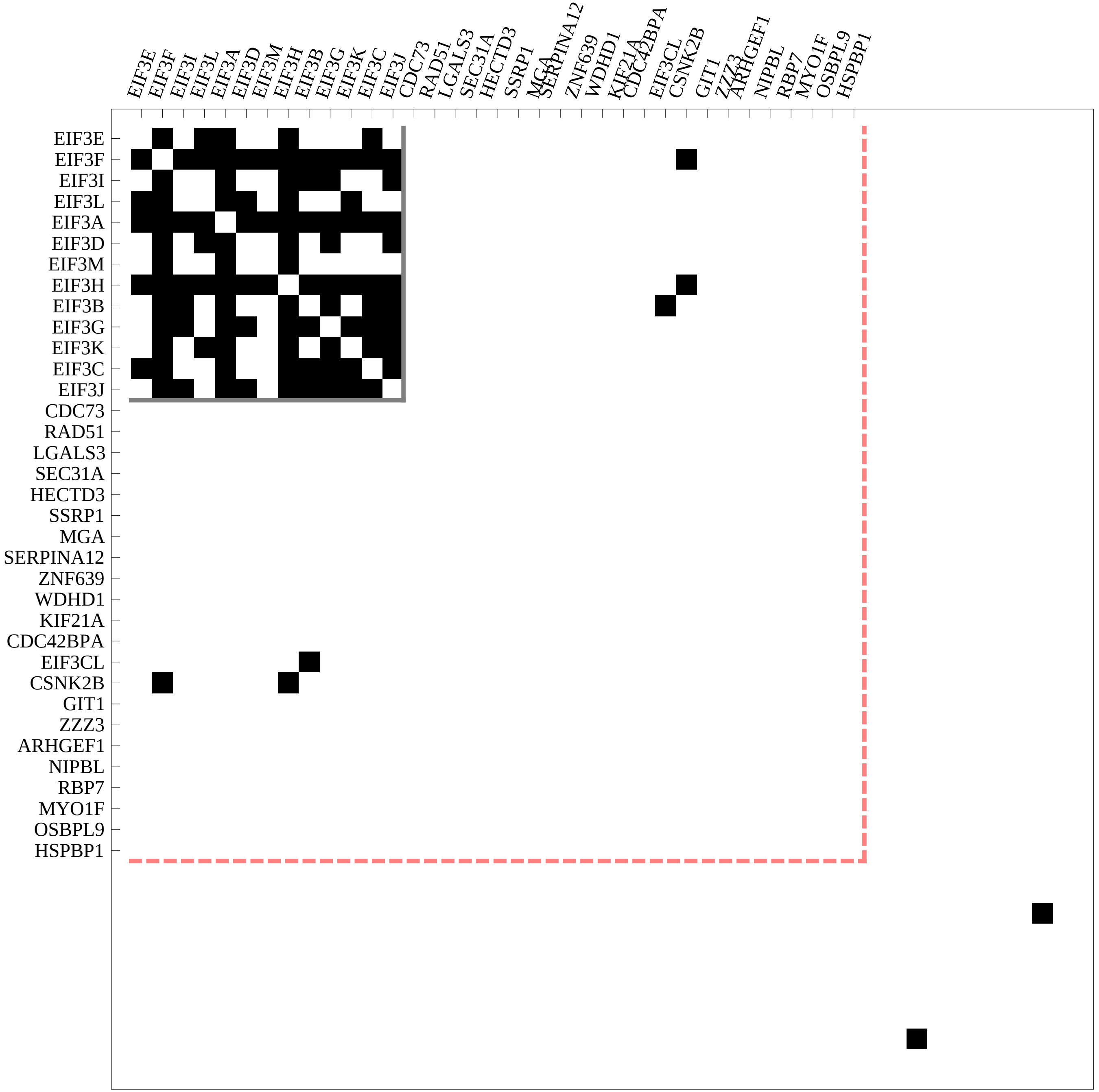

Polyadenylation complex (CSTF1, CSTF2, CSTF3, SYMPK CPSF1, CPSF2, CPSF3[illegible]

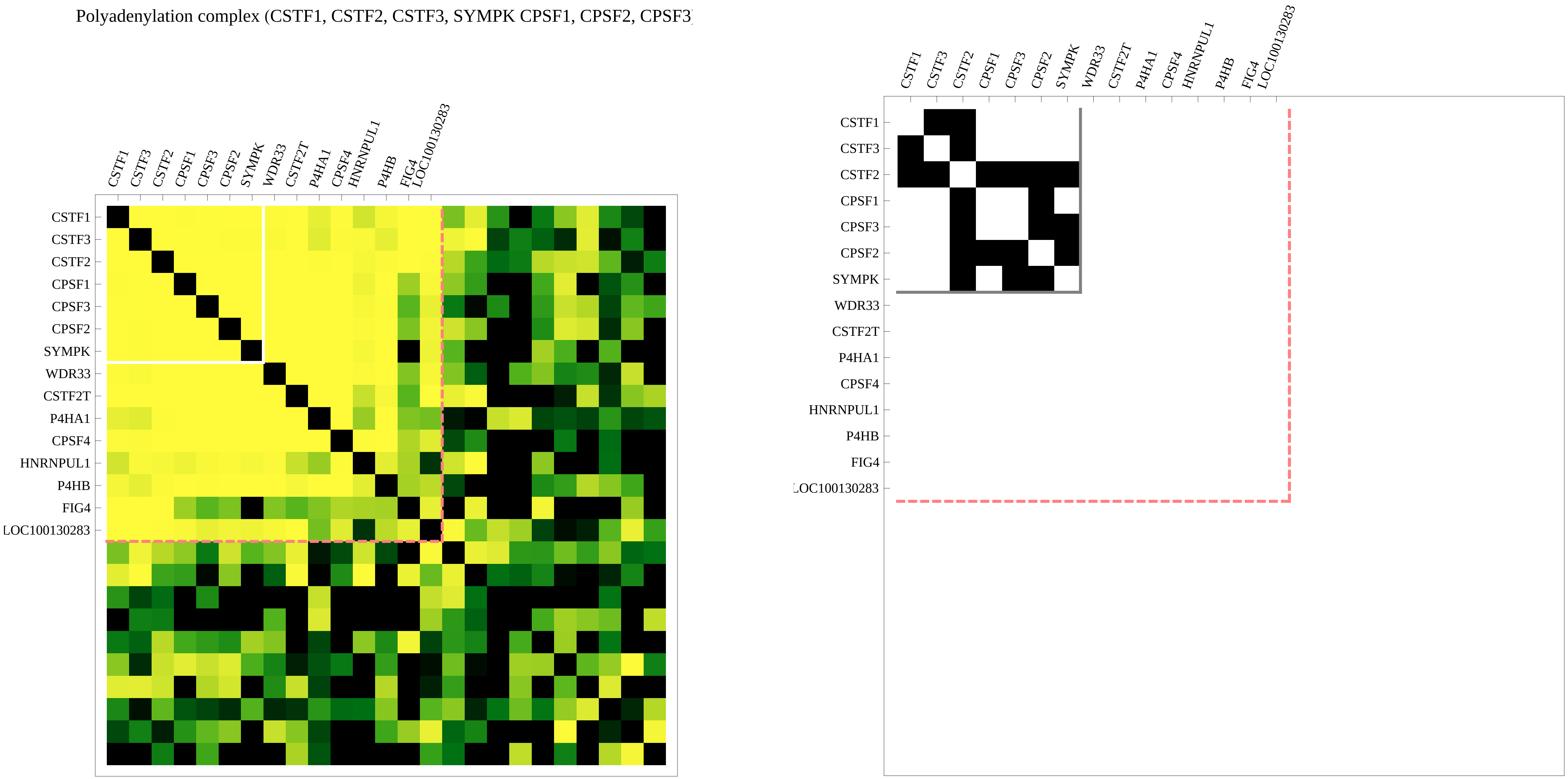

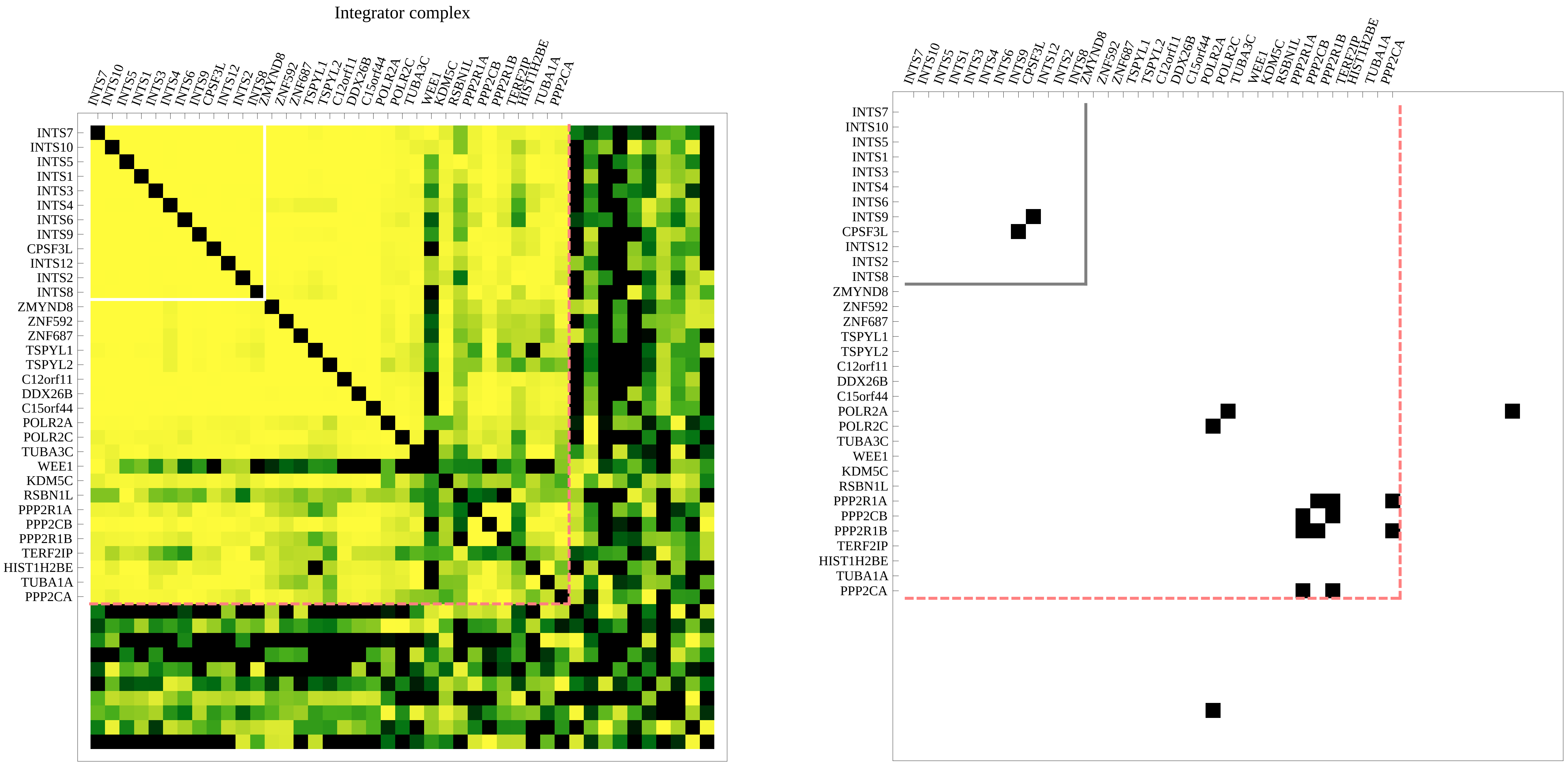
Integrator complex
INTS7
INTS10
INTS5
INTS1
INTS3
INTS4
INTS6
INTS9
CPSF3L
INTS12
INTS2
INTS8
ZMYND8
ZNF592
ZNF687
TSPYL1
TSPYL2
C12orf11
DDX26B
C15orf44
POLR2A
POLR2C
TUBA3C
WEE1
KDM5C
RSBN1L
PPP2R1A
PPP2CB
PPP2R1B
TERF2IP
HIST1H2BE
TUBA1A
PPP2CA

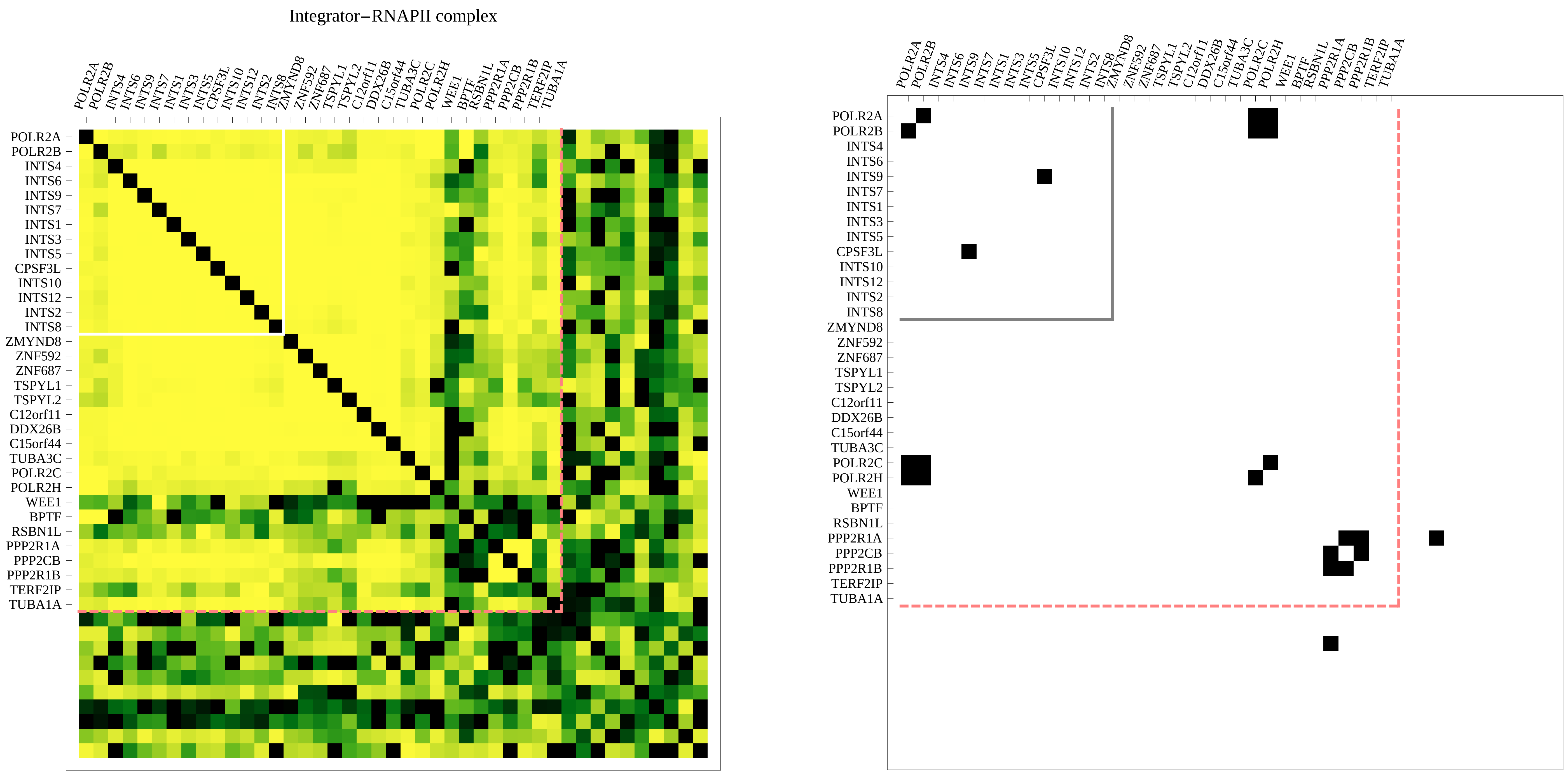

Integrator−RNAPII complex
POLR2A
POLR2B
INTS4
INTS6
INTS9
INTS7
INTS1
INTS3
INTS5
CPSF3L
INTS10
INTS12
INTS2
INTS8
ZMYND8
ZNF592
ZNF687
TSPYL1
TSPYL2
C12orf11
DDX26B
C15orf44
TUBA3C
POLR2C
POLR2H
WEE1
BPTF
RSBN1L
PPP2R1A
PPP2CB
PPP2R1B
TERF2IP
TUBA1A
POLR2A
POLR2B
INTS4
INTS6
INTS9
INTS7
INTS1
INTS3
INTS5
CPSF3L
INTS10
INTS12
INTS2
INTS8
ZMYND8
ZNF592
ZNF687
TSPYL1
TSPYL2
C12orf11
DDX26B
C15orf44
TUBA3C
POLR2C
POLR2H
WEE1
BPTF
RSBN1L
PPP2R1A
PPP2CB
PPP2R1B
TERF2IP
TUBA1A

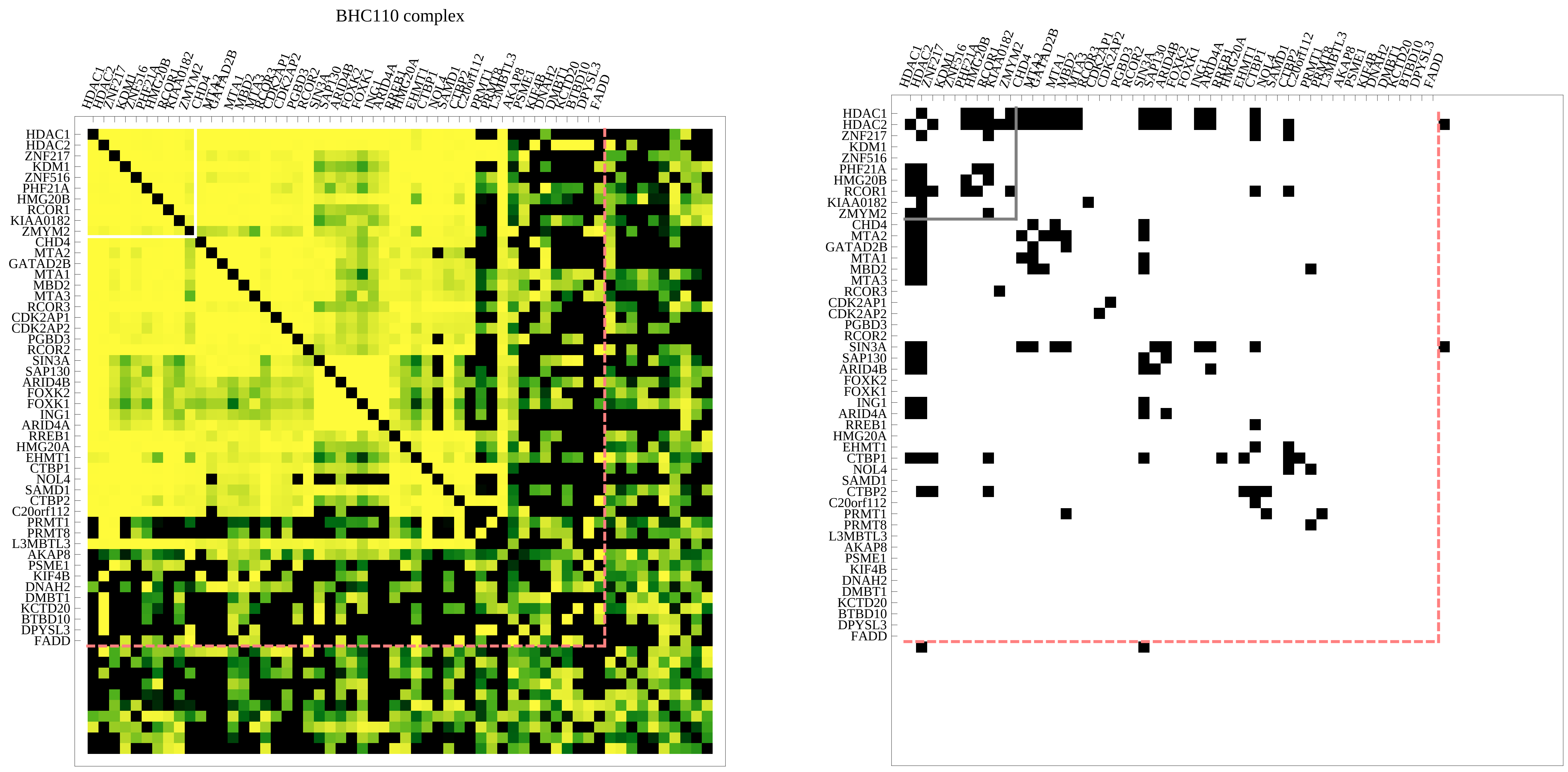
BHC110 complex
HDAC1
HDAC2
ZNF217
KDM1
ZNF516
PHF21A
HMG20B
RCOR1
KIAA0182
ZMYM2
CHD4
MTA2
GATAD2B
MTA1
MBD2
MTA3
RCOR3
CDK2AP1
CDK2AP2
PGBD3
RCOR2
SIN3A
SAP130
ARID4B
FOXK2
FOXK1
ING1
ARID4A
RREB1
HMG20A
EHMT1
CTBP1
NOL4
SAMD1
CTBP2
C20orf112
PRMT1
PRMT8
L3MBTL3
AKAP8
PSME1
KIF4B
DNAH2
DMBT1
KCTD20
BTBD10
DPYSL3
FADD

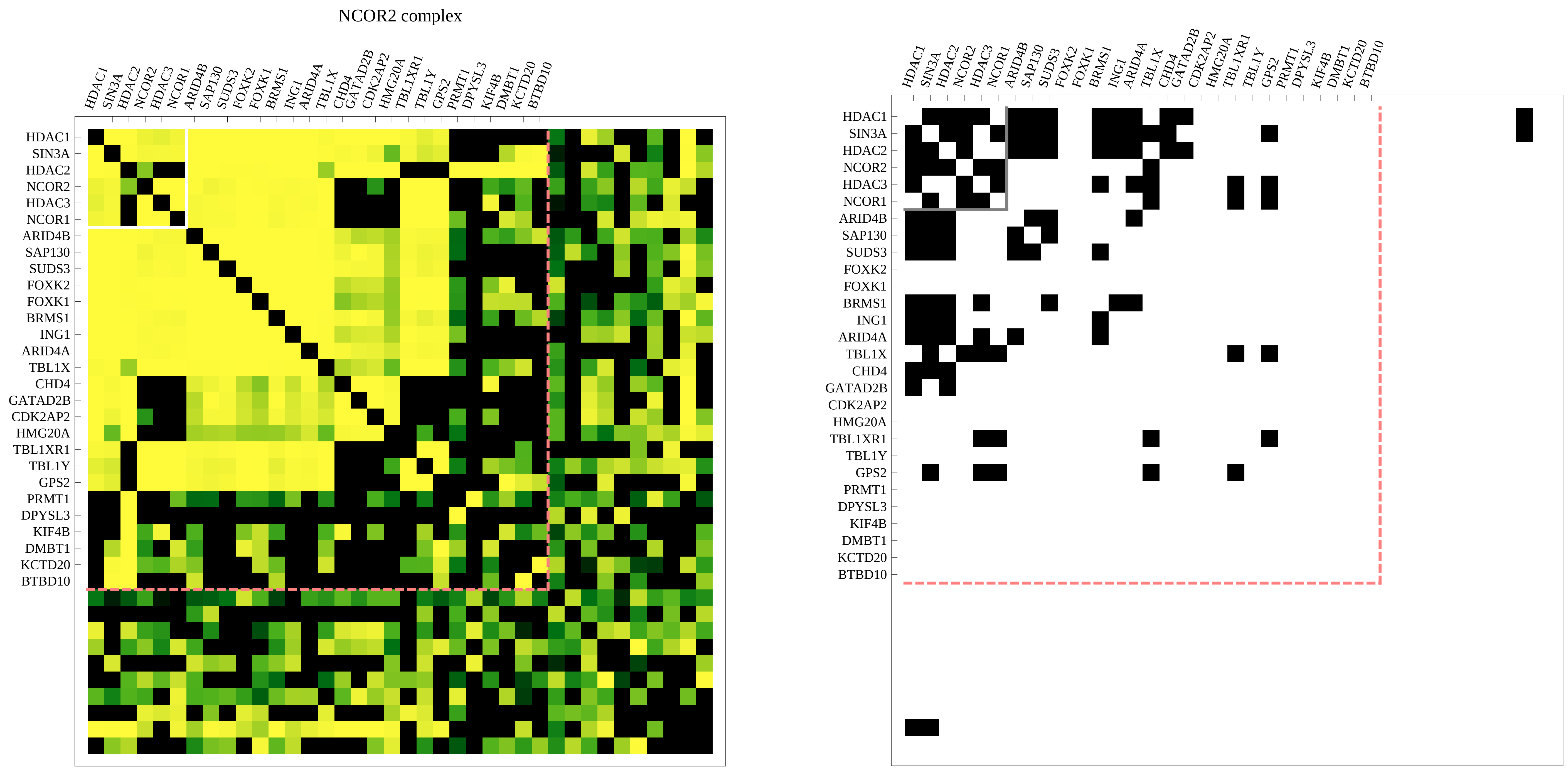

NCOR2 complex
HDAC1
SIN3A
HDAC2
NCOR2
HDAC3
NCOR1
ARID4B
SAP130
SUDS3
FOXK2
FOXK1
BRMS1
ING1
ARID4A
TBL1X
CHD4
GATAD2B
CDK2AP2
HMG20A
TBL1XR1
TBL1Y
GPS2
PRMT1
DPYSL3
KIF4B
DMBT1
KCTD20
BTBD10

CDCA5−PDS5A−RAD21−SMC1A−PDS5B−SMC3 complex

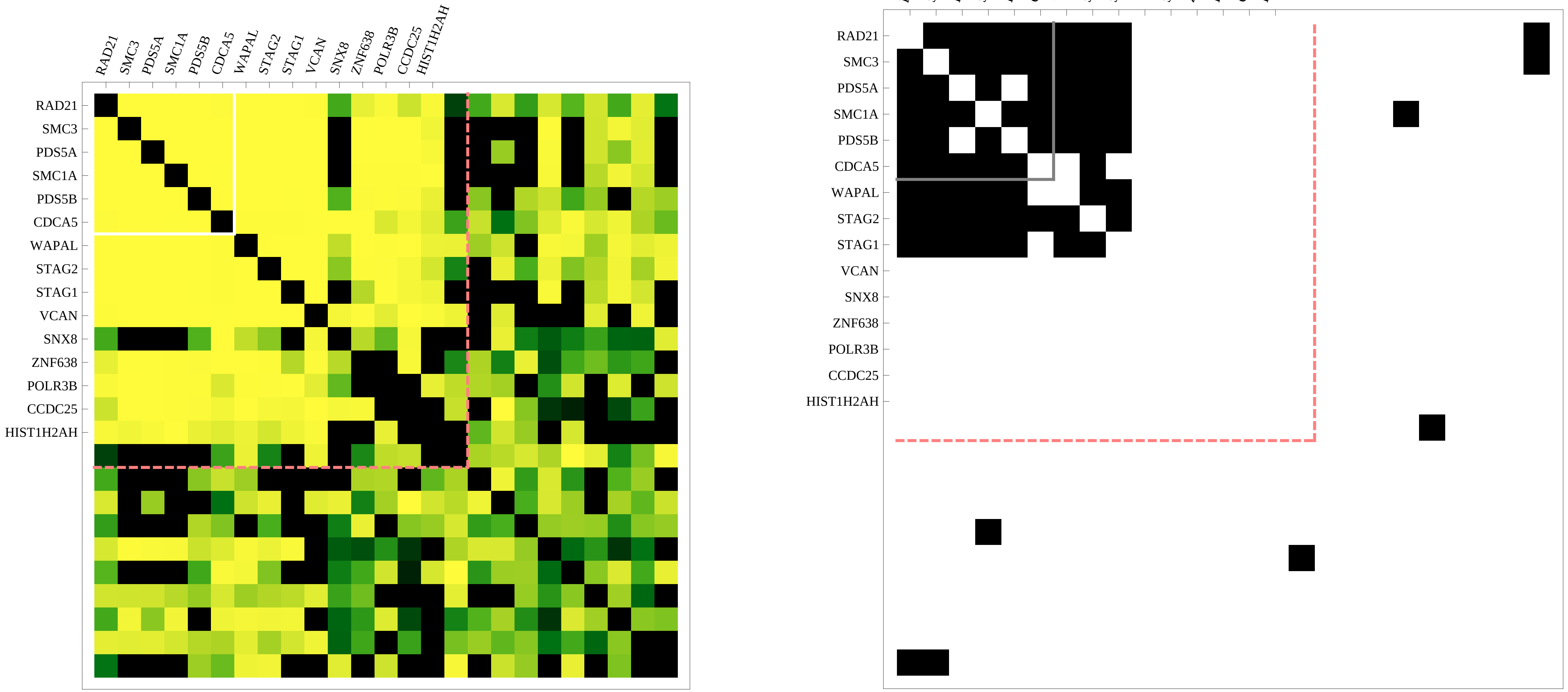

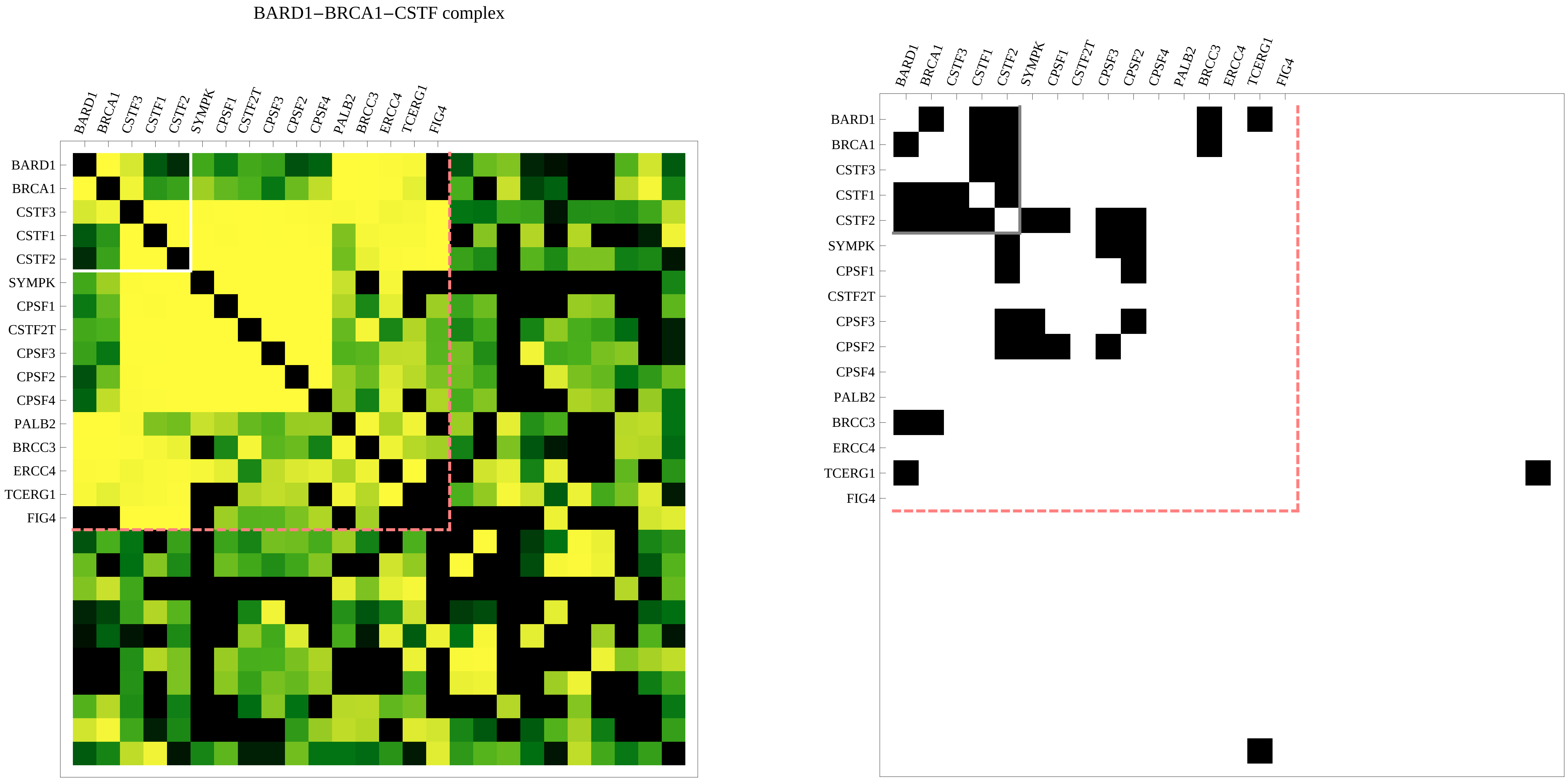
BARD1−BRCA1−CSTF complex
BARD1
BRCA1
CSTF3
CSTF1
CSTF2
SYMPK
CPSF1
CSTF2T
CPSF3
CPSF2
CPSF4
PALB2
BRCC3
ERCC4
TCERG1
FIG4

BLM complex II

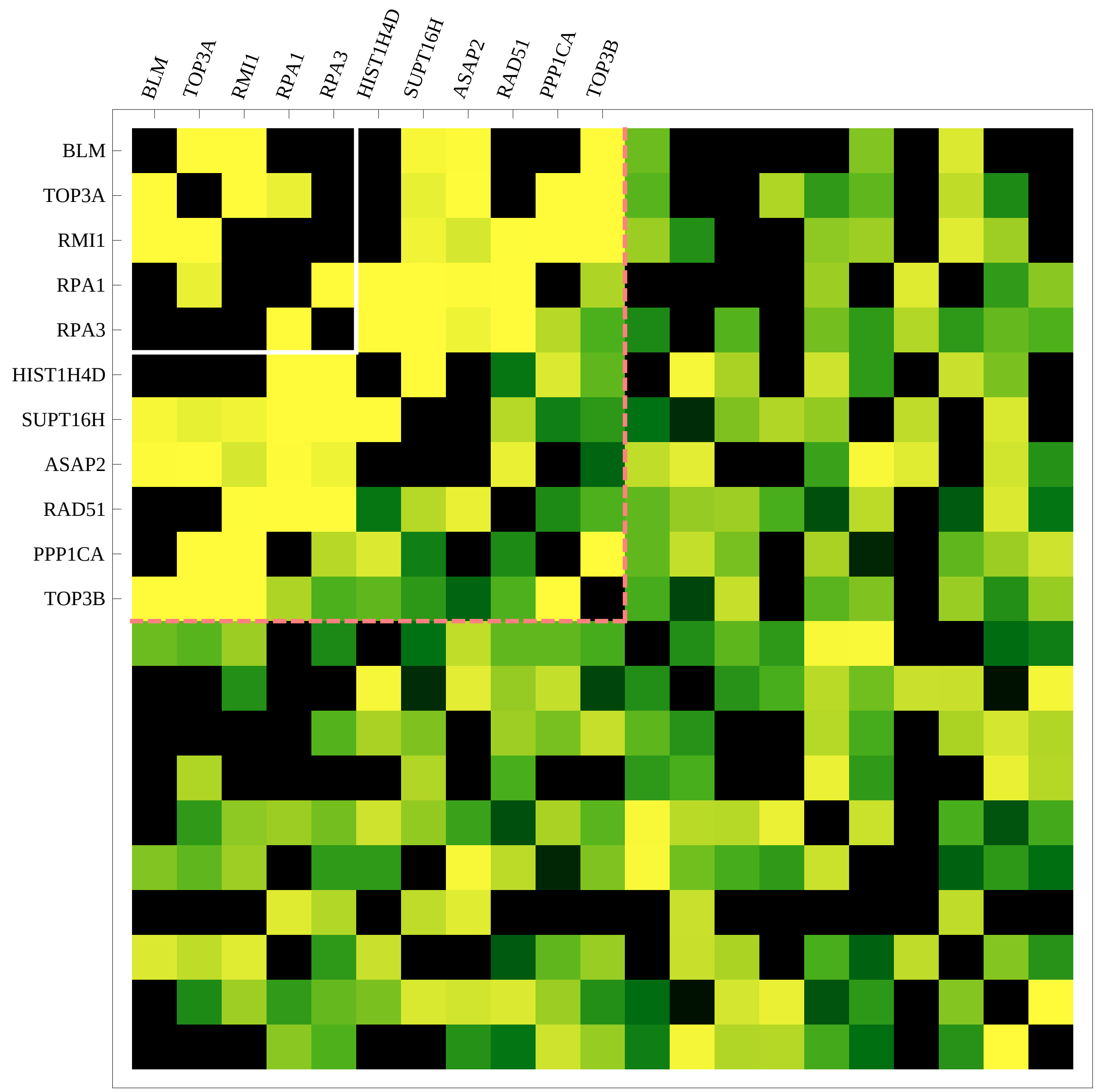

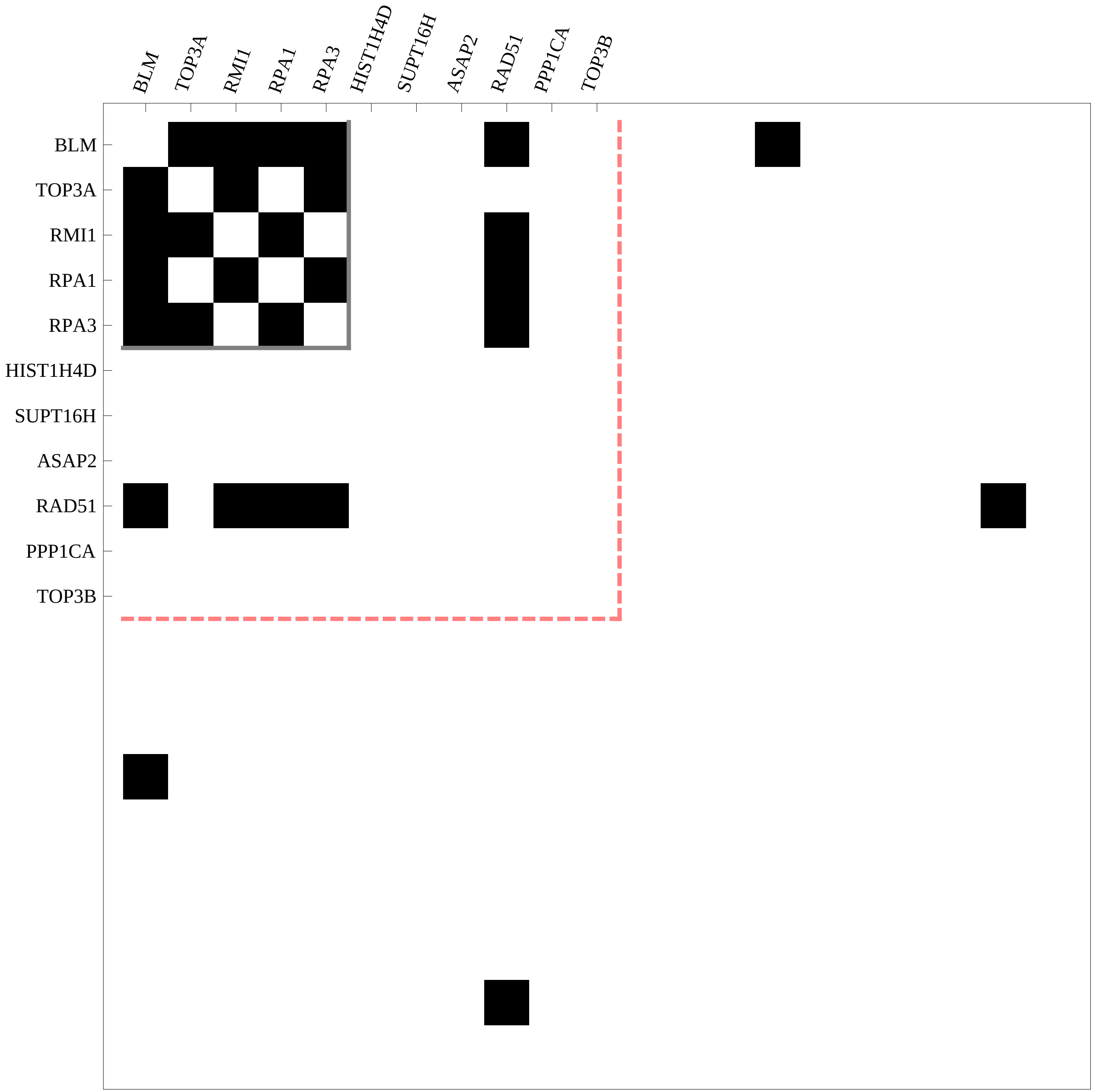

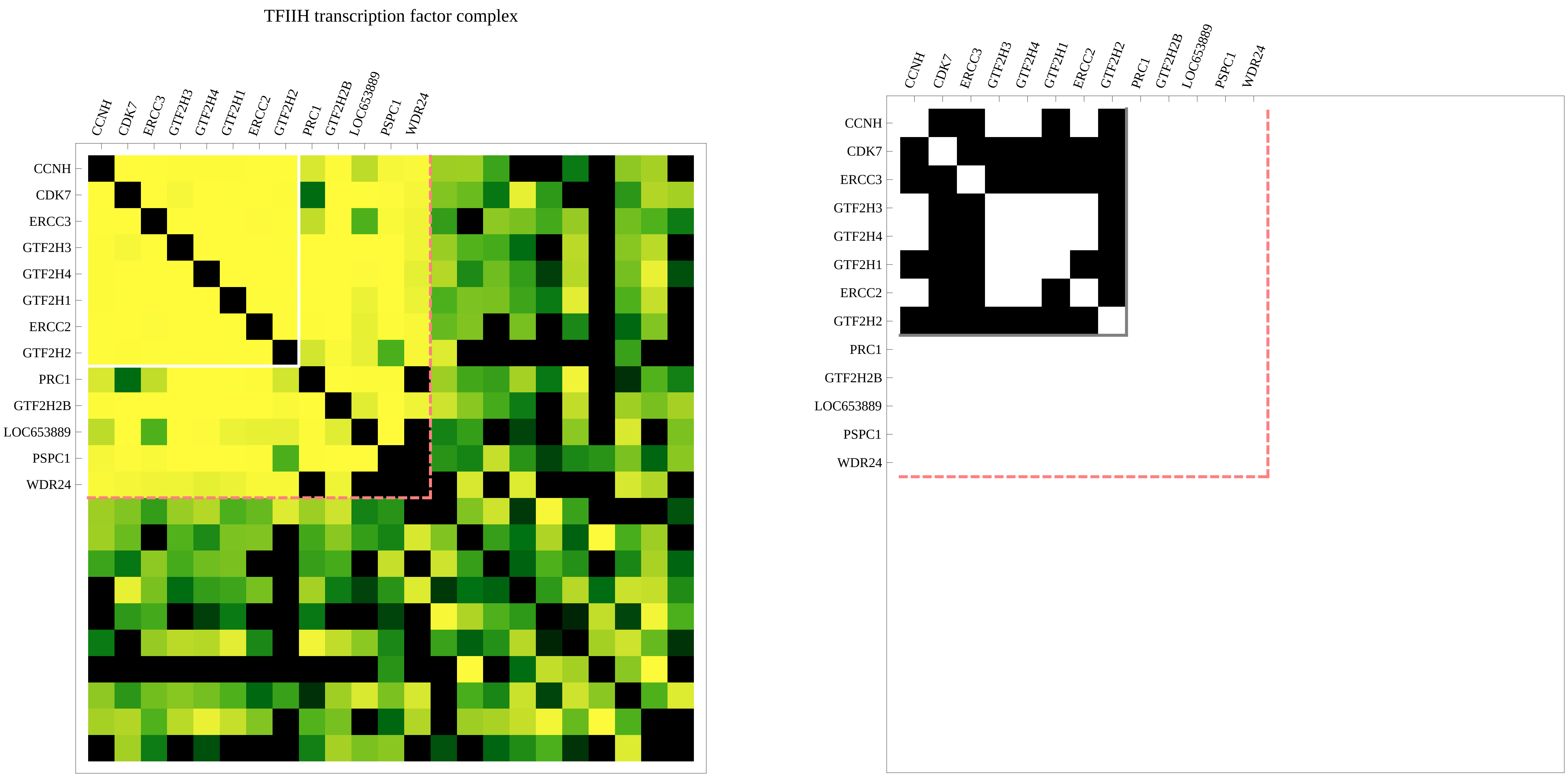
TFIIH transcription factor complex
CCNH
CDK7
ERCC3
GTF2H3
GTF2H4
GTF2H1
ERCC2
GTF2H2
PRC1
GTF2H2B
LOC653889
PSPC1
WDR24

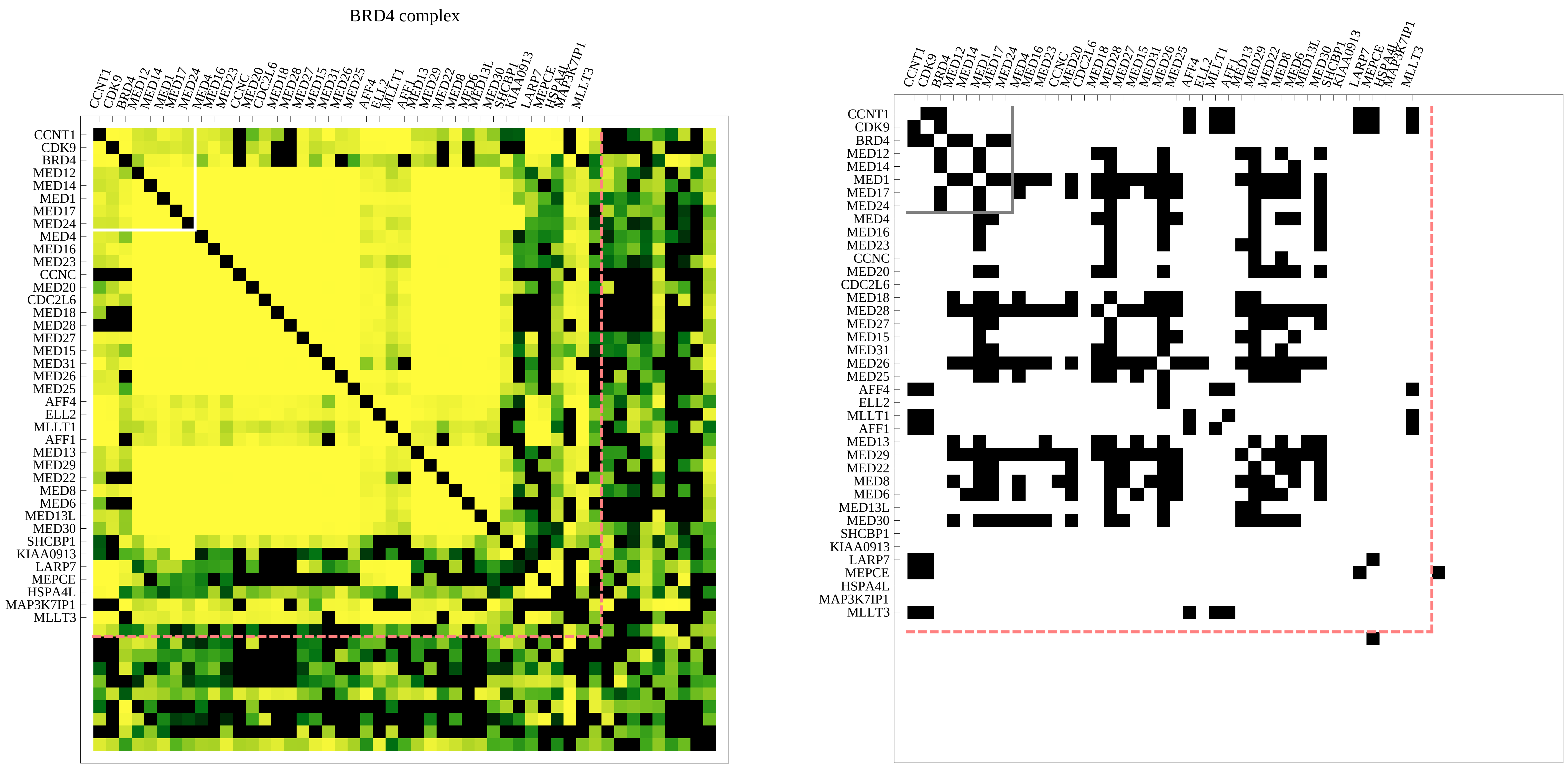
BRD4 complex
CCNT1
CDK9
BRD4
MED12
MED14
MED1
MED17
MED24
MED4
MED16
MED23
CCNC
MED20
CDC2L6
MED18
MED28
MED27
MED15
MED31
MED26
MED25
AFF4
ELL2
MLLT1
AFF1
MED13
MED29
MED22
MED8
MED6
MED13L
MED30
SHCBP1
KIAA0913
LARP7
MEPCE
HSPA4L
MAP3K7IP1
MLLT3

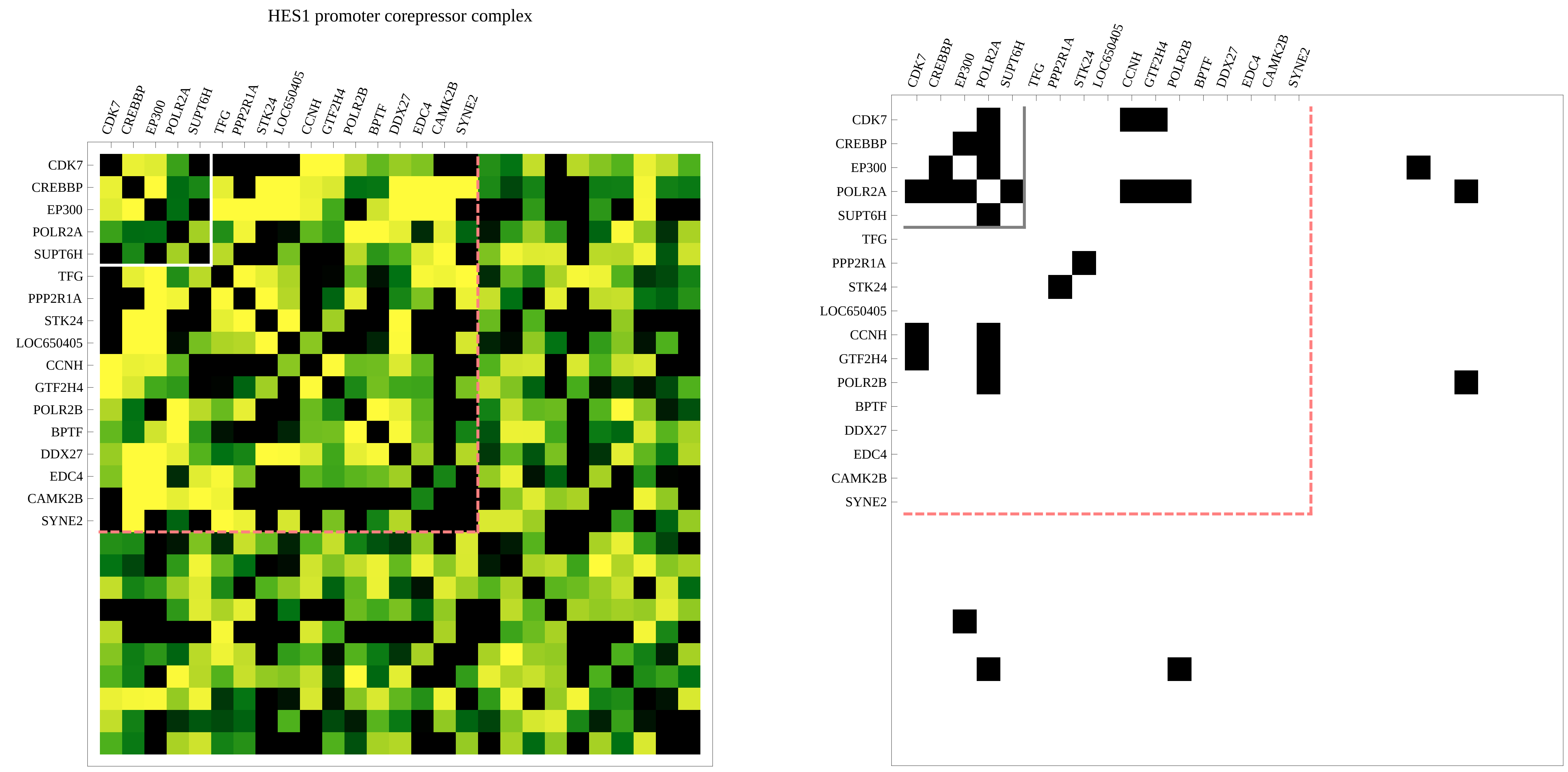

HES1 promoter corepressor complex
CDK7
CREBBP
EP300
POLR2A
SUPT6H
TFG
PPP2R1A
STK24
LOC650405
CCNH
GTF2H4
POLR2B
BPTF
DDX27
EDC4
CAMK2B
SYNE2

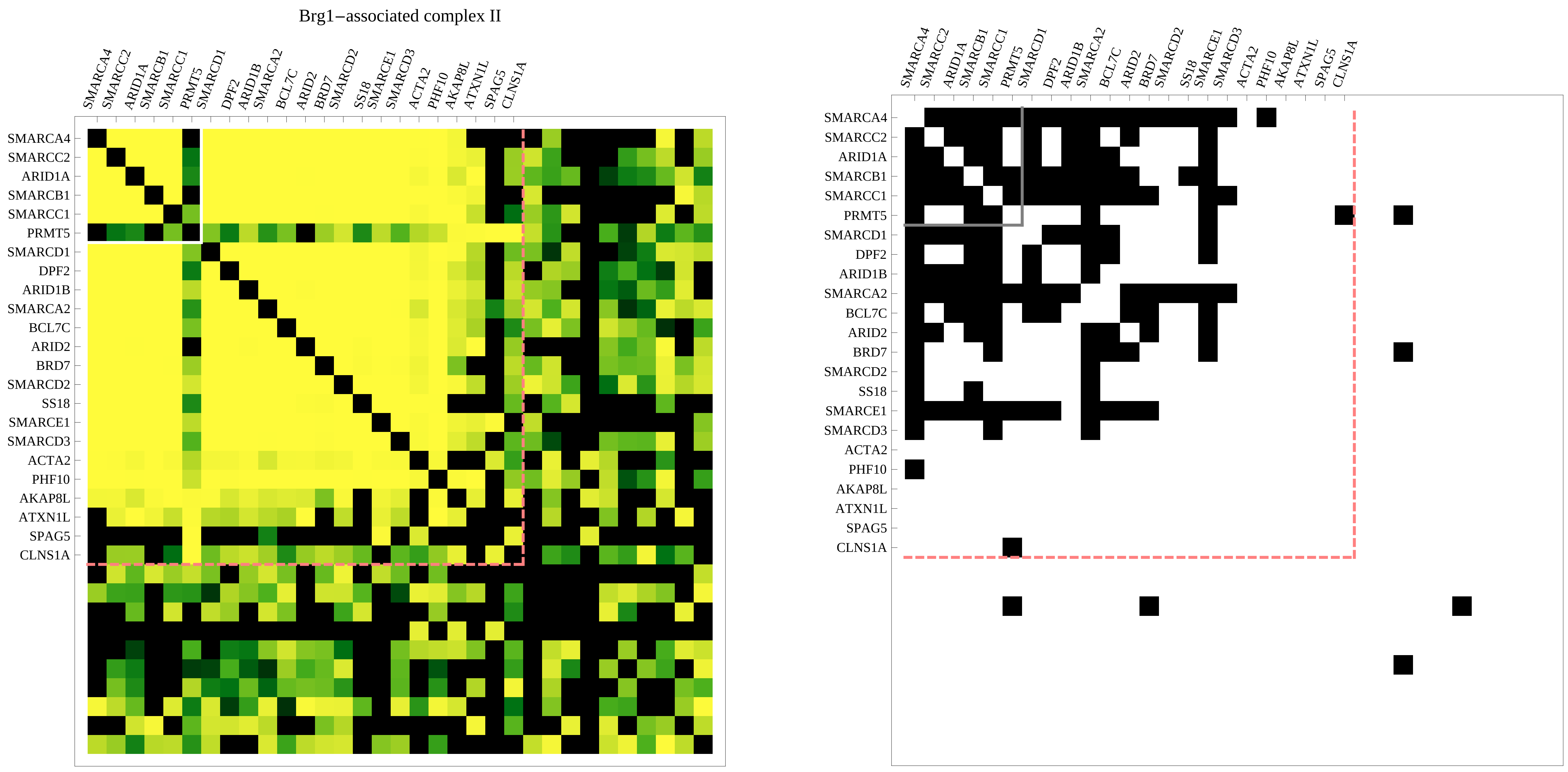

Brg1−associated complex II
SMARCA4
SMARCC2
ARID1A
SMARCB1
SMARCC1
PRMT5
SMARCD1
DPF2
ARID1B
SMARCA2
BCL7C
ARID2
BRD7
SMARCD2
SS18
SMARCE1
SMARCD3
ACTA2
PHF10
AKAP8L
ATXN1L
SPAG5
CLNS1A

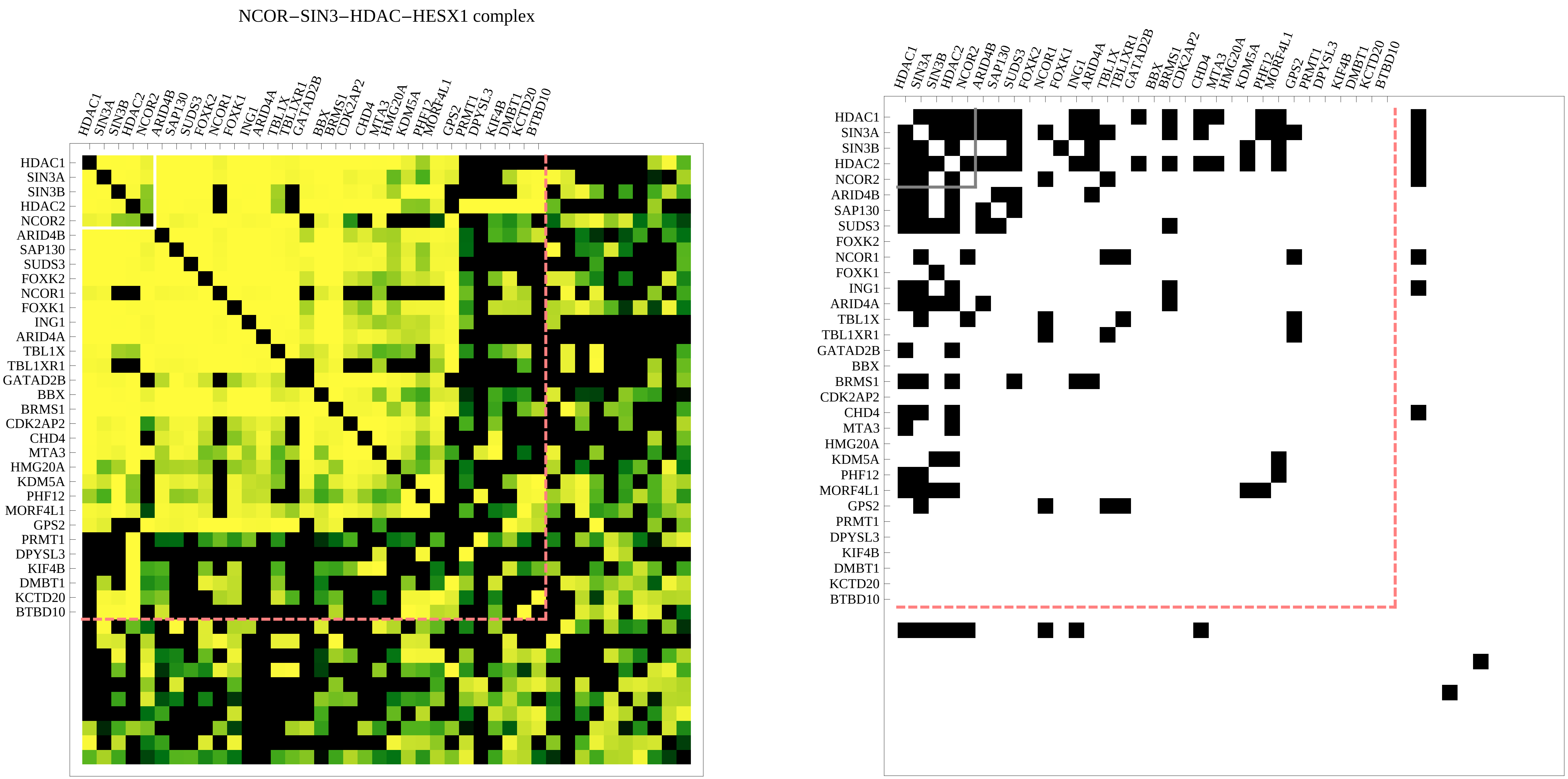
NCOR−SIN3−HDAC−HESX1 complex
HDAC1
SIN3A
SIN3B
HDAC2
NCOR2
ARID4B
SAP130
SUDS3
FOXK2
NCOR1
FOXK1
ING1
ARID4A
TBL1X
TBL1XR1
GATAD2B
BBX
BRMS1
CDK2AP2
CHD4
MTA3
HMG20A
KDM5A
PHF12
MORF4L1
GPS2
PRMT1
DPYSL3
KIF4B
DMBT1
KCTD20
BTBD10

SMN complex (GEMIN5,2,3,4, SMN)

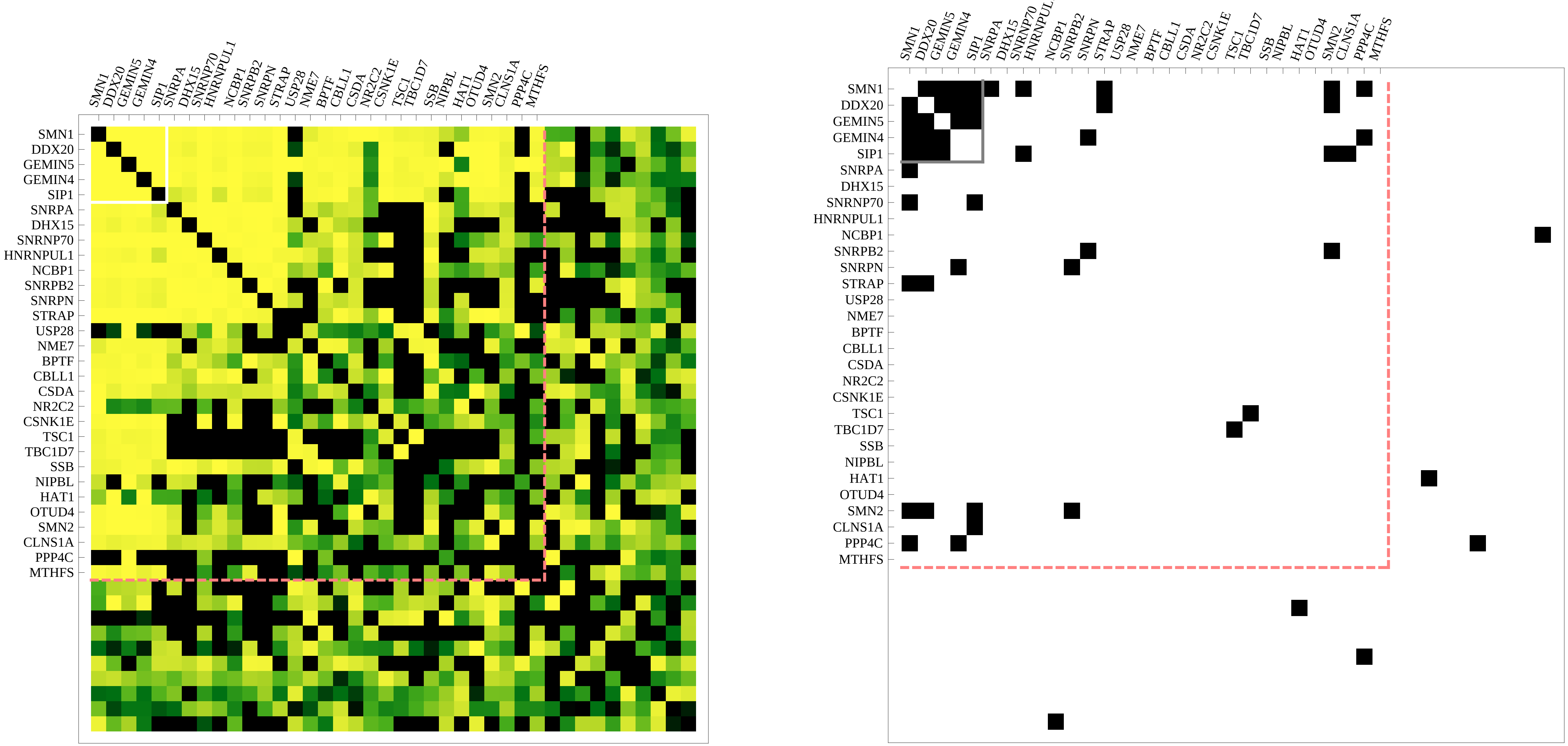

EIF3 complex (EIF3A, EIF3B, EIF3G, EIF3I, EIF3C)

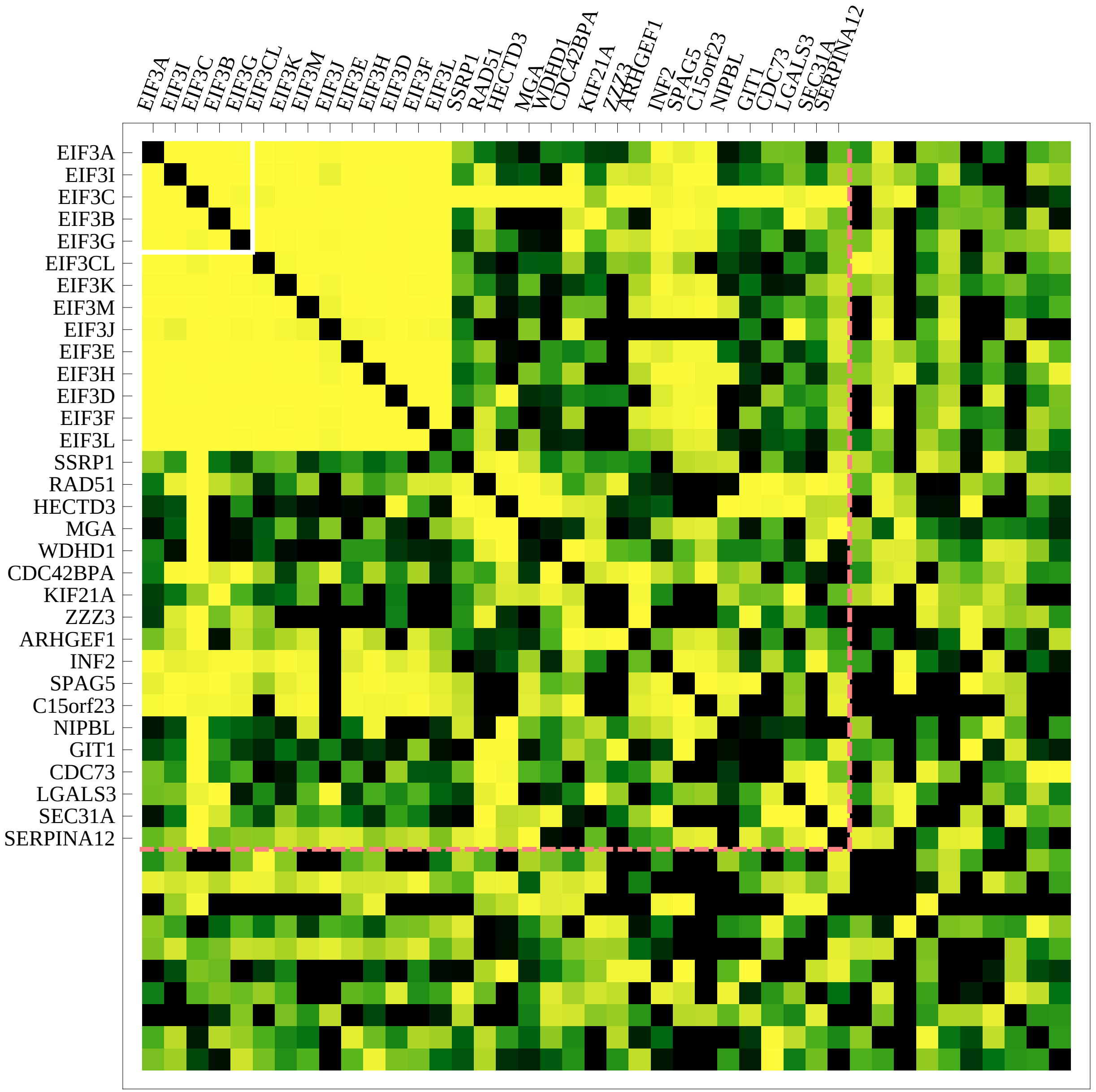

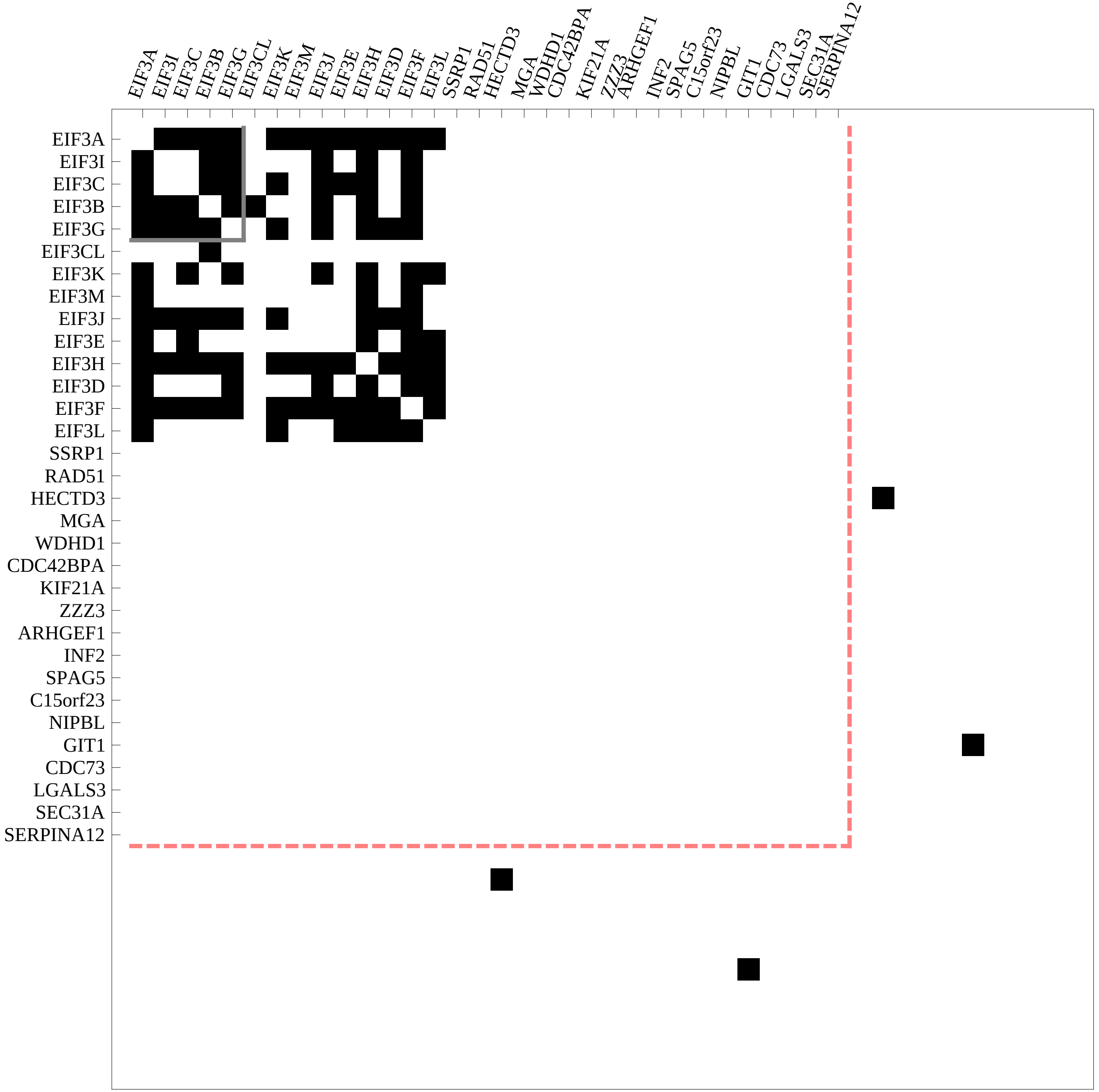

EIF3 complex (EIF3A, EIF3B, EIF3G, EIF3I, EIF3J)

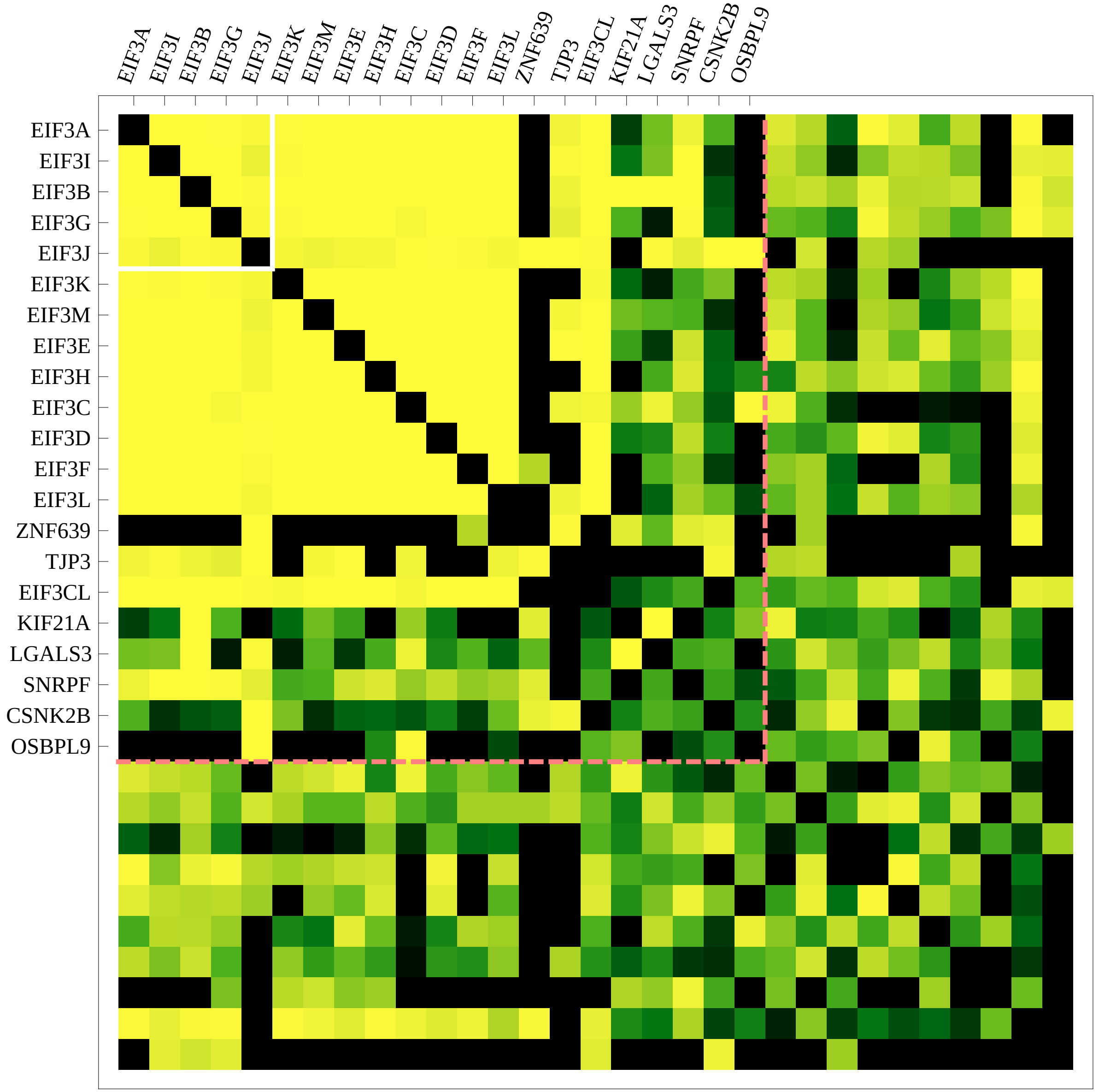

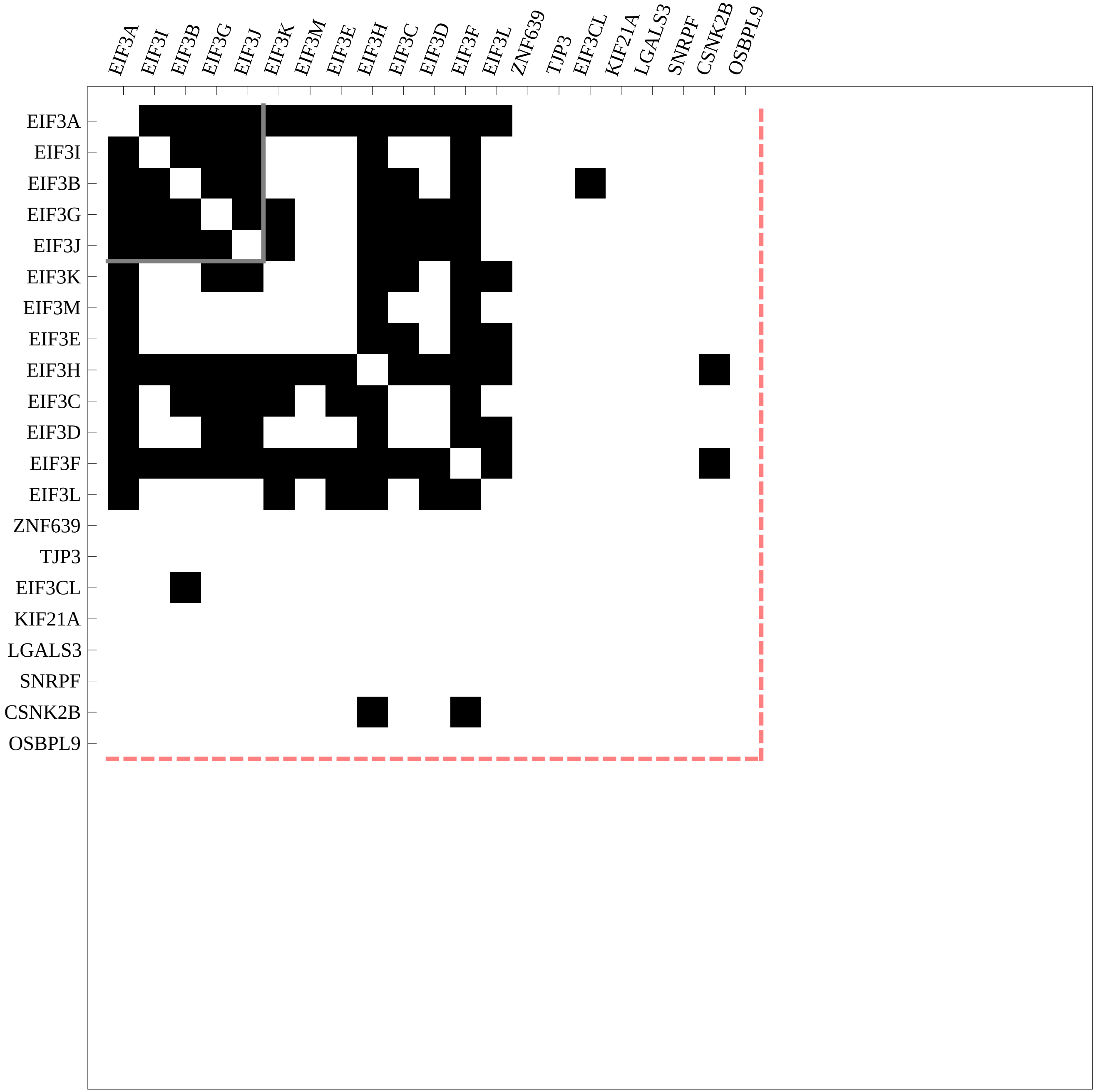

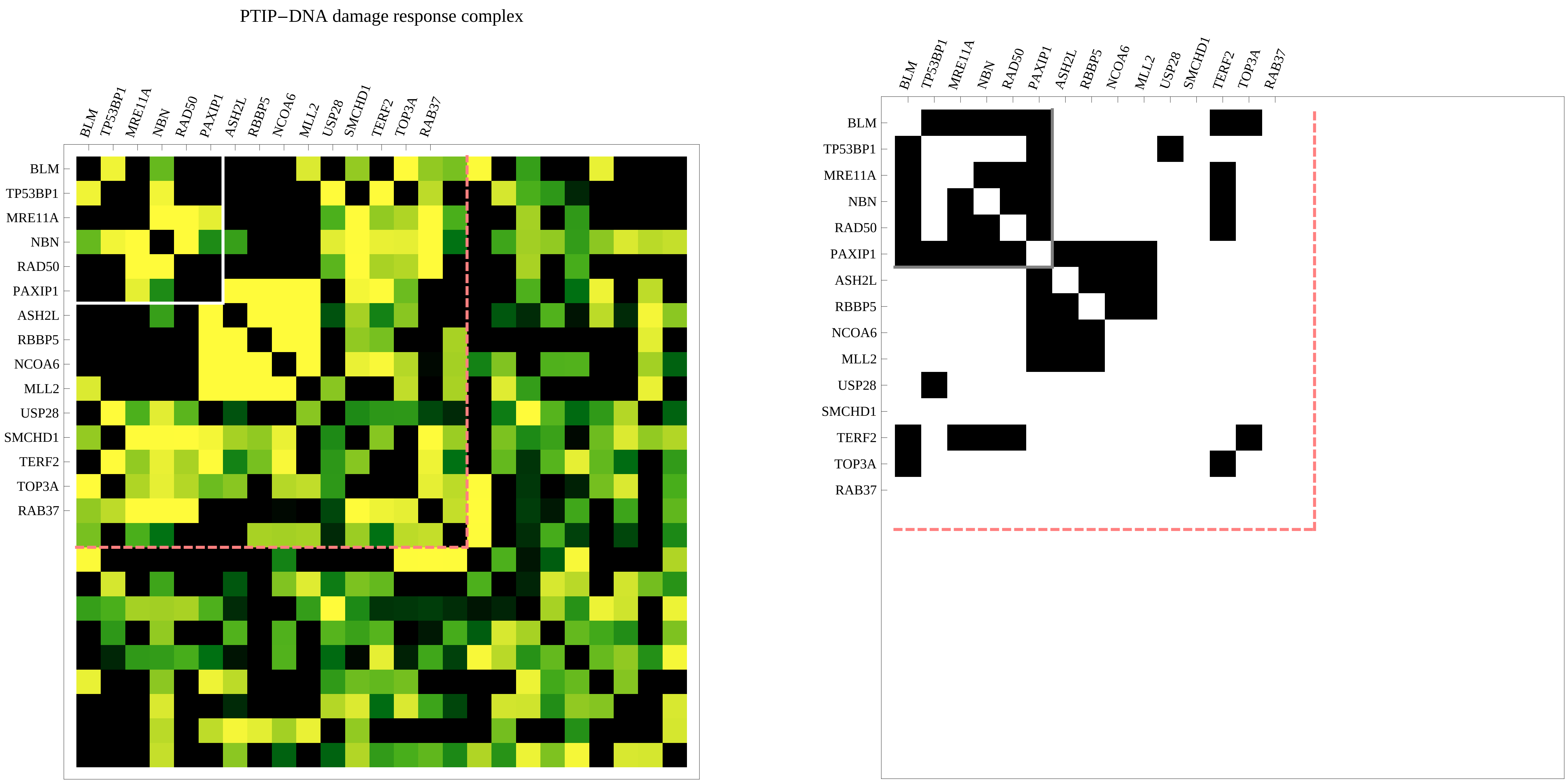
PTIP−DNA damage response complex
BLM
TP53BP1
MRE11A
NBN
RAD50
PAXIP1
ASH2L
RBBP5
NCOA6
MLL2
USP28
SMCHD1
TERF2
TOP3A
RAB37

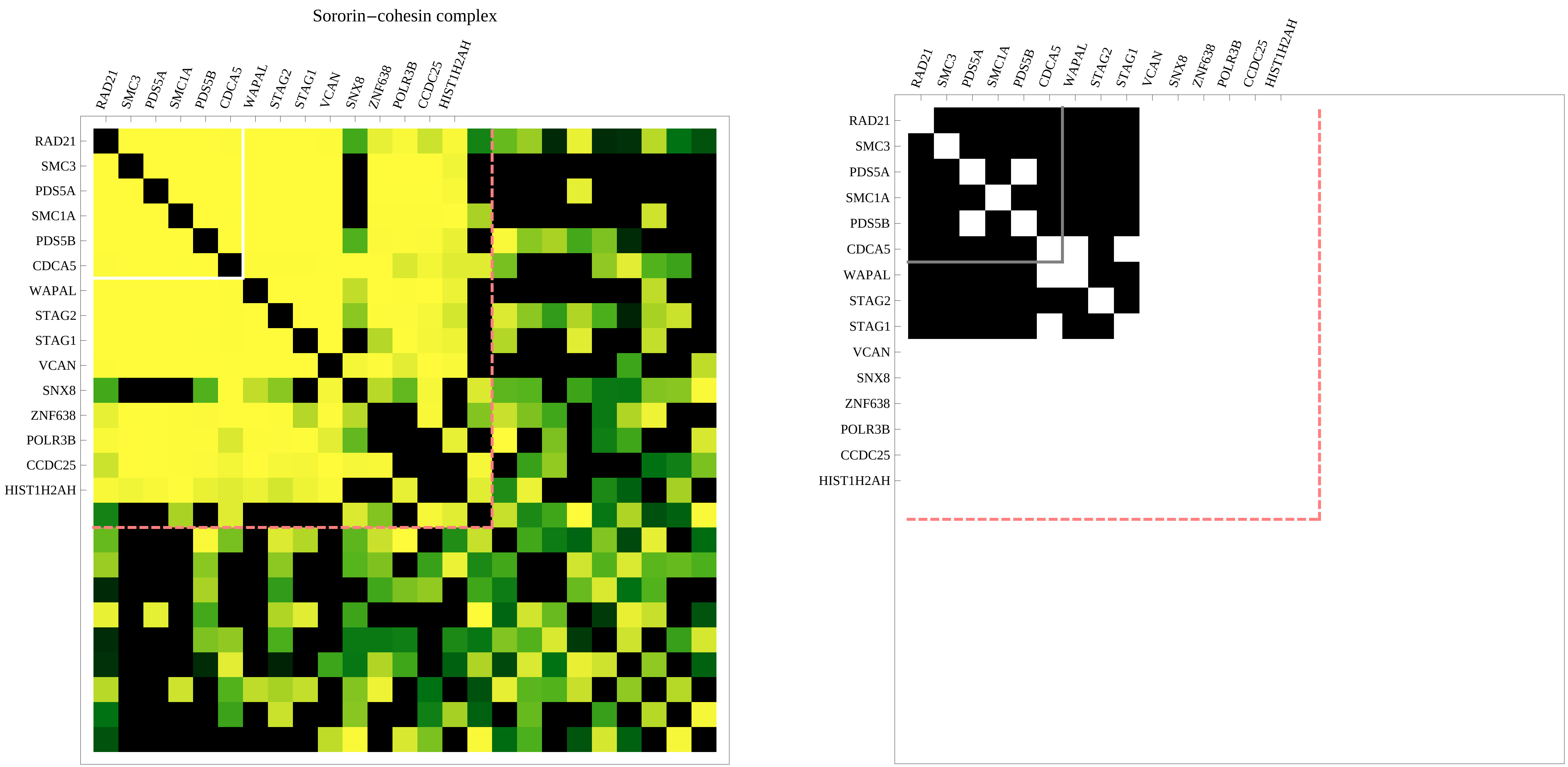
Sororin−cohesin complex
RAD21
SMC3
PDS5A
SMC1A
PDS5B
CDCA5
WAPAL
STAG2
STAG1
VCAN
SNX8
ZNF638
POLR3B
CCDC25
HIST1H2AH

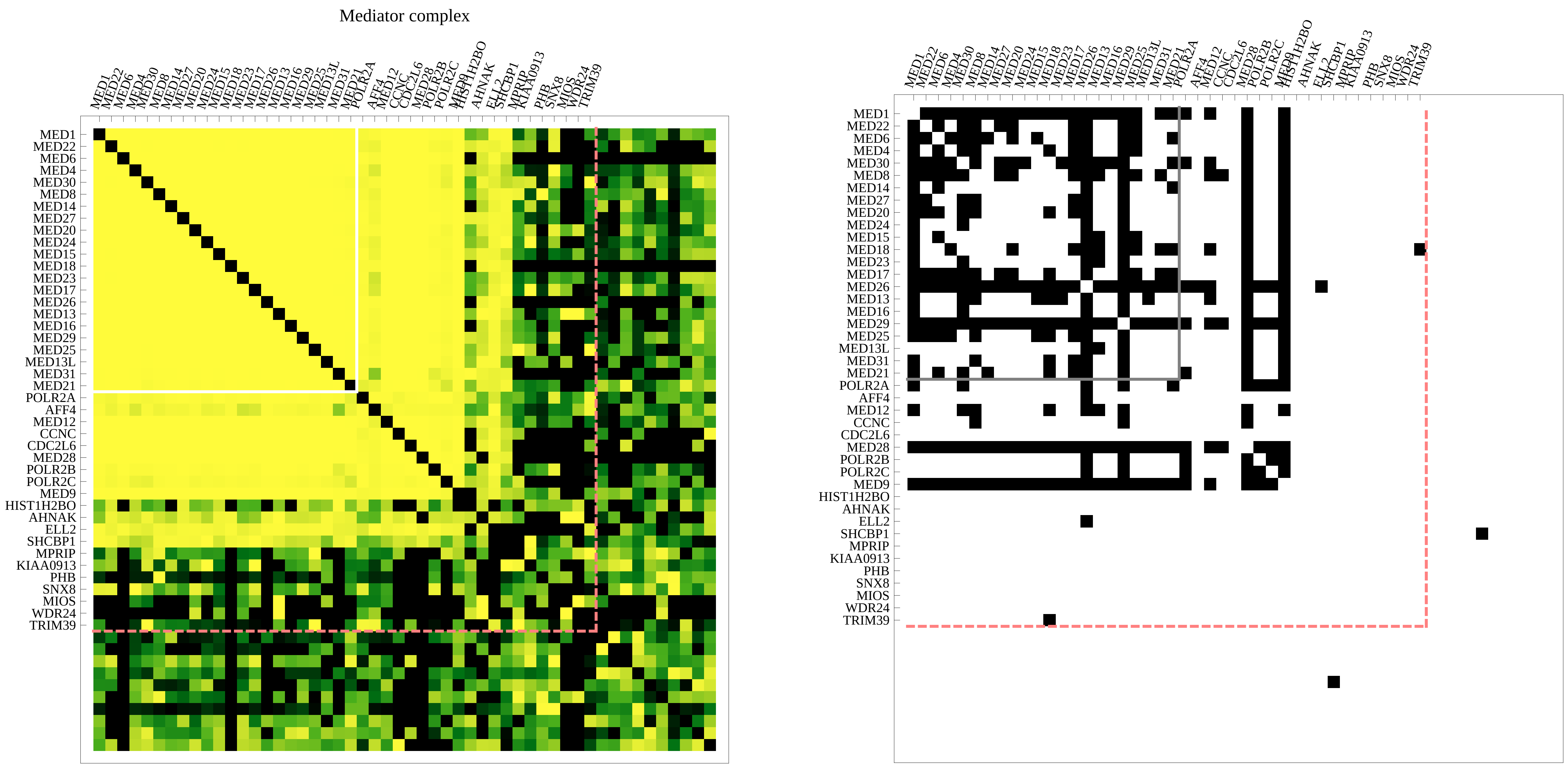

Mediator complex
MED1
MED22
MED6
MED4
MED30
MED8
MED14
MED27
MED20
MED24
MED15
MED18
MED23
MED17
MED26
MED13
MED16
MED29
MED25
MED13L
MED31
MED21
POLR2A
AFF4
MED12
CCNC
CDC2L6
MED28
POLR2B
POLR2C
MED9
HIST1H2BO
AHNAK
ELL2
SHCBP1
MPRIP
KIAA0913
PHB
SNX8
MIOS
WDR24
TRIM39

26S proteasome

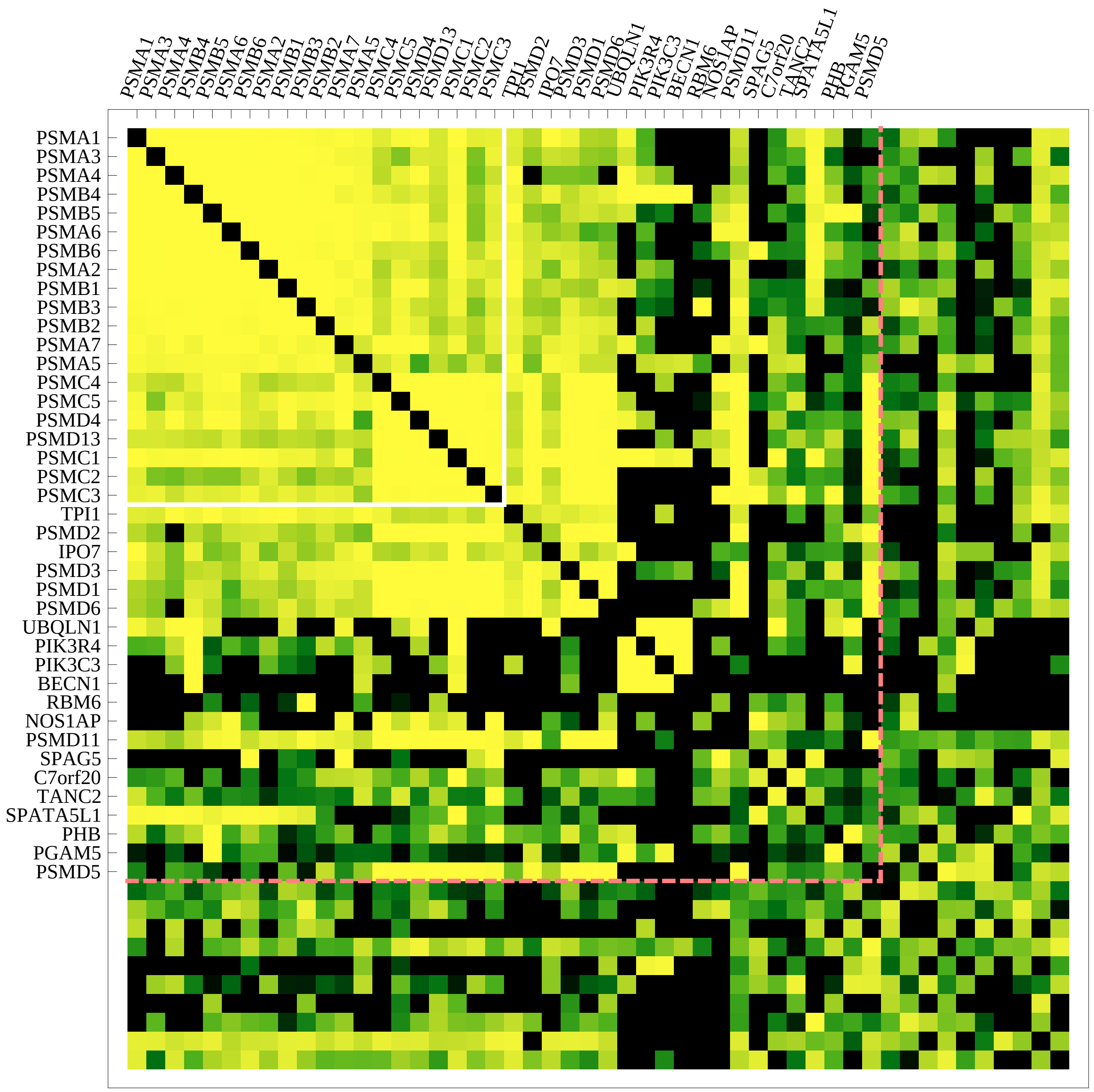

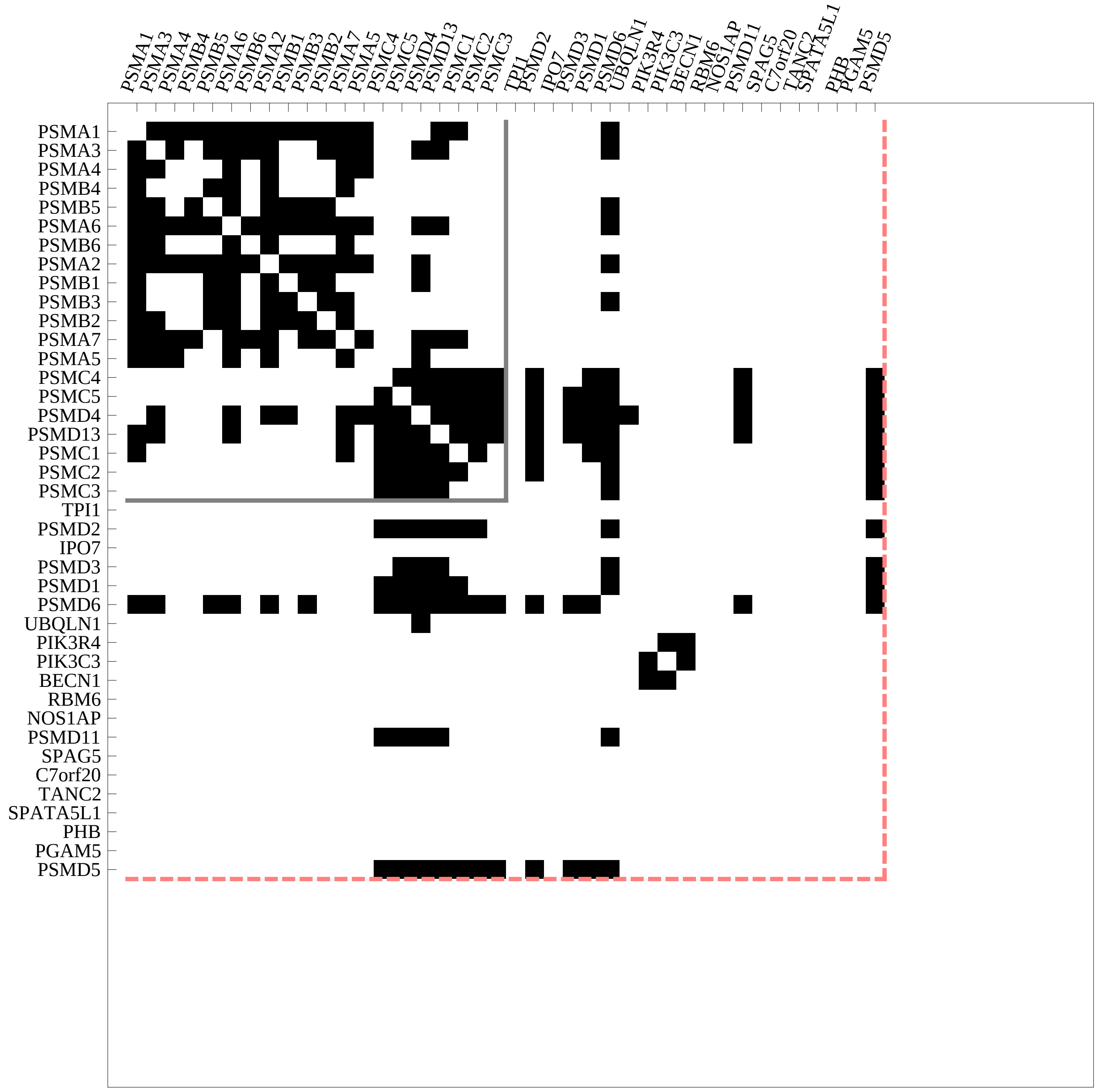

TFIIH transcription factor complex (ERCC2, ERCC3, GTF2H1, CDK7, CCNH, GTF2H

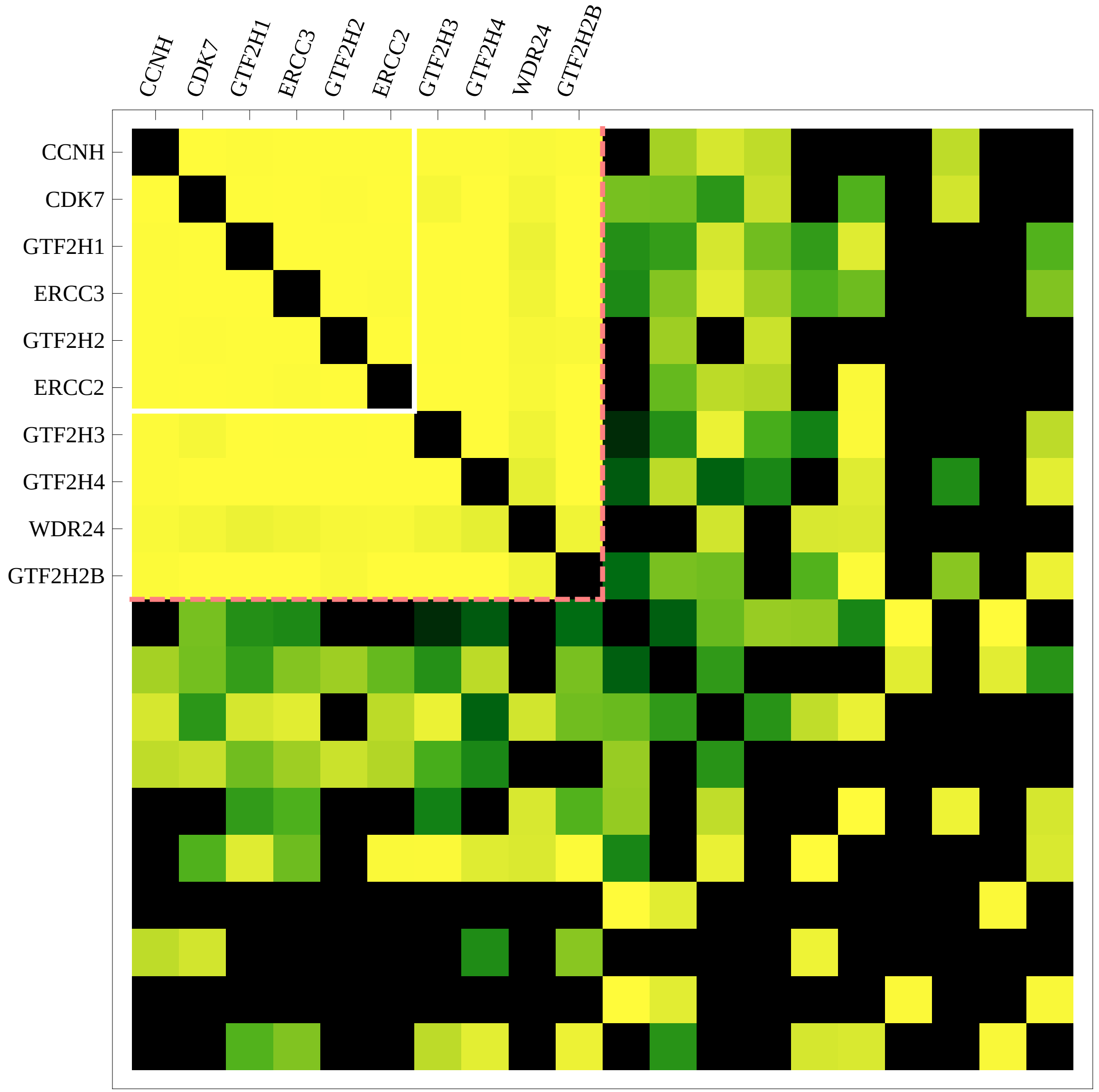

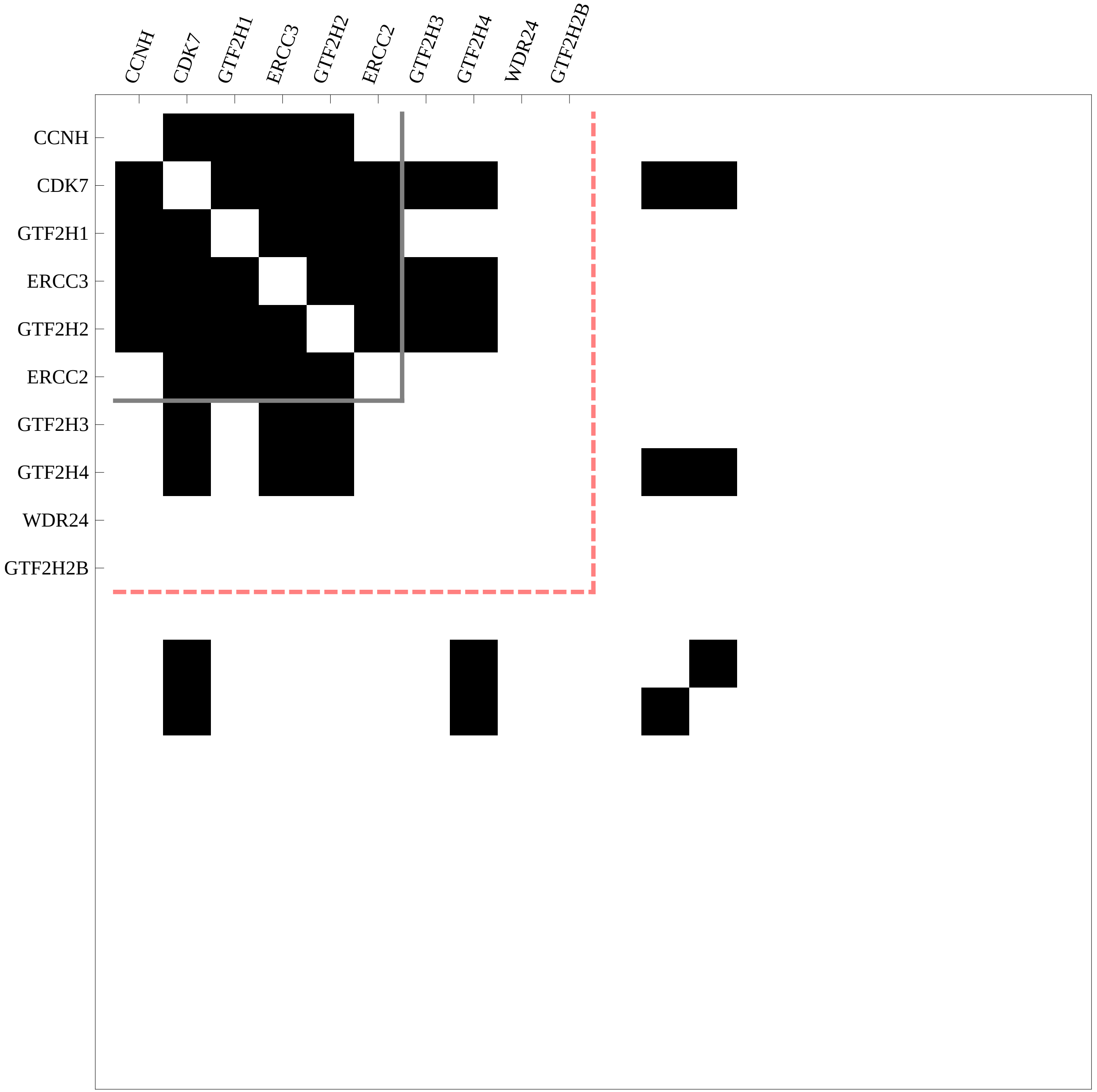

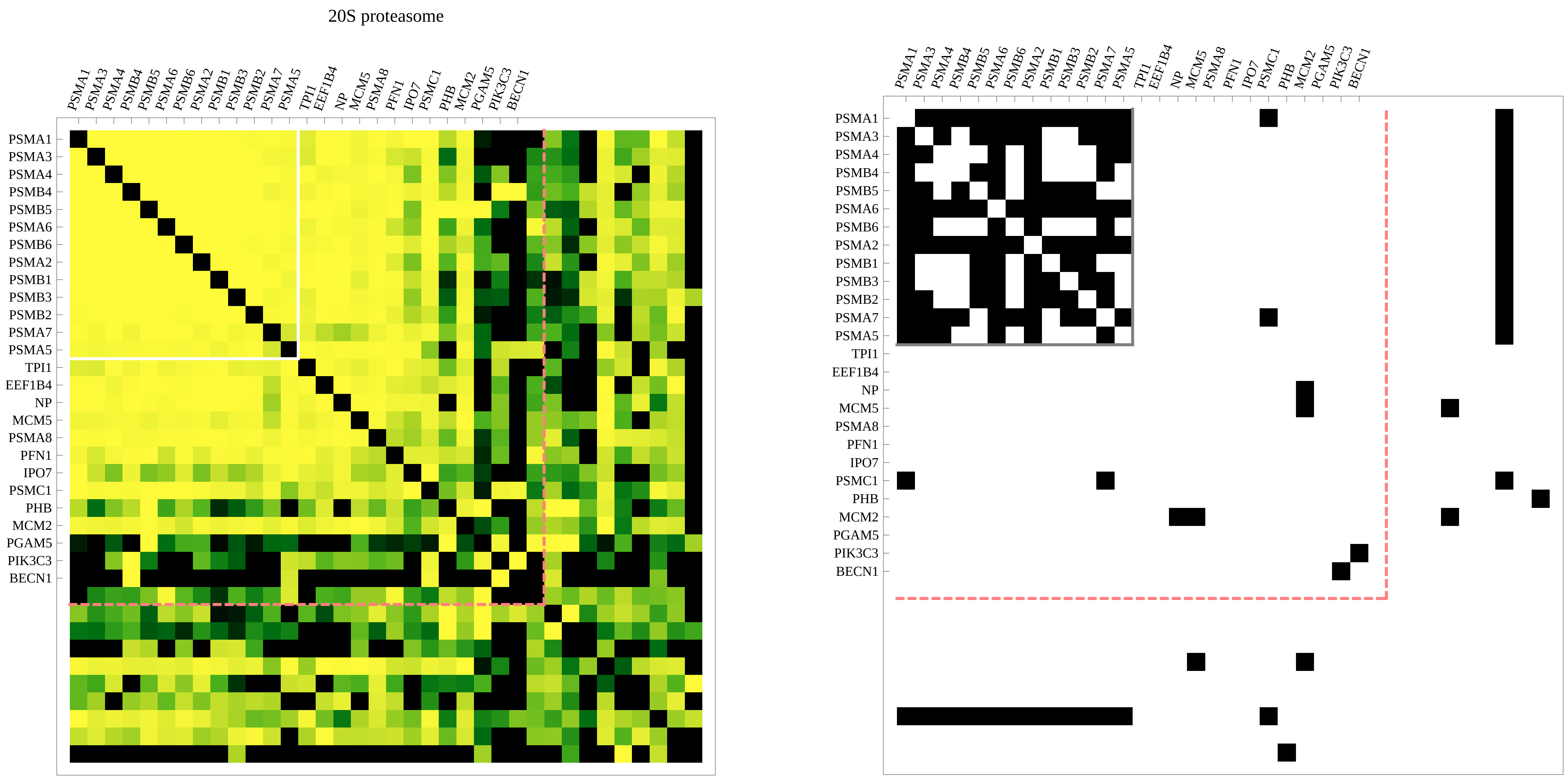

20S proteasome
PSMA1
PSMA3
PSMA4
PSMB4
PSMB5
PSMA6
PSMB6
PSMA2
PSMB1
PSMB3
PSMB2
PSMA7
PSMA5
TPI1
EEF1B4
NP
MCM5
PSMA8
PFN1
IPO7
PSMC1
PHB
MCM2
PGAM5
PIK3C3
BECN1

PA28−20S proteasome

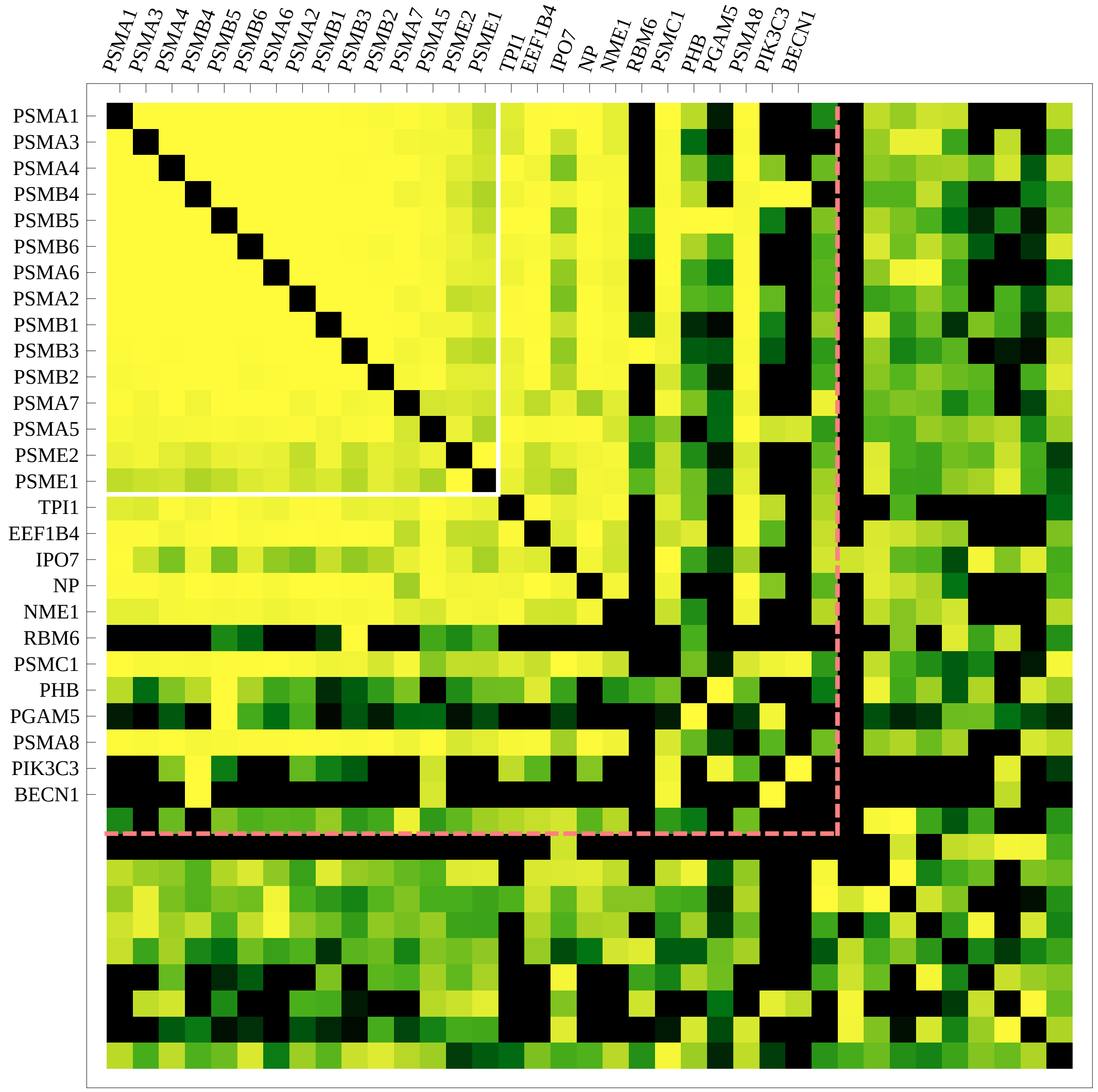

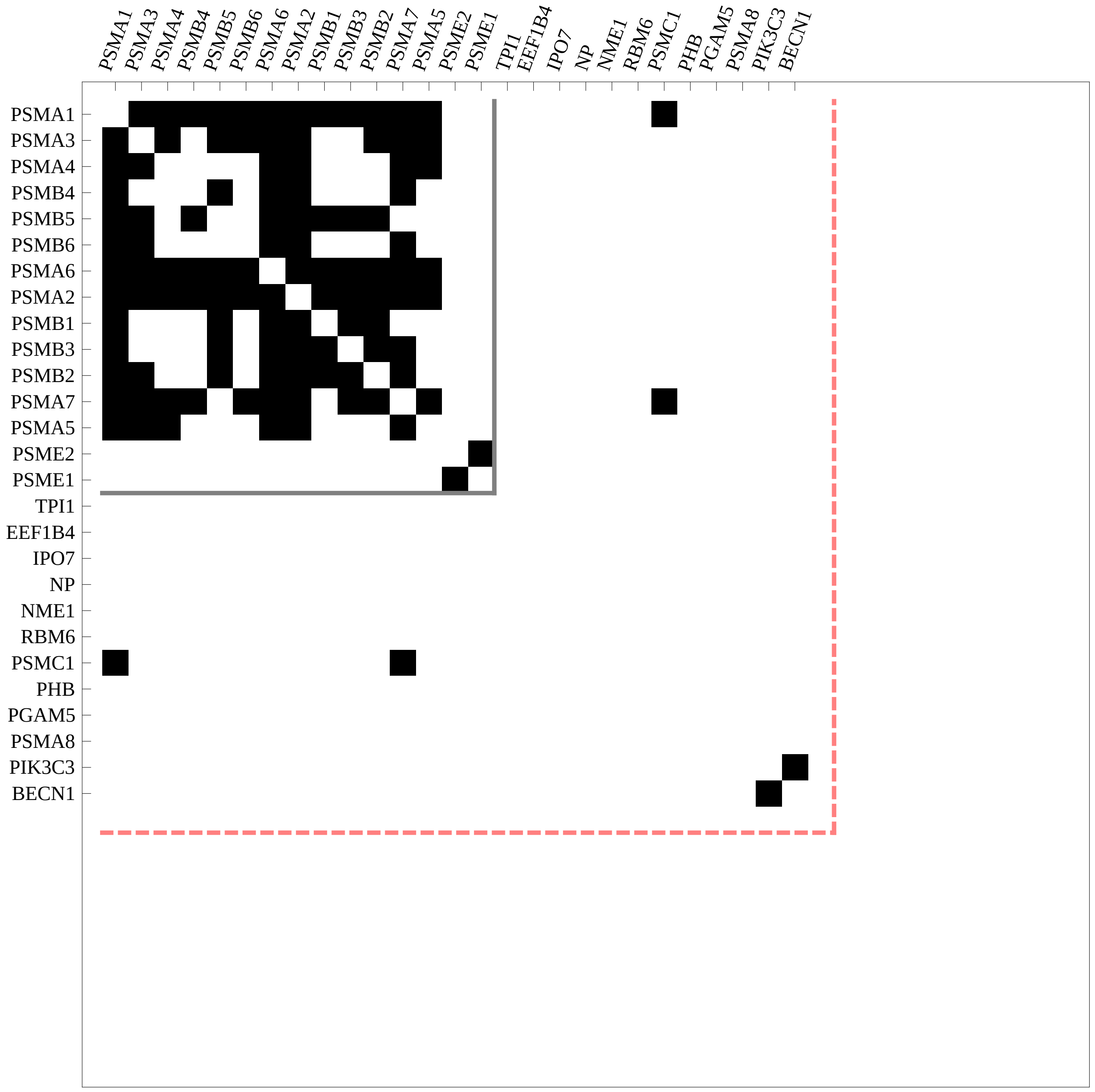

PA28gamma−20S proteasome

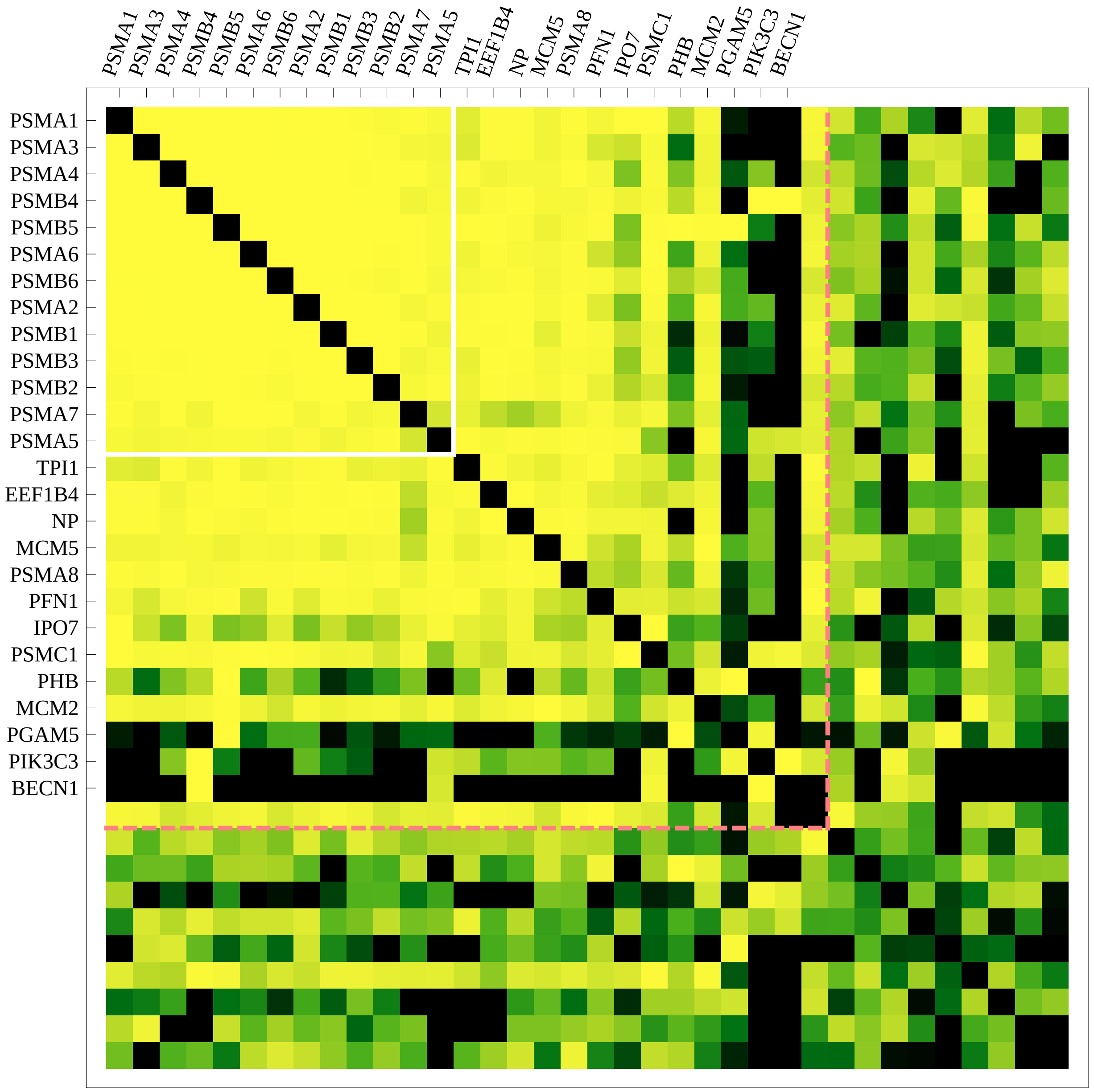

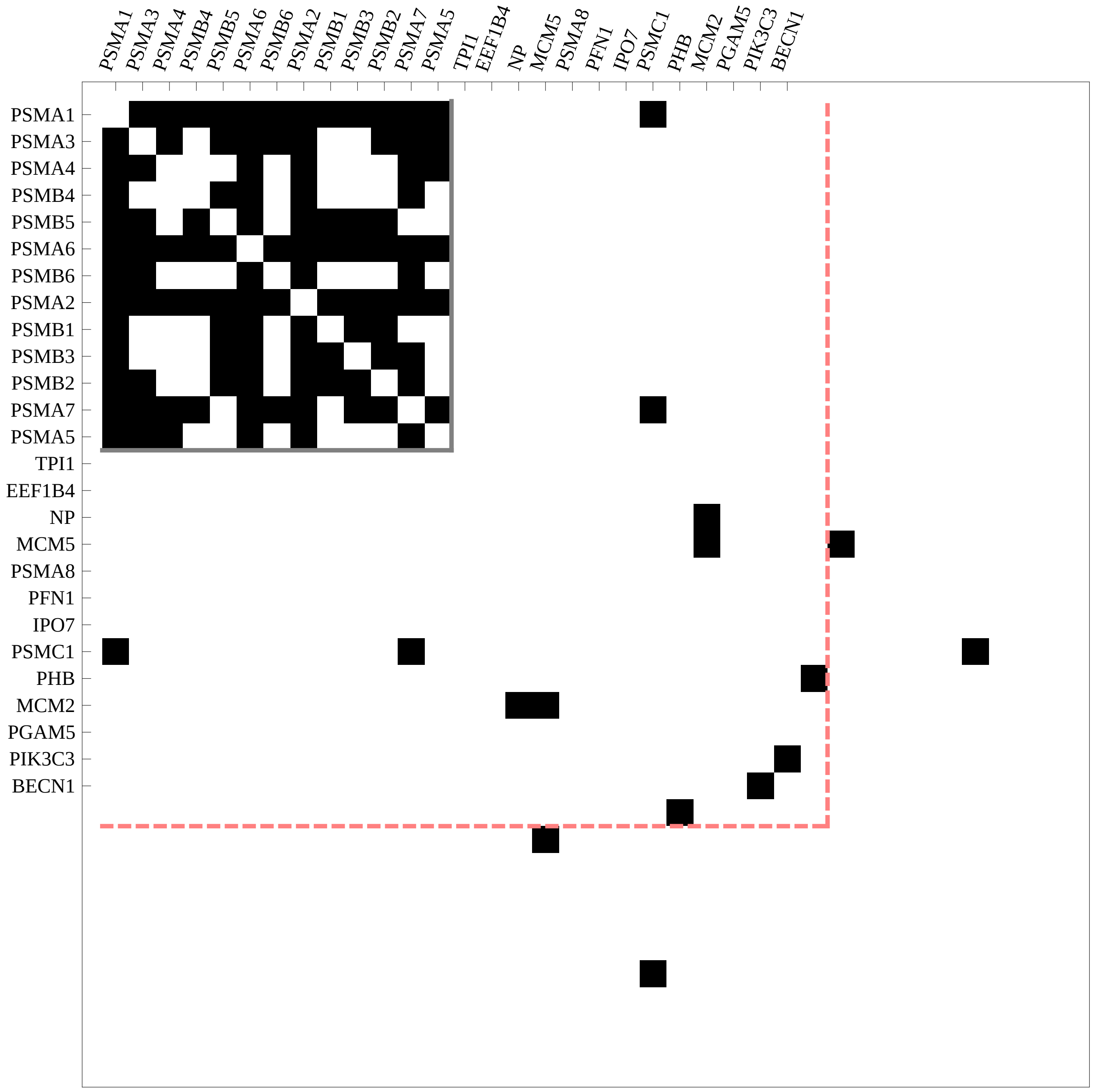

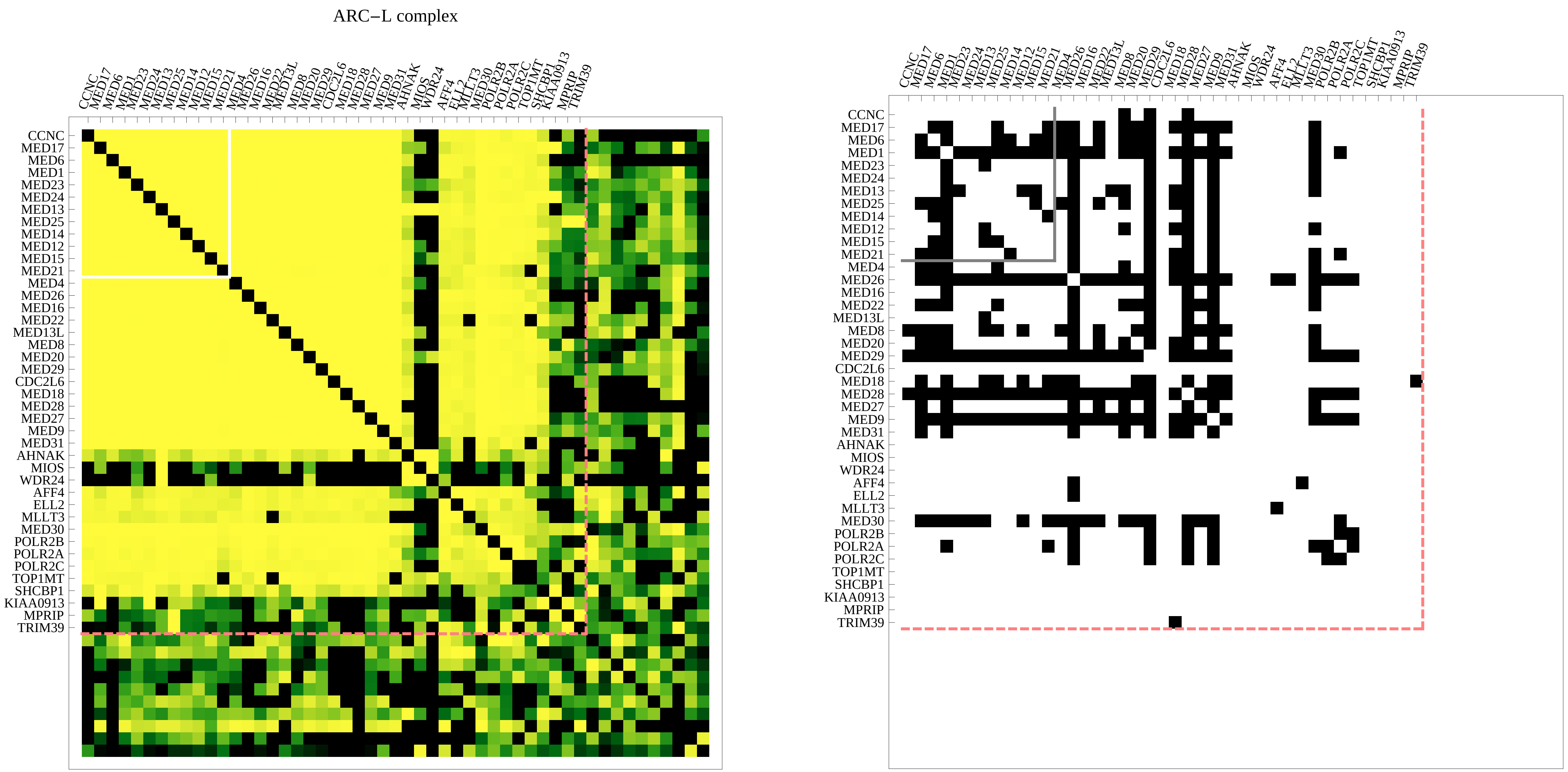
ARC−L complex
CCNC
MED17
MED6
MED1
MED23
MED24
MED13
MED25
MED14
MED12
MED15
MED21
MED4
MED26
MED16
MED22
MED13L
MED8
MED20
MED29
CDC2L6
MED18
MED28
MED27
MED9
MED31
AHNAK
MIOS
WDR24
AFF4
ELL2
MLLT3
MED30
POLR2B
POLR2A
POLR2C
TOP1MT
SHCBP1
KIAA0913
MPRIP
TRIM39

PC2 complex

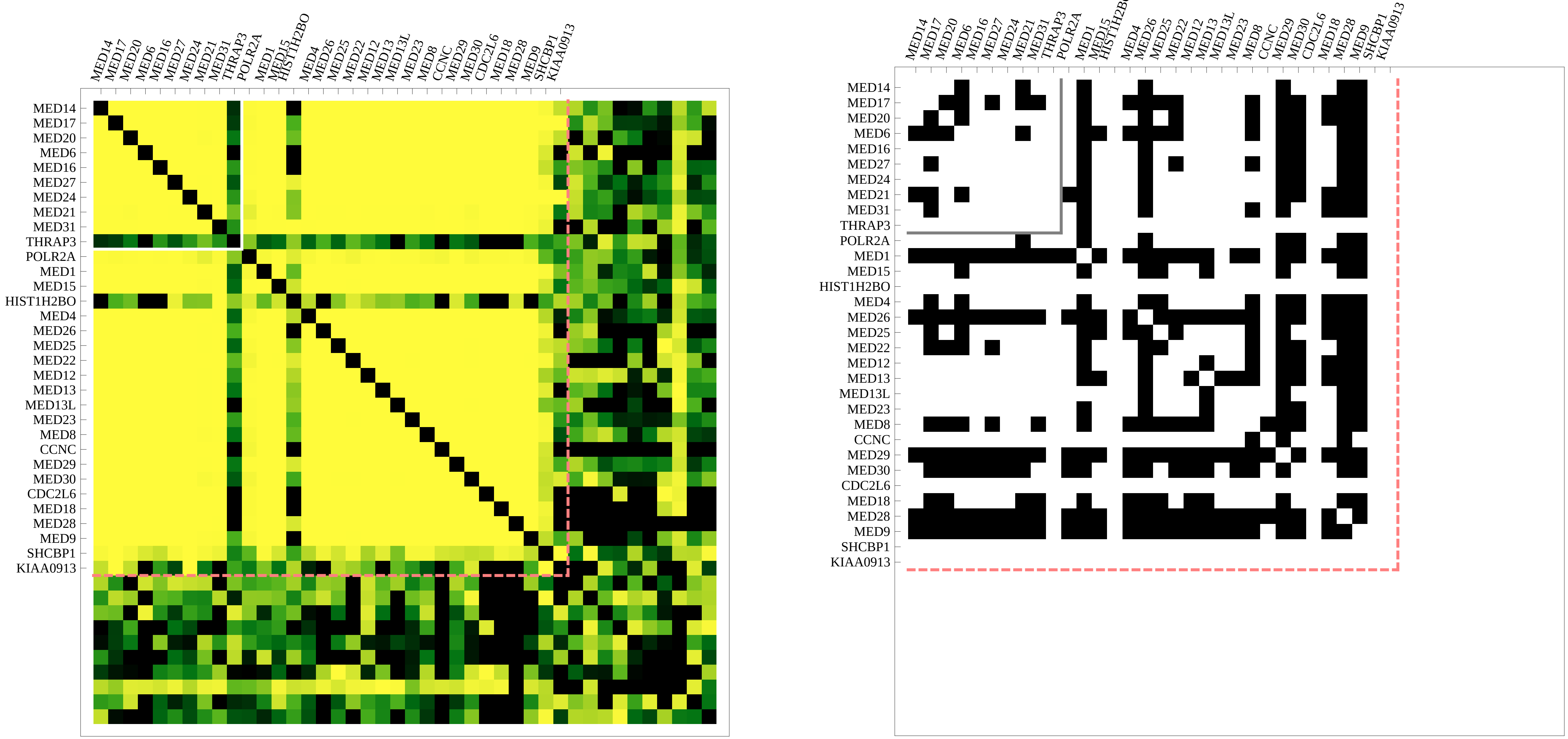
MED14
MED17
MED20
MED6
MED16
MED27
MED24
MED21
MED31
THRAP3
POLR2A
MED1
MED15
HIST1H2BO
MED4
MED26
MED25
MED22
MED12
MED13
MED13L
MED23
MED8
CCNC
MED29
MED30
CDC2L6
MED18
MED28
MED9
SHCBP1
KIAA0913
